# The Huanan Seafood Wholesale Market in Wuhan was the early epicenter of the COVID-19 pandemic

**DOI:** 10.1126/science.abp8715

**Published:** 2022-07-26

**Authors:** Michael Worobey, Joshua I. Levy, Lorena Malpica Serrano, Alexander Crits-Christoph, Jonathan E. Pekar, Stephen A. Goldstein, Angela L. Rasmussen, Moritz U. G. Kraemer, Chris Newman, Marion P. G. Koopmans, Marc A. Suchard, Joel O. Wertheim, Philippe Lemey, David L. Robertson, Robert F. Garry, Edward C. Holmes, Andrew Rambaut, Kristian G. Andersen

**Affiliations:** ^1^ Department of Ecology and Evolutionary Biology, University of Arizona, Tucson, AZ 85721, USA.; ^2^ Department of Immunology and Microbiology, The Scripps Research Institute, La Jolla, CA 92037, USA.; ^3^ W. Harry Feinstone Department of Molecular Microbiology and Immunology, Johns Hopkins Bloomberg School of Public Health, Baltimore, MD 21205, USA.; ^4^ Bioinformatics and Systems Biology Graduate Program, University of California San Diego, La Jolla, CA 92093, USA.; ^5^ Department of Biomedical Informatics, University of California San Diego, La Jolla, CA 92093, USA.; ^6^ Department of Human Genetics, University of Utah School of Medicine, Salt Lake City, UT 84112, USA.; ^7^ Vaccine and Infectious Disease Organization, University of Saskatchewan, Saskatoon SK S7N 5E3, Canada.; ^8^ Center for Global Health Science and Security, Georgetown University, Washington, DC 20057, USA.; ^9^ Department of Zoology, University of Oxford, Oxford OX1 3SZ, UK.; ^10^ Wildlife Conservation Research Unit, Department of Zoology, The Recanati-Kaplan Centre, University of Oxford, Oxford OX13 5QL, UK.; ^11^ Pandemic and Disaster Preparedness Centre, Erasmus University Medical Center, 3015 CE Rotterdam, Netherlands.; ^12^ Department of Viroscience, Erasmus University Medical Center, 3015 CE Rotterdam, Netherlands.; ^13^ Department of Biostatistics, Fielding School of Public Health, University of California Los Angeles, Los Angeles, CA 90095, USA.; ^14^ Department of Human Genetics, David Geffen School of Medicine, University of California Los Angeles, Los Angeles, CA 90095, USA.; ^15^ Department of Computational Medicine, David Geffen School of Medicine, University of California Los Angeles, Los Angeles, CA 90095, USA.; ^16^ Department of Medicine, University of California San Diego, La Jolla, CA 92093, USA.; ^17^ Department of Microbiology, Immunology and Transplantation, Rega Institute for Medical Research, KU Leuven, 3000 Leuven, Belgium.; ^18^ Global Virus Network (GVN), Baltimore, MD 21201, USA.; ^19^ MRC-University of Glasgow Center for Virus Research, Glasgow G61 1QH, UK.; ^20^ Tulane University, School of Medicine, Department of Microbiology and Immunology, New Orleans, LA 70112, USA.; ^21^ Zalgen Labs, Frederick, MD 21703, USA.; ^22^ Sydney Institute for Infectious Diseases, School of Life and Environmental Sciences and School of Medical Sciences, The University of Sydney, Sydney, New South Wales 2006, Australia.; ^23^ Institute of Evolutionary Biology, University of Edinburgh, Edinburgh EH9 3FL, UK.; ^24^ Scripps Research Translational Institute, La Jolla, CA 92037, USA.

## Abstract

Understanding how severe acute respiratory syndrome coronavirus 2 (SARS-CoV-2) emerged in 2019 is critical to preventing zoonotic outbreaks before they become the next pandemic. The Huanan Seafood Wholesale Market in Wuhan, China, was identified as a likely source of cases in early reports but later this conclusion became controversial. We show the earliest known COVID-19 cases from December 2019, including those without reported direct links, were geographically centered on this market. We report that live SARS-CoV-2 susceptible mammals were sold at the market in late 2019 and, within the market, SARS-CoV-2-positive environmental samples were spatially associated with vendors selling live mammals. While there is insufficient evidence to define upstream events, and exact circumstances remain obscure, our analyses indicate that the emergence of SARS-CoV-2 occurred via the live wildlife trade in China, and show that the Huanan market was the epicenter of the COVID-19 pandemic.

On 31 December 2019, the Chinese government notified the World Health Organization (WHO) of an outbreak of severe pneumonia of unknown etiology in Wuhan, Hubei province (*1*–*4*), a city of approximately 11 million people. Of the initial 41 people hospitalized with unknown pneumonia by 2 January 2020, 27 (66%) had direct exposure to the Huanan Wholesale Seafood Market (hereafter, “Huanan market”) (*2*, *5*, *6*). These first cases were confirmed to be infected with a novel coronavirus, subsequently named severe acute respiratory syndrome coronavirus 2 (SARS-CoV-2) and were suffering from a disease later named coronavirus disease 2019 (COVID-19). The initial diagnoses of COVID-19 were made in several hospitals independently between 18 and 29 December 2019 (*5*). These early reports were free from ascertainment bias as they were based on signs and symptoms before the Huanan market was identified as a shared risk factor (*5*). A subsequent systematic review of all cases notified to China’s National Notifiable Disease Reporting System by hospitals in Wuhan as part of the joint WHO-Chinese “WHO-convened global study of origins of SARS-CoV-2: China Part” (hereafter, “WHO mission report”) (*7*) showed that 55 of 168 of the earliest known COVID-19 cases were associated with this market. However, the observation that the preponderance of early cases were linked to the Huanan market does not establish that the pandemic originated there.

Sustained live mammal sales during 2019 occurred at the Huanan and three other markets in Wuhan, including wild and farmed wild-life (*8*). Several of these species are known to be experimentally susceptible to SARS-related coronaviruses (SARSr-CoVs), such as SARS-CoV (hereafter, “SARS-CoV-1”) and SARS-CoV-2 (*9*–*11*). During the early stages of the COVID-19 pandemic, animals sold at the Huanan market were hypothesized to be the source of the unexplained pneumonia cases (*12*–*19*) **(**data S1), consistent with the emergence of SARS-CoV-1 from 2002-2004 (*20*), as well as other viral zoonoses (*21*–*23*). This led to the decision to close and sanitize the Huanan market on 1 January 2020, with environmental samples also being collected from vendors’ stalls (*7*, *12*, *24*) (data S1).

Determining the epicenter of the COVID-19 pandemic at a neighborhood- rather than city-level could help resolve if SARS-CoV-2 had a zoonotic origin, similar to SARS-CoV-1 (*20*). In this study, we obtained data from a range of sources to test the hypothesis that the COVID-19 pandemic began at the Huanan market. Despite limited testing of live wildlife sold at the market, collectively, our results provide evidence that the Huanan market was the early epicenter of the COVID-19 pandemic and suggest that SARS-CoV-2 likely emerged from the live wildlife trade in China. However, events upstream of the market, as well as exact circumstances at the market, remain obscure, highlighting the need for further studies to understand and lower the risk of future pandemics.

## Results

### Early cases lived near to and centered on the Huanan market

The 2021 WHO mission report identified 174 COVID-19 cases in Hubei province in December 2019 after careful examination of reported case histories (*7*). Although geographical coordinates of the residential locations of the 164 cases who lived within Wuhan were unavailable, we were able to reliably extract the latitude and longitude coordinates of 155 cases from maps in the report (figs. S1 to S8).

**Fig. 1.**
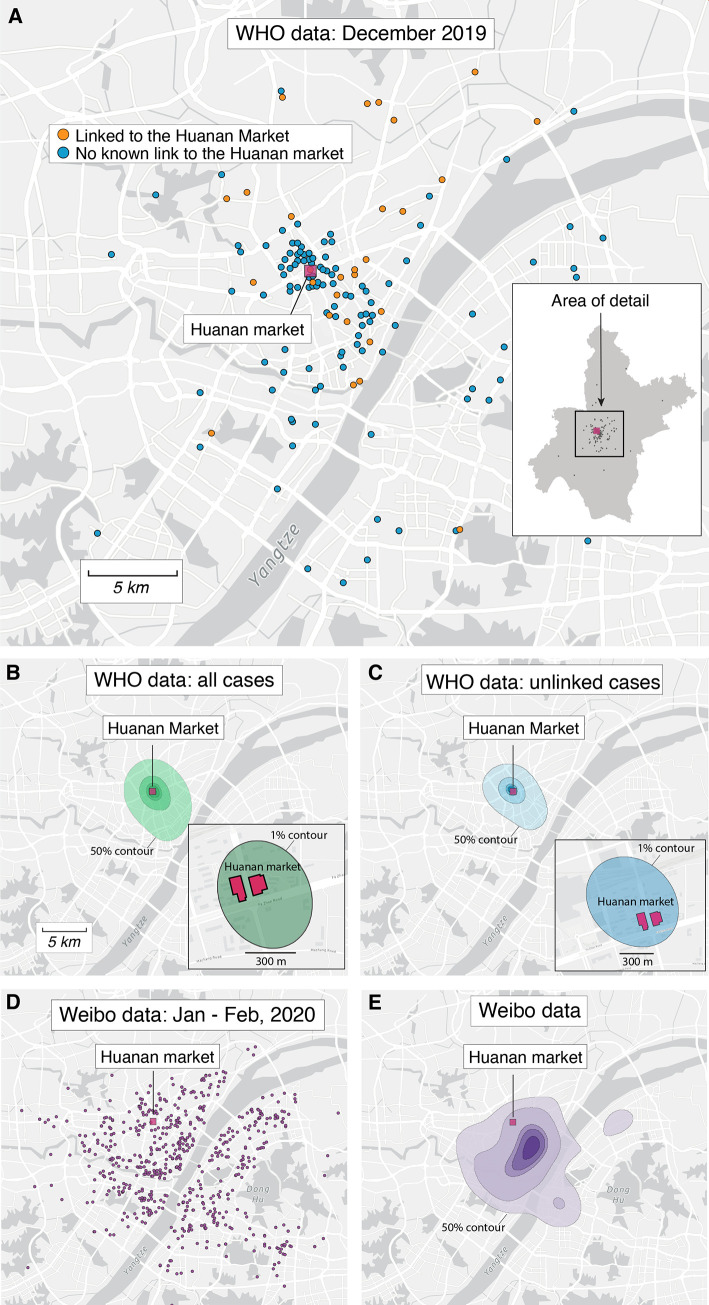
Spatial patterns of COVID-19 cases in Wuhan in December 2019 and January-February 2020. (**A**) Locations of the 155 cases we extracted from the WHO mission report (*7*). Inset: map of Wuhan with December 2019 case indicated with gray dots. (No cases are obscured by the inset.) In both the inset and the main panel the location of the Huanan market is indicated with a red square. (**B**) Probability density contours reconstructed by a kernel density estimate (KDE) using all 155 COVID-19 cases locations from December 2019. The highest density 50% contour marked is the area for which cases drawn from the probability distribution are as likely to lie inside as outside. Also shown are the highest density 25%, 10%, 5%, and 1% contours. Inset showing an expanded view and the highest density 1% probability density contour. (**C**) Probability density contours reconstructed using the 120 COVID-19 cases locations from December 2019 that were unlinked to the Huanan market. (**D**) Locations of 737 COVID-19 cases from Weibo data dating to January and February of 2020. (**E**) The same highest probability density contours (50% through 1%) for 737 COVID-19 case locations from Weibo data.

While early COVID-19 cases occurred across Wuhan, the majority clustered in central Wuhan near the west bank of the Yangtze River, with a high density of cases near to, and surrounding, the Huanan market (Fig. 1A). We used a kernel density estimate (KDE) to reconstruct an underlying probability density function from which the home locations for each case were drawn (*25*). Using all 155 December 2019 cases, the location of the Huanan market lies within the highest density contour that contains 1% of the probability mass (Fig. 1B). For a KDE estimated using the 120 cases with no known linkage to the market, the market remains within the highest density 1% contour (Fig. 1C). The clustering of COVID-19 cases in December around the Huanan market (Fig. 1, B and C, insets) contrasts with the pattern of widely dispersed cases across Wuhan by early January through mid-February 2020 (Fig. 1, D and E), which we mapped using location data from individuals using a COVID-19 assistance app on Sina Weibo (*26*). Weibo-based data analyses show that, unlike early COVID-19 cases, by January and February many of the sick who sought help resided in highly populated areas of the city, and particularly in areas with a high density of older people **(** Fig. 1E and figs. S9 and S10).

**Fig. 2.**
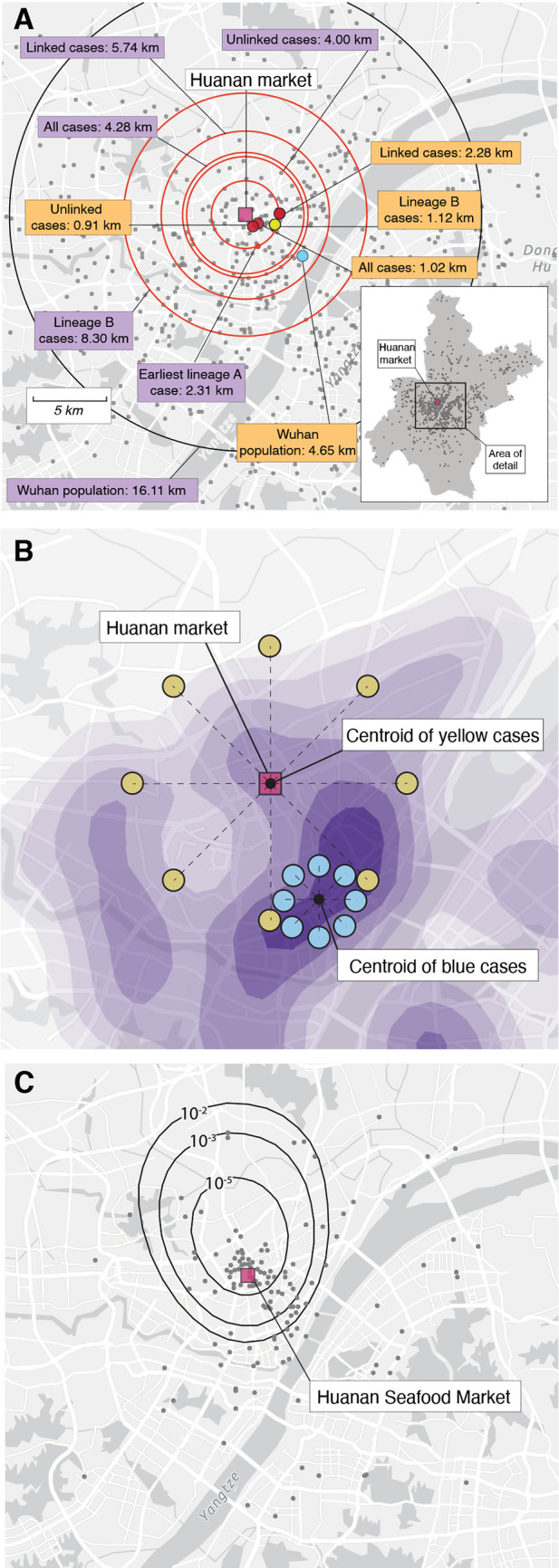
Spatial analyses. (**A**) Inset: map of Wuhan, with gray dots indicating 1000 random samples from worldpop.com null distribution. Main panel: median distance between Huanan market and (1) worldpop.org null distribution shown with a black circle and (2) December cases shown by red circles (distance to Huanan market depicted in purple boxes). Center-point of Wuhan population density data shown by blue dot. Center-points of December case locations shown by red dots (‘all’, ‘linked’ and ‘unlinked’ cases); dark blue dot (lineage A cases); and yellow dot (lineage B cases). Distance from center-points to Huanan market depicted in orange boxes. (**B**) Schematic showing how cases can be near to, but not centered on, a specific location. We hypothesized that if the Huanan market epicenter of the pandemic then early cases should fall not just unexpectedly near to it but should also be unexpectedly centered on it (see Methods). The blue cases show how cases quite near the Huanan market could nevertheless not be centered on it. (**C**) Tolerance contours based on relative risk of COVID-19 cases in December, 2019 versus data from January-February 2020. The dots show the December case locations. The contours represent the probability of observing that density of December cases within the bounds of the given contour if the December cases had been drawn from the same spatial distribution as the January-February data.

We also investigated whether the December COVID-19 cases were closer to the market than expected based on an empirical null distribution of Wuhan’s population density (data from worldpop.org (*27*, *28*)), with its median distance to the Huanan market of 16.11km (*25*). To account for older individuals being more likely to be hospitalized and sick with COVID-19 (*29*), we age-matched the population data to the December 2019 COVID-19 case data. We considered three categories of cases, and they were all significantly closer to the Huanan market than expected: (*i*) all cases (median 4.28km: *p*<0.001), (*ii*) cases linked directly to the Huanan market (median 5.74km; *p*<0.001), and (*iii*) cases with no evidence of a direct link to the Huanan market (median 4.00km: *p*<0.001) (Fig. 2A). The cases with no known link to the market on average resided closer to the market than the cases with links to the market (*p*=0.029). Furthermore, the distances between the center-points (Fig. 2B) and the Huanan market were shorter than expected for all categories of December cases compared with the empirical null distribution of Wuhan’s population density (Fig. 2A). For all the December cases the center-point was located 1.02km away (*p*=0.007); the center-point for cases with market links was 2.28km away (*p*=0.034), and the center-point for the cases with no reported link to the market was 0.91km away (*p*=0.006). In comparison, the center-point of age-matched samples drawn from the empirical null distribution was 4.65km away from the market (Fig. 2A).

We tested the robustness of our results to the possibility of ascertainment bias (*25*). For all mapped cases (*n*=155), under the ‘center-point distance to the Huanan market’ test, the 38 cases residing closest to the market (within a radius of 1.6km) could be removed from the data set before losing significance at the α*=0.05* level (fig. S12). For the ‘median distance to Huanan market’ test, we could remove 98 (63%) (*r=*5.8km). For cases not directly linked to the Huanan market (n=120), we could remove 36 (30%) (*r=*1.5km) and 81 (68%) (*r=*4.3km) for the two tests, respectively, before losing significance at the α*=0.05* level (fig. S12).

We performed a spatial relative risk analysis (*25*) to compare December 2019 COVID-19 cases with January-February 2020 cases, reported via Weibo (Fig. 2C). The Huanan market is located within a well-defined area with high case density that would be expected to be observed in fewer than one in 100,000 samplings of the Weibo data empirical distribution (relative risk analysis in Fig. 2C, control distribution in Fig. 1D). No other regions in Wuhan showed a comparable case density.

### Both early lineages of SARS-CoV-2 were geographically associated with the market

Two lineages of SARS-CoV-2 designated A and B (*30*) have co-circulated globally since early in the COVID-19 pandemic (*31*). Until a report in a recent preprint (*24*), only lineage B sequences had been sampled at the Huanan market. The eleven lineage B cases from December 2019, for which we have location information, resided closer than expected to the Huanan market compared to the age-matched Wuhan population distribution (median 8.30km; *p*=0.017) (*25*). The center-point of the eleven lineage B cases was 1.95km from the Huanan market, also closer than expected (*p*=0.026). The two lineage A cases for which we have location information involved the two earliest lineage A genomes known to date. Neither case reported any contact to the Huanan market (*7*). The first case was detected before any knowledge of a possible association of unexplained pneumonia in Wuhan with the Huanan market (*5*) and therefore could not have been a product of ascertainment bias in favor of cases residing near the market. The second had stayed in a hotel near the market (*32*) for the five days preceding symptom onset (*25*). Relative to the age-matched Wuhan population distribution, the first individual resided closer to the Huanan market (2.31km) than expected (*p*=0.034). While the exact location of the hotel near the market was not reported (*32*), there are at least 20 hotels within 500 m (table S1). Under the conservative assumption that the hotel could have been located as far as 2.31km from the Huanan market (as was the residence of the other lineage A case), and assuming this location is comparable to a residential location given the timing of the stay prior to symptom onset (*25*), it would be unlikely to observe both the earliest lineage A cases this near to the Huanan market (*p*=0.001 or less). That both identified lineage A cases had a geographical connection to the market, in combination with the detection of lineage A within the market (*24*), support the likelihood that during the early epidemic lineage A was, like lineage B, disseminating outward from the Huanan market into the surrounding neighborhoods.

Our statistical results were robust to a range of factors, for example, the use of an empirical control distribution based on presumptive COVID-19 cases locations later in the Wuhan epidemic (Weibo data); laboratory-confirmed versus clinically-diagnosed cases; and uncertainty in case location or missing data (figs. S13 to S15) (*25*). For instance, we artificially introduced location uncertainty (‘noise’) in each case location in our data set by randomly re-sampling each point within a circle of radius 1000m centered on its original center-point; the conclusions were unaffected (fig. S13). The extraction method we employed actually introduced up to about 50m of noise in each case location estimate (fig. S7), ruling out the possibility that our overall results were affected by this source of error. The results were also robust when corrected for multiple hypothesis testing (table S4).

### Wild animal trading in Wuhan markets

In addition to selling seafood, poultry, and other commodities, the Huanan market was among four markets in Wuhan reported to consistently sell a variety of live, wild-captured or farmed, mammal species in the years and months leading up to the COVID-19 pandemic (*8*). There are, however, no prior reports of which species, if any, were sold at the Huanan market in the months leading up to the pandemic. Here, we report that multiple plausible intermediate wildlife hosts of SARS-CoV-2 progenitor viruses, including red foxes (*Vulpes vulpes*), hog badgers (*Arctonyx albogularis*) and common raccoon dogs (*Nyctereutes procyonoides*), were sold live at the Huanan market up until at least November of 2019 **(** Table 1 and table S5**)**. No reports are known to be available for SARS-CoV-2 test results from these mammals at the Huanan market. Despite a general slow-down in live animal sales during the winter months, we report that raccoon dogs that are sold for both meat and fur were consistently available for sale throughout the year, including at the Huanan market in November 2019 (Table 1 and table S5).

**Table 1.** Live mammals traded at the Huanan market in November and December 2019

Species (susceptibility*)	**Family** (susceptibility*)	Order (susceptibility*)	**Observed at Huanan market, November 2019**
Raccoon dog (*Nyctereutes procyonoides*) (Y)	Canidae (Y)	Carnivora (Y)	Y
Amur hedgehog (*Erinaceus amurensis*)	Erinaceidae	Eulipotyphla	Y
Hog badger (*Arctonyx albogularis*) (Y)	Mustelidae (Y)	Carnivora (Y)	Y
Asian badger (*Meles leucurus*)	Mustelidae (Y)	Carnivora (Y)	Y
Chinese hare (*Lepus sinensis*)	Leporidae (Y)	Lagomorpha (Y)	Y
Chinese bamboo rat (*Rhizomys sinensis*) (Y)	Spalacidae (Y)	Rodentia (Y)	Y
Malayan porcupine (*Hystrix brachyura*)	Hystricidae	Rodentia (Y)	Y
Chinese muntjac (*Muntiacus reevesi*)	Cervidae (Y)	Artiodactyla (Y)	Y
Marmot (*Marmota himalayana*)	Sciuridae	Rodentia (Y)	Y
Red fox (*Vulpes vulpes*) (Y)	Canidae (Y)	Carnivora (Y)	Y
Siberian weasel (*Mustela sibirica*)	Mustelidae (Y)	Carnivora (Y)	N†
Pallas's squirrel (*Callosciurus erythraeus*)	Sciuridae	Rodentia (Y)	N
Masked palm civet (*Paguma larvata*) (Y)	Viverridae (Y)	Carnivora (Y)	N
Coypu (*Myocastor coypus*)	Echimyidae	Rodentia (Y)	N
Mink (*Neovison vison*) (Y)	Mustelidae (Y)	Carnivora (Y)	N
Red squirrel (*Sciurus vulgaris*)	Sciuridae	Rodentia (Y)	N
Wild boar (*Sus scrofa*) (Y)	Suidae (Y)	Artiodactyla (Y)	N
Complex-toothed flying squirrel (*Trogopterus xanthipes*)	Sciuridae	Rodentia (Y)	N

*Based on live susceptibility findings, serological findings, or ACE2-binding assays. See table S5 for details and associated references.

†Animals listed as “No” were, however, present at Wuhan markets during the 2017–2019 study period (*8*).

**Fig. 3.**
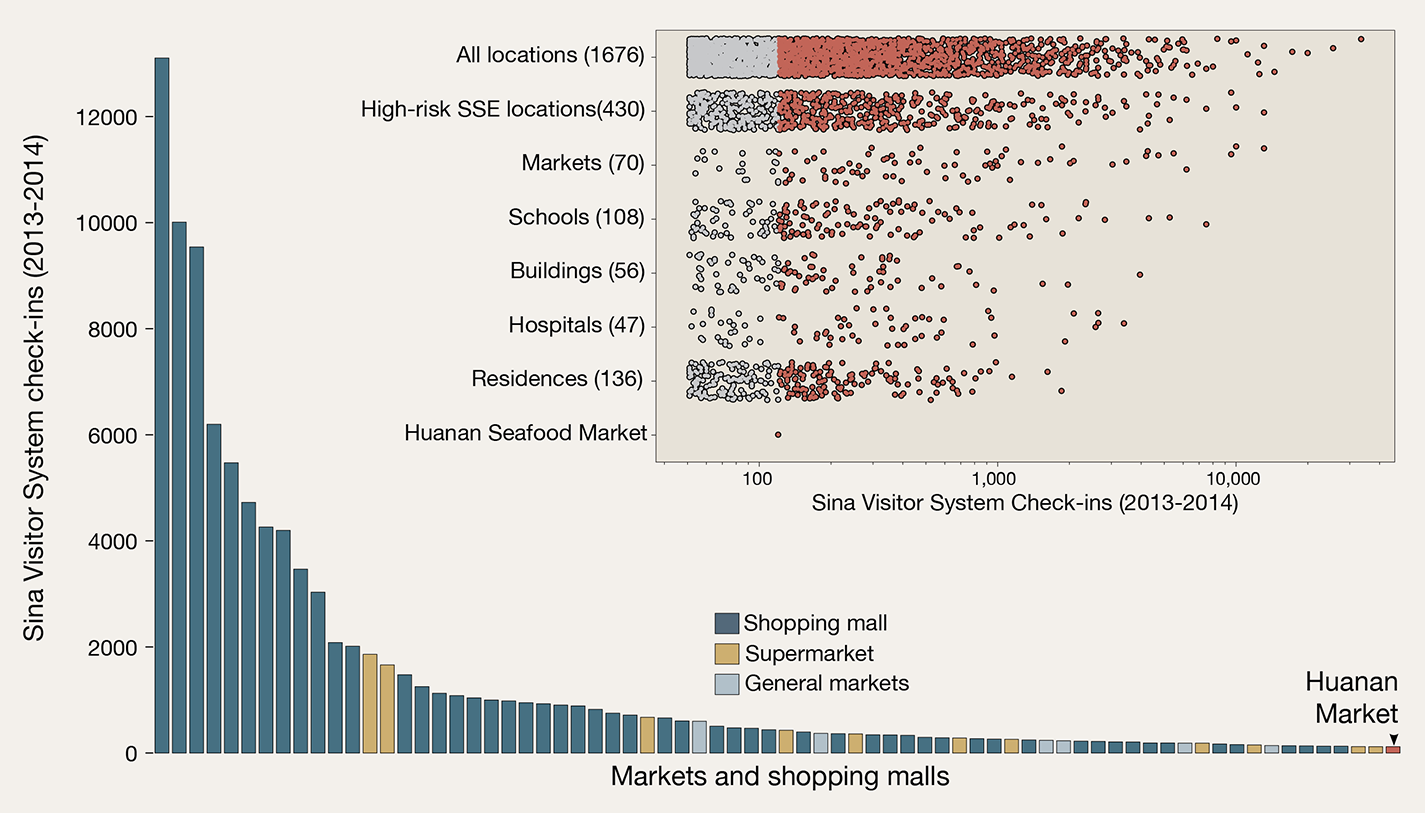
Visitors to locations throughout Wuhan. Number of social media check-ins in the Sina Visitor System from 2013-2014 as shared by (*33*). Number of visitors to individual markets throughout the city are shown in comparison to the Huanan market. Inset: the total number of check-ins to all individual locations across the city of Wuhan, grouped by category. Locations with more than 50 visitor check-ins are shown, and the locations which received more check-ins than the Huanan market in the same period are shown in red.

There were potentially many locations in Wuhan, a city of 11 million, that would have been equally or more likely than the Huanan market to sustain the first recognized cluster of a new respiratory pathogen had its introduction not been linked to a live animal market, including other shopping venues, hospitals, elder care facilities, workplaces, universities, and places of worship. To investigate possible sites, we compared the relative extent of intra-urban human traffic to the Huanan market versus other locations within the city of Wuhan using a location-specific data set of social media check-ins in the Sina Visitor System (*25*, *33*). We found at least 70 other markets throughout the city of Wuhan that received more social media visitors than the Huanan market (Fig. 3). To extend this analysis beyond only markets, we also used a subsequently published list of known SARS-CoV-2 superspreader locations (*34*) to identify 430 locations in Wuhan that may have been at high risk for superspreader events and which received more check-ins than the Huanan market (Fig. 3, inset). The Huanan market accounted for 0.12% (120 of 98,146) of social media check-ins to markets in the data set that received at least as many check-ins as the Huanan market. The market accounted for 0.04% (120 of 262,233) of all social media check-ins to the >400 sites in Wuhan identified as especially likely to be potential superspreader locations and which received at least as many social media visits as the Huanan market. Considering the number of check-ins to all four markets selling live, wild animals in Wuhan (combined), they accounted for 0.21% (206 of 98,146) of market visits and 0.079% (206 of 262,233) of visits to the 430 potential superspreader sites, where a new respiratory disease might first be noticed in a large city.

**Fig. 4.**
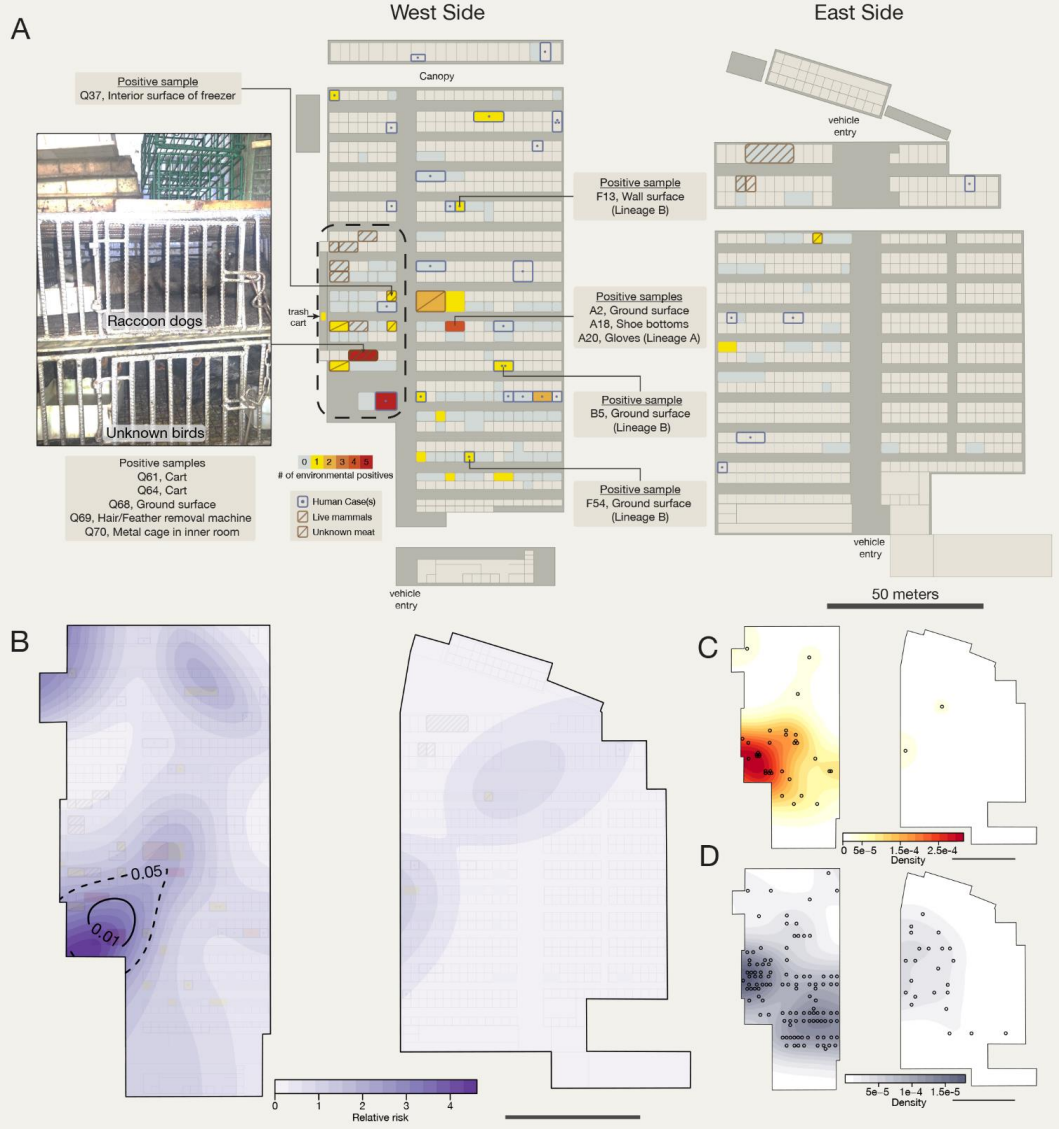
Map of the Huanan Wholesale Seafood Market. (**A**) Aggregated environmental sampling and human case data from Huanan Market. Captions (left) describe the types of SARS-CoV-2 positive environmental samples obtained from known live animal vendors and (center) from stalls with samples with known virus lineage. Lineage is unknown unless noted; sequencing data has not been released for some samples and many samples were PCR-positive but not sequenced. Image (left) of raccoon dogs in a metal cage, on top of caged birds, taken in business with five positive environmental samples (photo credit: E.C.H.). Rectangle with dashed outline is used to denote the ‘wildlife’ section of the market. (**B**) Relative risk analysis of positive environmental samples. Tolerance contours enclose regions with statistically significant elevation in density of positive environmental samples relative to the distribution of sampled stalls. (**C**) Distribution of positive environmental samples. Sample locations (centroid of corresponding business) and quantity are shown as black circles. (**D**) Control distribution for relative risk analysis. All businesses investigated with environmental sampling are shown as black circles (one per business, whether or not a positive sample was found). See table S12 for details on stalls that were SARS-CoV-2-negative.

A data set from the Chinese Center for Disease Prevention and Control (CCDC) report dated 22 January 2020 **(**data S1**)** (*12*, *13*, *15*, *16*) was made publicly available in June 2020 (*24*, *35*). 585 environmental samples were initially taken from various surfaces in the Huanan market on 1 and 12 January 2020 by the CCDC **(**tables S6 and S7 and data S1) (*12*, *13*, *15*, *16*, *24*, *35*), with further samples taken through the market during January and February (*24*). We extended the analysis in the WHO mission report (*7*) by integrating public online maps and photographic evidence, data from public business registries **(**table S8 and data S2**)**, information about which live mammal species were sold at the Huanan market in late 2019 (Table 1 and table S5**)**, and the CCDC report **(**data S1). We reconstructed the floor plan of the market and integrated information from business registries of vendors at the market (fig. S16 and table S8), as well as an official report (*36*) recording fines to three business owners for illegal sale of live mammals (data S2**)** (*36*). From this, we identified an additional five stalls that were likely selling live or freshly butchered mammals or other unspecified meat products in the southwest corner of the western section of the market (Fig. 4A, figs. S16 and S17, and table S6).

Five of the SARS-CoV-2-positive environmental samples were taken from a single stall selling live mammals in late 2019 (table S6). Further, the objects sampled showed an association with animal sales, including a metal cage, two carts (of the kind frequently used to transport mobile animal cages) and a hair/feather remover (table S6). No human COVID-19 cases were reported there (*7*, *12*). The same stall was visited by one of us (ECH) in 2014, who then observed live raccoon dogs housed in a metal cage stacked on top of a cage with live birds (Fig. 4A) (*37*). A recent report (*24*) identified that the grates outside of this stall, upon which animal cages were stacked (*37*), were positive for SARS-CoV-2.

### Positive environmental samples linked both to live mammal sales and to human cases at the Huanan market

We used a spatial relative risk analysis to identify potential regions of the market with an increased density of positive environmental samples (*25*). We found evidence (*p*<0.05) of a region in the southwest area of the market where live mammals were on sale (Fig. 4B). Although environmental sampling of the market was incomplete and spatially heterogeneous **(**data S1 and table S6**)**, our analysis accounts for the empirical environmental sampling distribution, which was biased toward ‘stalls related to December cases’ as well as ‘stalls that sold livestock, poultry, farmed wildlife’ (*7*) **(** Fig. 4, C and D **)**. The ‘distance to the nearest vendor selling live mammals’ and ‘distance to the nearest human case’ were independently predictive of environmental sample positivity (*p*=0.004 and 0.014, respectively for N=6; table S9). To further investigate the robustness of these findings to possible sampling biases, we considered three scenarios: (*i*) oversampling of live mammal and unknown meat stalls, (*ii*) over-counting of positive samples, and (*iii*) exclusion of the seafood stand near the wildlife area of the market (with five positive samples) from our analysis (table S10). In each case, the distance to live mammal vendors remained predictive of environmental sample positivity, and the region of increased positive sample density in the southwest corner of the western section of the market remained consistent (fig. S18).

**Fig. 5.**
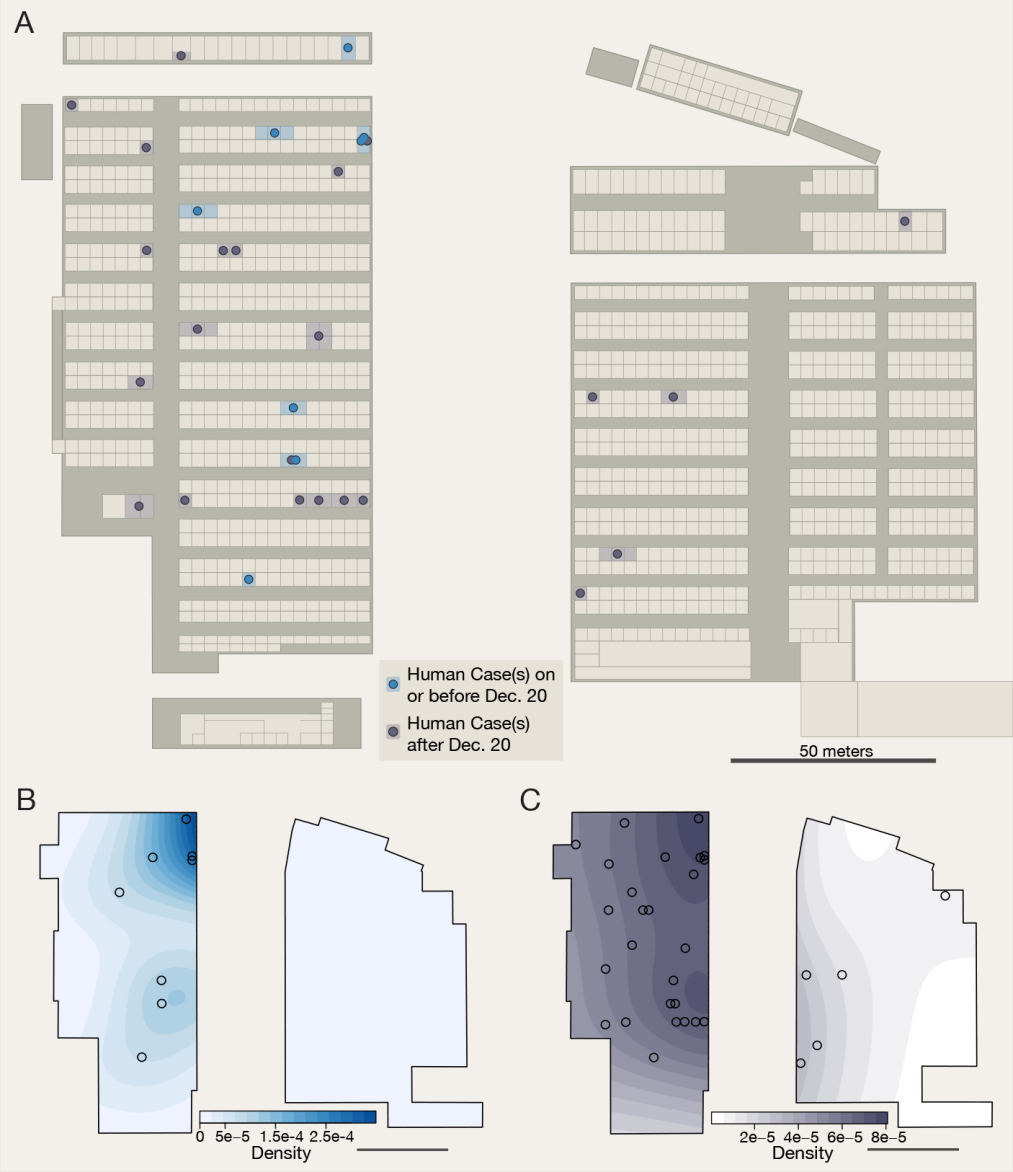
Location and timing of human cases in Huanan market. (**A**) Outline colors correspond to the timing of the first known case in each business. Individual case timing is denoted by marker color and shown within the outlined business. (**B**) Distribution of known cases on or before December 20th, 2019. Locations of each case are shown as a black circle. (**C**) Distribution of all known human cases in Huanan Market. See table S11 for details on SARS-CoV-2 positive human cases with the Huanan market.

Finally, to analyze the spatial patterning of human cases within the Huanan market, we plotted cases as a function of symptom onset from the WHO mission report (*7*) (Fig. 5A and table S11) (*25*). All eight COVID-19 cases detected prior to 20 December were from the western side of the market, where mammal species were also sold (Fig. 5, B and C). Unlike SARS-CoV-2 positive environmental samples (Fig. 4, A and C), we found that COVID-19 cases were more diffuse throughout the building (Fig. 5).

### Study limitations

There are several limitations to our study. We have been able to recover location data for most of the December-onset COVID-19 cases identified by the WHO mission (*7*) and have been able to do so with sufficient precision to support our conclusions. However, we do not have access to the precise latitude and longitude coordinates of all these cases. Should such data exist, they may be accompanied by additional metadata, some of which we have reconstructed, but some of which, including the date of onset of each case, would be valuable for ongoing studies. We also lack direct evidence of an intermediate animal infected with a SARS-CoV-2 progenitor virus either at the Huanan market or at a location connected to its supply chain, like a farm. Additionally, no line list of early COVID-19 cases is available and we do not have complete details of environmental sampling, though compared to many other outbreaks, we have more comprehensive information on early cases, hospitalizations and environmental sampling (*7*).

## Discussion

Several lines of evidence support the hypothesis that the Huanan market was the epicenter of the COVID-19 pandemic and that SARS-CoV-2 emerged from activities associated with live wildlife trade. Spatial analyses within the market show that SARS-CoV-2-positive environmental samples, including cages, carts, and freezers, were associated with activities concentrated in the southwest corner of the market. This is the same section where vendors were selling live mammals, including raccoon dogs, hog badgers, and red foxes, immediately prior to the COVID-19 pandemic. Multiple positive samples were taken from one stall known to have sold live mammals, and the water drain proximal to this stall, as well as other sewerages and a nearby wildlife stall on the southwest side of the market, tested positive for SARS-CoV-2 (*24*). These findings suggest that infected animals were present at the Huanan market at the beginning of the COVID-19 pandemic; however, we do not have access to any live animal samples from relevant species. Additional information, including sequencing data and detailed sampling strategy, would be invaluable to test this hypothesis comprehensively.

In a related study, we infer separate introductions of SARS-CoV-2 lineages A and B into humans from likely infected animals at the Huanan market (*38*). We estimate the first COVID-19 case to have occurred in November 2019, with few human cases and hospitalizations occurring through mid-December (*38*). A recent preprint (*24*) confirms the authenticity of the CCDC report **(**data S1**)** and records additional positive environmental samples in the southwestern area of the market selling live animals. This report also documents the early presence of the A lineage of SARS-CoV-2 in a Huanan market environmental sample. This, along with the lineage A cases we report in close geographical proximity to the market in December, challenges the suggestion that the market was simply a superspreading event, which would be lineage-specific. Rather, it adds to the evidence presented here that lineage A, like lineage B, may have originated at the Huanan market then spread from this epicenter into the neighborhoods surrounding the market and then beyond.

Several observations suggest that the geographic association of early COVID-19 cases with the Huanan market is unlikely to have been the result of ascertainment bias (supplementary text and tables S2 and S3) (*39*). These include: (*i*) few, if any, cases among Huanan market-unlinked individuals are likely to have been detected by active searching in the neighborhoods around the market – only in hospitals – since all cases analyzed here were hospitalized (*7*), (*ii*) public health officials simultaneously became aware of Huanan-linked cases near and far from the Huanan market, not just ones near it (fig. S11) (*5*), (*iii*) Huanan-unlinked cases would not be expected to live significantly closer to the market than linked cases if they had been ascertained as contacts traced from those market-linked cases, and (*iv*) seroprevalence in Wuhan was highest in the districts around the market (*40*, *41*). It is also noteworthy that the December 2019 COVID-19 cases we consider here were identified based on reviews of clinical signs and symptoms, not epidemiological factors such as where they resided or links to the Huanan market (*7*) and that excess deaths from pneumonia rose first in the districts surrounding the market (*42*). Moreover, the spatial relationship with the Huanan market remains after removing the two-thirds of the unlinked cases residing nearest the market.

One of the key findings of our study is that ‘unlinked’ early COVID-19 patients, those who neither worked at the market or knew someone who did, nor had recently visited the market, resided significantly closer to the market than patients with a direct link to the market. The observation that a substantial proportion of early cases had no known epidemiological link had previously been used as an argument against a Huanan market epicenter of the pandemic. However, this group of cases resided significantly closer to the market than those who worked there, indicating that they had been exposed to the virus at, or near, the Huanan market. For market workers, the exposure risk was their place of work not their residential locations, which were significantly further afield than those cases not formally linked to the market.

Our spatial analyses show how patterns of COVID-19 cases shifted between late 2019, when the outbreak began (*43*), and early 2020, as the epidemic spread widely across Wuhan. COVID-19 cases in December 2019 were associated with the Huanan market in a manner unrelated to Wuhan population density or demographic patterns, unlike the wide spatial distribution of cases observed during later stages of the epidemic in January and February. This observation fits with the evidence from other sources that SARS-CoV-2 was not widespread in Wuhan at the end of 2019. For example, no SARS-CoV-2-positive sera or influenza-like illness (ILI) reports were recorded among more 40,000 blood donor samples collected up to December 2019 (*44*, *45*), and none of thousands of samples taken from ILI patients at Wuhan hospitals in October-December 2019 tested for SARS-CoV-2 RNA was positive (*7*).

The sustained presence of a potential source of virus transmission into the human population in late 2019, plausibly from infected live mammals sold at the Huanan market, offers an explanation of our findings and the origins of SARS-CoV-2. The pattern of COVID-19 cases reported for the Huanan market, with the earliest cases in the same part of the market as the wildlife sales and evidence of at least two introductions (*38*), resembles the multiple cross-species transmissions of SARS-CoV-2 subsequently observed during the pandemic from animals to humans on mink farms (*46*), and from infected hamsters to humans in the pet trade (*47*). There was an extensive network of wildlife farms in western Hubei province, including hundreds of thousands of raccoon dogs on farms in Enshi prefecture, which supplied the Huanan market (*48*). This region of Hubei contains extensive cave complexes housing *Rhinolophus* bats, which carry SARSr-CoVs (*49*). SARS-CoV-1 was recovered from farmed masked palm civets from Hubei in 2003 and 2004 (*20*). The animals on these farms (nearly 1 million) were rapidly released, sold, or killed in early 2020 (*48*), apparently without testing for SARS-CoV-2 (*7*). Live animals sold at the market (Table 1) were apparently not sampled either. By contrast, during the SARS-CoV-1 outbreaks farms and markets remained open for over a year after the first human cases occurred, allowing sampling of viruses from infected animals (*20*).

The live animal trade and live animal markets are a common theme in virus spillover events (*21*–*23*, *50*), with markets such as the Huanan market selling live mammals being in the highest risk category (*51*). The events leading up to the COVID-19 pandemic mirror the SARS-CoV-1 outbreaks from 2002-2004, which were traced to infected animals in Guangdong, Jiangxi, Henan, Hunan, and Hubei provinces in China (*20*). Maximum effort must now be applied to elucidate the upstream events that might have brought SARS-CoV-2 into the Huanan market, culminating in the COVID-19 pandemic. To reduce the risk of future pandemics we must understand, and then limit, the routes and opportunities for virus spillover.

## Methods summary

### Ethics statement

This research was reviewed by the Human Subject Protection Program at the University of Arizona and the Institutional Review Board at The Scripps Research Institute and determined to be exempt from IRB approval because it constitutes secondary research for which consent is not required.

### Data sources

COVID-19 case data from December 2019 was obtained from the WHO mission report (*7*) and our previous analyses (*5*). Location information was extracted and sensitivity analyses performed to confirm accuracy and assess potential ascertainment bias. Geotagged January/February 2020 data from Weibo COVID-19 help seekers was obtained from the authors (*26*). Population density data was obtained from worldpop.org (*27*). Sequencing- or qPCR-based environmental sample SARS-CoV-2 positivity from the Huanan market was obtained from a January 2020 China CDC report (data S1) (*24*).

### Wildlife trading at the Huanan market

Animal sales from Wuhan wet markets immediately prior to the COVID-19 pandemic was previously reported (*8*) and in this study we report details about animals for sale at the Huanan market up until November 2019.

### Spatial analyses of COVID-19 cases

Haversine distances to the Huanan market were calculated for each of the geolocated December 2019 cases. Center-points and median distances from cases to the Huanan market were calculated separately for (1) all 155 cases, (2) the 35 cases epidemiologically linked to the Huanan market, (3) for the 120 cases not epidemiologically linked to the market, (4) the eleven lineage B cases, and (5) the earliest lineage A case. These distances were also calculated for the 737 Weibo help seekers from 8 January to 10 February 2020 (*26*). Empirical null distributions were generated from the population density data and the Weibo data. The population density null distributions were age-matched to the December 2019 cases.. Kernel density estimates were also generated for the market-linked cases, unlinked-cases and all cases, to infer a probability density function from which the cases could have been drawn. Highest-density contours representing specific probability masses (0.5, 0.25, 0.1, 0.05, and 0.01) were inferred and the location of the market compared to these.

### Mobility analyses

To estimate the relative amount of intra-urban human traffic to the Huanan market compared to other locations within the city of Wuhan, we utilized a location-specific dataset of social media check-ins in the Sina Visitor System as shared by Li *et al*. 2015 (*33*). This dataset is based on 1,491,499 individual check-in events across the city of Wuhan from the years 2013-2014 (5-6 years before the start of the COVID-19 pandemic), and 770,521 visits are associated with 312,190 unique user identifiers. Location names and categories were translated using a Python API for Google Translate.

### Spatial analyses of environmental samples at the Huanan market

We used the official maps from the China CDC (*12*) (data S1) and WHO map (*7*), as well as satellite photographs (Google Maps, Google Earth, Baidu Maps), aerial photographs, and images of the market in the public domain to reconstruct the floorplan of the market. Market stalls were assigned by categories of the types of goods sold using official reports and data from the TianYanCha.com business directory (table S8). Final maps of the Huanan market were converted into geojson format for spatial analyses. Significance testing of live animal vendors and/or human SARS-CoV-2 cases on the number of positive environmental samples was performed using a binomial GLM. Distances between businesses were defined as the distance between their respective center-points and spatial relative risk analysis was performed using the ‘sparr’ package in R, using linear boundary kernels for edge correction (*52*), with bandwidth selection performed using least squares cross-validation.

## Supplementary Materials

### This PDF file includes:

Materials and Methods Supplementary Text Figs. S1 to S18 Tables S1 to S12 References (*54*–*81*) Data S1 and S2

### Other Supplementary Material for this manuscript includes the following:

MDAR Reproducibility Checklist
