# Supplementary material for: The Huanan Seafood Wholesale Market in Wuhan was the early epicenter of the COVID-19 pandemic
Source: Science. 2022 Jul 26:abp8715. doi: 10.1126/science.abp8715 (PMC9348750; doi:10.1126/science.abp8715)
Supplement: Supplementary file 1 — Materials and Methods Supplementary Text Figs. S1 to S18 Tables S1 to S12 References ( 54 – 81 ) Data S1 and S2 [file science.abp8715_sm.pdf]

Science
AAAS

Supplementary Materials for

The Huanan Seafood Wholesale Market in Wuhan was the early epicenter of the COVID-19 pandemic

Michael Worobey *et al.*

Corresponding authors: Michael Worobey, worobey@arizona.edu; Kristian G. Andersen, andersen@scripps.edu

DOI: 10.1126/science.abp8715

The PDF file includes:

Materials and Methods
Supplementary Text
Captions and Links for Data S1 and S2
Figs. S1 to S18
Tables S1 to S12
References

Other Supplementary Material for this manuscript includes the following:

MDAR Reproducibility Checklist

Materials and Methods

Ethics Statement

This research was reviewed by the Human Subject Protection Program at the University of Arizona and the Institutional Review Board at The Scripps Research Institute and determined to be exempt from IRB approval because it constitutes secondary research for which consent is not required.

Sources of data

COVID-19 case data from December of 2019. The COVID-19 case data we consider here is that reported in the WHO mission report (*7*). These are all individuals that were hospitalized and notified to China's national disease surveillance system by hospitals in Wuhan during the early phase of the epidemic. Importantly, they are included as "December" cases based on their date of COVID-19 symptom onset, not their date of hospitalization.

Population density data from Wuhan, China. The dataset used for population density was downloaded from worldpop.org: China population 2020: constrained 100 m resolution: projection WGS84: Geotiff format: constrained datasets. This estimates population densities only within areas mapped as containing built settlements (*54*). The Geotiff file was imported directly to R and cropped to the administrative boundaries of Wuhan, using a shapefile downloaded from https://data.humdata.org/dataset/china-administrative-boundaries. The worldpop.org data gives an estimate of the number of people per pixel, with each pixel representing a 100 m by 100 m square defined by its center point. The resulting raster file for the Wuhan province has a total of 494,676 pixels. This raster file was transformed into a pixel image, from which the coordinates and values of each pixel were extracted.

To generate a population density data set of Wuhan that was age-matched to the December 2019 COVID-19 cases (for which age data were available in ten-year age bins), we downloaded worldpop.org datasets for population density by age and gender for 2020. These datasets are grouped in 5 year age intervals for male and female populations in Geotiff format: 100 m resolution; projection WGS84. Following the same methodology described above, these raster files were used to extract the coordinates and pixel values of each point. The datasets were merged to have population density data in 10 year intervals for both male and female populations to match the case data. To merge the datasets, first we verified that all the points' coordinates matched each other. Then, the pixel values for each point were added across all of the datasets to be merged. Population density null distributions were generated by sampling the different age groups in the same proportions as those reported by WHO for the December COVID-19 cases: 5.75% of the cases were sampled from the 80 and older age group population density data, 12.64% were sampled from the 70-80 age group population density data, 23.56% were sampled from the 60-70 age group population density data, 25.29% were sampled from the 50-60 age group population density data, 21.84% were sampled from 40-50 age group population density data, 7.47% were sampled from the 30-40 age group population density data and 3.45% were sampled from the 20-30 age group population density data.

COVID-19 case data from January and February of 2020. Geotagged Weibo COVID-19 help seekers' location data were shared with us by the authors (*26*) and consisted of 737 points defined by latitude and longitude. These were from users of a Weibo app designed for COVID-19 patients seeking medical information on the disease.

Extraction of December 2019 COVID-19 case locations

Geographical coordinates were not available for the residential locations of the 174 cases in Hubei province, China reported by the WHO mission to have had COVID-19 symptom onset in 2019 (all in December) (*7*). We therefore extracted locations from the 2-dimensional location data within the maps available in the WHO mission report and its annexes (*7*) using the following approach: We started with (1) Fig. 4 on page 148, Annex E of the WHO mission report (**fig. S1**, this study), which depicts the spatial distribution of 164 cases living in Wuhan by residential location, along with information on whether each case was epidemiologically linked to the Huanan market, and (2) a map of Wuhan adapted from one generated by us during an earlier study (*42*) (**fig. S2**). We gratefully acknowledge that data used to create that map of Wuhan was acquired from OpenStreetMap®, which is licensed under the Open Data Commons Open Database License (ODbL) by the OpenStreetMap Foundation (OSMF), ©OpenStreetMap contributors

(https://www.openstreetmap.org/copyright).

We then overlaid these two maps in Adobe Illustrator v25.4.1 (**fig. S3**) using the borders of Wuhan in each map to achieve a close overlap. While isolated case locations were easily discerned in WHO Figure 4 of Annex E, many were located in areas of high density. Moreover, the large circles used to indicate each residential location, with a diameter of approximately 2 km, plus the low resolution of the map, meant that many circles overlapped each other and could not be individually identified. Almost all other case maps in the WHO mission report had similarly large case markers and low resolution. Figure 23 on page 44 of the main WHO mission report (**fig. S4**, this study) was the exception and appeared to resolve cases better than other figures in the report, but it lacked borders of the city or other clear points of reference to use for reverse engineering the map's visual information into numerical coordinates.

We noticed, however, that we could overlay in Illustrator the more precise and higher resolution case location markers of the WHO report's Fig. 23 with the larger case location markers of WHO Fig. 4 such that the smaller markers were clearly placed in the center of the larger markers (**fig. S5**). This allowed us to combine the precision of WHO Fig. 23 with the points of the reference along the Wuhan border in WHO Fig. 4 and thereby use WHO Fig. 4 as a bridge between WHO Fig. 23 and the OSM map. This revealed that the WHO Figure 23 circles had diameters of roughly 250 m.

Next, we used WebPlotDigitizer v4.5 (Ankit Rohatgi; https://automeris.io/WebPlotDigitizer) to reverse engineer the case locations on the map in **fig. S5** and extract the underlying numerical data (latitude and longitude for each point). We used the map locations and longitude and latitude of the Huanan market [114.2576, 30.6196] and a point along the northeast border of Wuhan [114.9747, 31.0285] to define two X-axis and two Y-axis reference points (**fig. S6**). We then marked each case in WebPlotDigitizer, starting with the 124 central points in **fig. S7**, then moving to the more peripheral locations where individual locations on WHO Fig. 4, with its ~2 km diameter markers, were easily distinguishable. The inset in the top right of **fig. S7** shows a closeup of locations marked in WebPlotDigitizer where the halo of dark blue around each red location marker is the roughly 250 m-diameter location marker of each point from WHO Fig. 23. It is evident that the placement of the red markers using our approach likely generally introduces less than about 50 m of error.

Once all COVID-19 cases locations were marked, we converted the points into latitudes and longitudes in .csv format, using the reference points. We further annotated this file with information from (1) Figure 6 on page 78 of the main WHO mission report (*7*), which allowed us to discern the location of eleven lineage B cases and one lineage A case, and (2) Annex E Figure 17 (page 156), which allowed us to discern the locations of six family clusters. We assumed that each cluster involved two individuals (**fig. S8**). We also annotated the file to include, for the cases where such information was obtainable from WHO Fig. 23, whether a case was laboratory confirmed or clinically diagnosed.

Cases were to some extent obscured in the region closest to the Huanan market because of a high density of plotted locations even in the highest-resolution map in the WHO mission report (WHO Figure 23) - circles marking individual cases in some instances overlapped one another. Nevertheless, we recovered 155 of the 164 cases depicted in WHO Annex E Figure 4. The nine cases for which we were not able to extract a location therefore likely lie in the high case density area near the Huanan market.

Analyses of distances to the Huanan market

The spatial analyses below assume the possibility that indirect interactions between local residents and the Huanan market and its workers – for example at neighborhood stores, clinics, hospitals, schools, restaurants and other business as well as with other infected locals – could have led to initial foci of transmission in the residential areas immediately surrounding the market. Cases from an early point would not be restricted to these neighborhoods, but we hypothesized that if case location data came from an early enough time point in the outbreak, the geographical signal of the point source may not yet have been obscured. It is clear from time series data not considered here that such a diffusion process did occur generally from central Wuhan to more outlying areas (*42*).

Haversine distances to the Huanan market (30.61955, 114.25738) were calculated for each of the 155 December 2019 cases. The median distance from cases to the Huanan market was calculated separately for (1) all 155 cases, (2) the 35 cases epidemiologically linked to the Huanan market, (3) for the 120 cases not epidemiologically linked to the market, (4) the 65 confirmed cases, (5) the 79 clinically diagnosed cases, (6) the eleven lineage B cases, (7) the two lineage A cases and (8) a single lineage A case. We used medians rather than means for our analyses so as to not give undue

influence to outliers like those that can be seen in **fig. S8**. The lineage A cases were looked into both as a pair and as a single case because the non-hotel case is known to not be subject to ascertainment bias as it was recognized by Dr. Zhang Jixian at Xinhua Hospital before any notion of a connection of COVID-19 to the Huanan market existed (*5*) (see **Lineage 'A' and 'B' case locations**, below).

For the age-matched Wuhan population density data, the weighted median haversine distance to the Huanan market was calculated, with the weights assigned to each point being the pixel value of each point, which represents the population density for the 100 m by 100 m square with that point at its center. We also calculated the median haversine distance of the 737 Weibo help seekers from 8 January to 10 February 2020 (*26*).

To test whether the December cases were closer to the Huanan market than expected, null distributions were generated from the population density data and the Weibo data, with sample sizes matching the six groups of cases listed above for the December cases. For the population density data, sampling weights were assigned to each point, the weight being the pixel value, such that points representing higher population densities had a correspondingly higher probability of being sampled. For the population density nulls, 5.75% of the cases were sampled from the 80 and older age group population density data, 12.64% were sampled from the 70-80 age group population density data, 23.56% were sampled from the 60-70 age group population density data, 25.29% were sampled from the 50-60 age group population density data, 21.84% were sampled from 40-50 age group population density data, 7.47% were sampled from the 30-40 age group population density data and 3.45% were sampled from the 20-30 age group population density data. As noted, these percentages correspond to the percentage of December COVID-19 cases in each age group in WHO cases we analyze in this study.

For the lineage B eleven cases, the two lineage A cases and the single lineage A case the sample size was too small to accurately sample the corresponding percentages of cases from each age group population density dataset; an additional population density data set was generated by merging the seven age groups' population density data sets. To merge them, the pixel values from each point were added throughout all the different age groups, before adding each pixel value, they were assigned a weight corresponding to the proportion of cases from this age group that we would expect to sample. This weight was simply the proportion of early December cases that came from that age group. For each point in each pseudoreplicate the Haversine distance to Huanan was calculated, and the median or mean distance to Huanan was calculated for each pseudoreplicate. The median (or mean for n=2) distance between all the early December cases (n=155), the cases epidemiologically linked to the Huanan market (n=35), the cases not epidemiologically linked to the market (n=120), the confirmed cases (n=65), the clinically diagnosed cases (n=79), the lineage B cases linked to the market (n=11), and the lineage A cases (n=2) (as well as one of those lineage A cases analyzed on its own as a special case because it could not possibly have been subject to ascertainment bias) were compared to these null distributions. In the case of the single lineage A case and its corresponding null, instead of comparing median distances of each pseudoreplicate, this single distance was compared to the distance to Huanan from 1,000 single points drawn at random from the population density dataset null created with the methodology described above.

To test whether there was a difference in distance to the market between market-linked versus unlinked cases, we employed a Wilcoxon rank sum test.

Analyses of centering on the Huanan market

As a central tendency measure for groups of locations (*e.g.* the 155 COVID-19 cases locations from December 2019) we chose the coordinate-wise median latitude and longitude in order to reduce the influence of outliers (for convenience we refer to these as 'center-points'). Center-points were determined for the eight groupings of December COVID-19 cases listed in the section above, as well as for the population density data (worldpop.org) and the Weibo data (*26*). The Haversine distance between each resulting center-point and Huanan market was obtained. The population density center-point was estimated by taking the weighted median latitude and weighted median longitude of the population density dataset, assigning the pixel values as the weights, while the center-point of the Weibo data (*26*) was defined by the median of the 737 latitudes and the median of the 737 longitudes.

Significance testing of the distance between the December center-points and the Huanan market compared to the null distributions was based on random samples (with replacement) of 1,000,000 points from the population density data

(weighted). The 1,000,000 points were sampled from each population density age group, in the percentages mentioned above, which match the percentage of early December cases in each age group. 1,000,000 points were also sampled with replacement from the Weibo data (*26*). The Haversine distance to the Huanan market was calculated for each of these 1,000,000 points and these distances were compared to the distance to Huanan from the different center-points of each set of December cases (all cases, Huanan market-linked cases, or Huanan market-unlinked cases). These center-points represent plausible starting points of the COVID-19 epidemic in Wuhan, insofar as the center-point of early cases might reflect the starting point of the epidemic.

To further examine the distribution of December cases in relation to the Huanan market, kernel density estimates (KDEs) were generated for the market-linked cases, unlinked-cases and all cases, to infer a probability density function (PDF) from which the cases could have been drawn (function 'kde', package 'ks' version 1.13.4, R version 4.1.3, using default bandwidth selector). Contours representing specific probability masses (0.5, 0.25, 0.1, 0.05, and 0.01) were inferred and the location of the market compared to these. These contours contain the peak density with an increasingly small proportion of the total probability mass. Thus for the P=0.01 contour, a location drawn at random from the PDF is nearly 100-fold more likely to fall outside this contour than inside it and thus, if it does fall inside it is very unlikely to be this close to the peak density by chance.

December COVID-19 cases sensitivity analyses

We implemented an approach to investigate how robust the results of our geocoding analyses were to possible errors in locations extracted from the WHO mission report maps. As shown in **figs. S1 to S7**, we were able to reliably ascertain coordinates of points marked on maps in the WHO mision report, likely to within approximately 50 m. To explore how robust the results of our statistical analyses were to uncertainty in geocoded locations, we resampled each point randomly from anywhere within a circle of radius 1000 m, centered on our geocoded location. In other words, for each of the 155 points in our December COVID-19 case location data set, we introduced noise such that its location in sensitivity analyses could be up to 1 km from where we had placed it. We performed 1,000 iterations of adding noise in this way and ran the sensitivity analyses for all cases (n=155), for the Huanan market-linked cases only (n=35) and for the Huanan-market-unlinked cases only (n=120) (**fig. S9**).

We further examined the sensitivity of our analyses to both location "noise" and, simultaneously, to missing data. There were nine cases for which we were not able to extract a location, and these were likely from the region closest to the Huanan market, with a high density of plotted (and therefore likely overlapping) locations. To assess the impact of the missing data, we uniformly sampled nine additional locations from the 25% kernel density estimate of the 155 December cases in 1,000 iterations (reflecting the fact that our location extraction procedure likely missed cases in the high-density area) (see **fig. S10**).

Finally, we generated 1000 replicates sampling 104 cases from the set of 120 cases not linked to the Huanan market, to assess whether our results were robust to mis-assignment of some 'linked' cases as 'not linked' (**fig. S11**). As described above, we computed the median distance and the center-point distance to the Huanan market for each iteration and compared the distribution of these statistics to critical values of the distributions drawn from the Weibo locations and the age-matched worldpop.org samples. These analyses were performed (i) for the resampled locations, (ii) for the resampled locations with the additional samples representing missing cases, in both cases for all locations, for the locations of the cases linked to the Huanan market, and for the cases not linked to the Huanan market, and (iii) for the 104 sub-sampled cases not linked to the Huanan market.

Tolerance contour analysis

We studied variations in relative risk, $r(z) = f(z)/g(z)$, at each position z, where f(z) is the density of the test distribution, and g(z) is the density of the Weibo data distribution. We explicitly tested the null hypothesis H_0: $r(z) = 1$, against an alternative hypothesis of increased relative risk, H_1: $r(z) > 1$. Using an asymptotic *p*-value computation (shown to be more robust than a Monte Carlo approach) (*55*), we calculated and plotted contours corresponding to significant values of P(z), a pointwise estimate of statistical significance. For bandwidth estimation, we performed least squares cross validation on points within the same rectangular region as in **Fig. 1C** (Latitude:[30.4433,30.7466], Longitude:[114.0875,114.4741]),

which contains more than 92% (143 out of 155) of all early cases.

Lineage 'A' and 'B' case locations

We linked SARS-CoV-2 genome lineage information ('A' or 'B') (*30*) and residential location for twelve COVID-19 cases among the 155 from December 2019 for which we extracted locations - 11 lineage B (ten linked to the Huanan market and 1 unlinked) and one lineage A (**fig. S8**). Because we had earlier determined the location of the single lineage A virus (*5*) among the thirteen December cases for which genomic information was reported by the WHO mission report (*7*) (of which we were able to link twelve to particular cases) we were certain that the remaining eleven cases had been infected by lineage B. Location information was also available for one additional lineage A case with COVID-19 onset in 2019: virus 'Wuhan/WH04/2020'. This individual had stayed at a hotel near the Huanan market for the five days before fever onset (*32*). These are the two earliest-onset lineage A cases currently reported, and the eleven lineage B genomes are among the twelve earliest lineage B genomes. To investigate the distribution of hotels near the Huanan market, we plotted the distance from the market to the twenty nearest hotels using Bing Maps (**table S1**) and found that all were closer than 500 m. However, to be conservative, we assigned a distance-to-market for the hotel lineage A case of 2.31 km, equal to that of the other lineage A case in our data set. We treated this as comparable to a distance from a residential location to the Huanan market given the timing of the stay prior to symptom onset.

Robustness of statistical test results to possible ascertainment bias

To test the robustness of our results to the possibility of ascertainment bias, we took the following approach. For December 2019 COVID-19 cases (both 'all cases' and 'unlinked cases') we tested how many cases, starting with the one closest to the Huanan market and proceeding to the next closest, could be eliminated before losing statistical significance at the $\alpha=0.05$ level. Null distributions were generated from the population density data sets for the different age groups. Each age group population density data set was sampled in the same proportion as the ages of the early December cases reported by WHO mission report. The new median distance to Huanan after removing the cases nearest to the market was tested against these null distributions to obtain *p*-values. For the 'all cases' data set (n=155) the 98 nearest cases to the market could be removed without losing statistical significance (*p*=0.024). Significance was lost when removing the 99 nearest cases to the market (p=0.069). For the 'unlinked cases' (n=120) the 81 cases nearest to the market could be removed without losing statistical significance (p=0.045). Significance was lost when removing the 82 nearest cases to the market (p=0.088). The center-point of the cases was also calculated after removing the nearest cases to Huanan, from the 155 cases up to 38 cases could be removed without losing statistical significance (p=0.048), significance is lost when removing 39 cases (p=0.052). For the 120 cases unlinked to the market up to 36 cases can be removed without losing statistical significance (p=0.05), significance is lost when removing 37 cases (p=0.051).

Floorplan of the Huanan market.

Two maps of the internal arrangement of stalls in the Huanan Market are available: The CCDC map (*12*) (**data S1**) and the WHO map (*7*). While the two maps agree on key features, including the general arrangement of the stalls, neither map is drawn to scale. The internal location of stalls in the Huanan Market for our study was discerned through detailed analysis of satellite photographs (Google Maps, Google Earth, Baidu Maps), aerial photographs, and other images of the market. Use of Baidu Total View, which displays interactive panoramic images at street level, allowed for construction of an accurate detailed internal map of the locations of individual stalls. The outlines of the eastern and western sections of the market were traced from Google Maps using Adobe Illustrator. Locations of the key internal structural elements of the main areas of the Market, the vertical pillars, were determined from Total View images and other photographs then mapped onto the external walls of the eastern and western sections of the market. The western part of the market is approximately 70 meters wide, with pillars spaced an average of 8.75 meters apart (9 pillars per street), and the pillars along the main market building span about 126 meters, with pillars spaced about 9 meters apart (15 pillars total). Stalls on the north side of the canopy are supported by smaller metal pillars and are spread over the full market width of 70 meters and are each 6 meters deep. Additional stalls and storage units in the West market parking lot were not included, as they were not studied by either the CCDC or the WHO mission report. The eastern part of the market is approximately 92

meters at its widest, and the main building is approximately 90 meters long (supported by a series of 15 pillars). Distances were all confirmed using Google Earth's measurement tool. The panoramic images of Baidu Total View were then used to map the location of individual numbered walkways ("streets") throughout the market; major vehicle passages; and the dimensions of other major structures, such as the canopy in the eastern section of the market.

We also cross-checked our conclusions with an independent researcher's collation covering much of the same information (http://babarlelephant.free-hoster.net/visiting-the-wuhan-seafood-market courtesy of @babarlelephant). The map was then converted into geojson format for spatial analyses.

Analysis of environmental samples in the Huanan market

The location and quantity of positive environmental samples were taken directly from the CCDC map (*12*) (**data S1**), with the exception of two positive businesses in the southwest corner of the market that were only noted on the WHO map (*7*). Locations of known live animal vendors, cases, as well as businesses with no positive environmental samples were obtained from the WHO map (*7*). Testing of the significance of live animal vendors and/or human SARS-CoV-2 cases on the number of positive environmental samples found in a business was performed using a binomial GLM available in the 'stats' package in R. Distances between businesses were defined as the distance between their respective center-points. Spatial relative risk analysis was performed using the 'sparr' package in R, using linear boundary kernels for edge correction (*52*), with bandwidth selection performed using least squares cross-validation. Tolerance contours were calculated using the same robust asymptotic approximation used in **Fig. 2C** (*55*). Kernel density estimates of positive environmental sample density as well as sampled businesses were performed the same bandwidth identified from cross validation in the relative risk analysis.

Market stalls were assigned by categories of the types of goods sold through integration of several different sources. The WHO mission report(*7*) identified 10 stalls involved in the trade of live animals (Appendix F, Table 3). We further obtained names and descriptions of stalls from the TianYanCha.com business directory (**table S8**) based on stall addresses within the market. In many cases, business names and meat products sold were further confirmed through photographic evidence of stalls. Three stalls were identified as involved in domesticated wildlife sales from an official local forestry bureau fine for their registered owners for illegal hedgehog sales in summer 2019 (*36*). One of these two stalls (street 8, 25) yielded an environmental positive from the interior of a freezer, but was not noted as selling domesticated wildlife in Figure 2 from the "ANIMAL AND ENVIRONMENT STUDIES" section of the WHO mission report (*7*).

Analysis of human cases in Huanan Seafood Market

The location and timing of human cases was taken from the WHO mission report, with the exception of one case identified by media as well as in recent work. Analysis was performed using the 'sparr' package in R (*52*). Bandwidth selection for each kernel density estimate was determined by likelihood cross-validation.

Mobility analysis of the Huanan Seafood Market and potential superspreader sites within the city of Wuhan

To estimate the relative amount of intra-urban human traffic to the Huanan Seafood Market compared to other locations within the city of Wuhan, we utilized a location-specific dataset of social media check-ins in the Sina Visitor System as shared by Li et al. 2015 (*33*). This dataset is based on 1,491,499 individual check-in events across the city of Wuhan from the years 2013-2014 (5-6 years before the start of the COVID-19 pandemic), and 770,521 visits are associated with 312,190 unique user identifiers. We translated location names and categories to English using a Python API for Google Translate (see "**Data and code availability**"). Of the four markets reported to be selling a significant number of SARS-CoV-2 susceptible animals during 2019 (*8*), we found there had been 120 visitor check-ins to the Huanan Seafood Market during this time period, at least 81 to the Baishazhou Agricultural Products Market, 4 to Dijiao Flower and Bird Pet World, and 0 to Qiyimen Fresh Market. Compared to other markets across Wuhan (translated categories: 'supermarket', 'Convenience Store/Convenience Store', and 'shopping mall'), we found at least 70 other markets throughout the city of Wuhan that received more visitors than the Huanan Seafood Market (see "**Data and code availability**") (**fig. S12**).

Beyond markets, we found at least 1,676 total locations in Wuhan with more visitors than the Huanan Seafood Market. However, some high traffic locations may be less predisposed to COVID-19 superspreader events or substantial spread over a longer period than others: to further quantify this, we utilized a list of known SARS-CoV-2 superspreader locations/events (*34*) to further subset the following categories of locations that may serve as potential high-risk locations for superspreader events: 'Residential area', 'College', 'Building', 'shopping mall', 'Hospital', 'Middle school', 'supermarket', 'bar', 'Convenience Store/Convenience Store', 'Sports place', 'Comprehensive Stadium', 'church', 'Temple', 'primary school', ‘company’. This subset identified another further 430 locations which may be at higher risk for superspreader events, which received more human visitors than the Huanan Seafood Market. As a fraction of all social media check-ins to the set of 70 markets described above, the Huanan market represented (120/98,146) visits or 0.12%; as a fraction of all social media check-ins to the set of 430 locations similar to those of known superspreader events, the Huanan market represented (120/262,233) or 0.046%. For all four wet markets selling wild animals in Wuhan, these numbers were 206/98,146 (0.21%) and 206/262,233 (0.079%), respectively.

While the potential risk of a location to be the site of an ascertained COVID-19 superspreader event (SSE) undoubtedly depends on many factors beyond number of visitors, there are no reasons to believe the Huanan Seafood Market is at an unusually high risk of a SSE compared with several other locations in Wuhan. COVID-19 cases associated with the Huanan market were not older (and actually leaned slightly younger) than all December 2019 COVID-19 cases on average (*7*), indicating the market population was not excessively elderly. Further, the main entryways to the market were large and open to the street, indicating a significant degree of airflow through the main thoroughfares.

While the association of social media check-ins and true visitor number likely varies across different types of sites and is likely subject to demographic biases, for the Huanan market to be even a remotely likely random location for a superspreader event within the city of Wuhan would require it to be extremely under-reported in the social media data. The fraction of Huanan market social media visitors out of social media visitors to all markets was 0.12%, or slightly higher than the number of visitors per day officials reported to the WHO mission report (10,000) as a fraction of the general Wuhan population of approximately 11 million (0.09%). Further, the Huanan market specifically received fewer social media visitors than 2 Walmart stores, 2 Carrefour stores, and 1 RT-Mart store, and does not stand out among other large wholesale markets in the city.

Furthermore, in light of the strong evidence for independent introductions of lineages A and B, and the association of both with the Huanan market, these check-in data suggest that independent introductions of these lineages at the same, relatively seldom visited location in Wuhan, would be extremely unlikely to occur by happenstance.

Supplementary Text

Results comparing to Weibo empirical distribution, and comparing laboratory confirmed vs clinically-diagnosed cases

In addition to comparisons of the spatial distribution of the early COVID-19 cases to the age-matched Wuhan, we further compared these cases with the empirical distribution of Weibo COVID-19 help seekers from, as a proxy for the distribution of cases within the city during the later phase of the pandemic. We found that early COVID-19 cases had a significantly closer median distance to the Huanan market than Weibo help seekers later in the pandemic. This was true considering all cases (median 4.28 km, $p<0.001$), Huanan-unlinked cases (median 4 km; $p<0.001$), Huanan-linked cases (median 8.3 km; $p<0.031$), and the two early lineage A cases (median 1.33 km; $p<0.002$). Further, the early cases also had significantly closer geographic center-points to the Huanan market than Weibo help seekers later in the pandemic. This was also true considering all cases (center-point distance 1.02 km; $p<0.015$), Huanan-unlinked cases (center-point distance 0.91 km; $p<0.005$), and the two early lineage A cases (center-point distance 1.12 km; $p<0.009$).

The WHO mission report on the origins of SARS-CoV-2 noted that early COVID-19 cases were either laboratory confirmed (by sequencing, PCR, or serology) or clinically confirmed based on clinical characteristics. We further tested cases based on each case definition separately and found that both laboratory confirmed and clinically confirmed cases had significantly closer median and center-point distances to the Huanan market than the Wuhan general population or the Weibo help seekers.

Laboratory-confirmed cases resided a median of 2.91 km from the Huanan market, while clinically-diagnosed cases resided a median of 4.67 km away. Both were closer to the market than the Wuhan population distribution and Weibo distribution ($p<0.001$ for all four combinations). The center-point of the laboratory-confirmed cases was 0.32 km from the Huanan market, closer than the Wuhan population distribution ($p=0.0007$) and the Weibo cases ($p=0.002$). The center-point of the clinically-diagnosed cases was 1.80 km from the Huanan market, closer than the Wuhan population null distribution ($p=0.022$) and Weibo data ($p=0.038$)

All p-values at the 0.05 significance level were still significant after applying the Benjamini-Hochberg Procedure for multiple hypothesis correction (**table S4**).

No other location except the Huanan market clearly epidemiologically linked to early COVID-19 cases

No other location in Wuhan was, at the time or retrospectively, identified as being clearly epidemiologically linked to the December 2019 COVID-19 cases (*42*). For example, a major transit hub, the Hankou Railway Station, is located near the Huanan market; but if mass transit was the route of entry into Wuhan, it would still require the establishment of infection at the Huanan market to be consistent with the results presented here, and, while numerous early COVID-19 cases where detected among workers at the Huanan market, none were reported among workers at this station (or any other major transit hub in Wuhan). Likewise, the Wuhan CDC (WCDC) is very close to the market's location, but there was *"no storage nor laboratory activities on CoVs or other bat viruses preceding the outbreak*" at that site (*7*) and no epidemiological evidence of COVID-19 cases linked to the WCDC in December 2019 or earlier (*7*). Beyond the Huanan market, no other proposed or hypothesized origin has been supported by data (*42*, *56*).

Animal screening for SARS-CoV-2 in China

WHO mission members were told (despite known susceptible live animals including raccoon dogs being mentioned in the agreed terms of reference for the study's scope) that no unlicensed or live-trapped wild animals had been for sale at the Huanan market and that *"no verified reports of live mammals being sold around 2019 were found"(7)*. Notably, none of the live (known to be susceptible) mammals from species we identify here as present at the Huanan market in November 2019 have been reported to have been tested for evidence of SARS-CoV-2 infection. The only live mammals 'from' the market among the 188 appear to have been animals such as stray cats, dogs, snakes, rabbits, and mice. Indeed, of the 188 animals tested, 167 were those species or hedgehogs, pigs, chickens, salamanders, crocodiles, turtles, fish or sheep (*7*). Only 21 individuals from traded mammalian species likely susceptible to SARS-CoV-2 infection were tested – but these were dead, of unknown procurement date, and refrigerated or frozen, and therefore unlikely to be a source of human infection: six "*bamboo rat*", six "*muntjac*", six "*badger*", two "*wild boar*" and one "*weasel*" (*7*). Some of the wild

mammals were screened (negative) for active SARS-CoV-2 infection by qRT-PCR, but a very low number have sampled from Hubei province (*57*). In addition, bamboo rats, porcupines, and wild boar from farms in Hubei supplying the Huanan market, sampled during February and March 2020, also showed no sign of active infection (*7*). Unfortunately, to our knowledge, no serological testing was conducted on animals from within the market or from farms supplying it, nor on farm workers or animal traders in those supply chains. Indeed, both the Huanan market and the farms in Hubei province that supply it (*48*) were rapidly shut down before such sampling occurred. Hence, it is apparent that by the time the Huanan market was closed, and animal sampling at the market began, the SARS-CoV-2-susceptible live mammals that had been on sale there in the preceding months (**Tables 1 and S5**) were no longer present (*7*).

While >80,000 animal samples were tested and no evidence was found for presence of, or exposure to, SARS-CoV-2 (*7*), this is far from conclusive evidence for most animals. The negative predictive value of the surveys depends on the assumed prevalence. For serology, for instance, if one assumes a reservoir host in which the virus is endemic, seroprevalence likely is high, but will depend on the age of the animals and infection dynamics in that particular species. In addition, China is a vast country, and the possible sources of animals could be over a long distance. Therefore, the catchment area for surveillance studies is huge (*58*). Therefore, tracking back along the market supply chain is crucial for a more targeted effort, as recommended in the WHO mission report (*7*). In addition, it is possible that a one-off spillover event occurred on a farm that supplied the market. Such events would be needles in a haystack that are virtually unresolvable.

A summary of data on the size of the early epidemic within Wuhan

Not until the second week of December 2019 was there a detectable increase in the number of reported ILI-like cases in Wuhan (WHO mission report, Epidemiology, Figure 1). However, even this increase in ILI-cases was consistent with an increase in laboratory confirmed Influenza A and B cases, that occurred primarily in children (see WHO mission report, Epidemiology, Figure 2C) until the last two weeks of December, when an increase in adult ILI-cases was also observed. Consistent with this, retrospective testing of 640 throat swabs collected at the Children's Hospital of Wuhan and Wuhan No. 1 Hospital from Oct 6 2019 to January 21 2020 only identified nine positive SARS-CoV-2 samples, the earliest of which had reported symptom onset of January 4th, 2020 (*59*). Meanwhile, 22% of these cases tested positive for Influenza. A further 2334 throat swabs from the Wuhan Tongji hospital system and 218 throat swabs from Wuhan Union Hospital, collected in Oct-Dec 2019, were additionally retrospectively tested and found to all be RT-PCR negative for SARS-CoV-2 (see WHO mission report, Review of Stored Biological Samples Testing). Furthermore, travel cases exported from Wuhan can also be used to estimate the number of infections within the city during the early phase of the epidemic. Out of 117 influenza-like illness cases who traveled from Wuhan to Hong Kong from Dec-31-2019 to Jan-21-202, only 1 (0.9%) was positive for SARS-CoV-2, while 36.3% were positive for seasonal influenza (*60*). Of 2164 blood donations within the city of Wuhan that were collected before January 23 and retrospectively tested for SARS-CoV-2 antibodies, only one sample, from January 20, 2020, was confirmed to be SARS-CoV-2 seropositive (*44*), providing further evidence that the epidemic was still limited in size by early to mid January 2020. Similarly, 2058 plasma samples collected from patients at Tongji Hospital in Wuhan between July and December 2019 were tested for SARS-CoV-2 antibodies, and all were found to be negative (see WHO mission report, Review of Stored Biological Samples Testing). Excess all-mortality and pneumonia deaths, a lagging but robust signal of COVID-19 cases resilient to possible reporting issues, only exceeded rates of previous years in Wuhan by the week of Jan-14-2020, consistent with an epidemic growing substantially in size only from the end of December 2019, and a 17 day median time from onset of illness to death for COVID-19 (see WHO mission report, Epidemiology, Figure 12).

Additional data related to case ascertainment biases

We investigated the possibility of whether ascertainment biases in case identification could alternatively have shaped the geographic distribution of early cases throughout the city of Wuhan. In particular, three possible ascertainment biases are (i) a direct ascertainment bias towards the Huanan market, where cases were directly diagnosed based on their reported direct contact with the market, (ii) a path-dependent contact tracing ascertainment bias, where cases unlinked to the market were identified primarily through contact tracing of cases linked to the market, and (iii) a geographic

ascertainment bias, where cases unlinked to the market were identified primarily through selective reporting by hospitals or by selective case reporting based on residential addresses. First, we note that the strong geographic association between unlinked early cases and the Huanan seafood market, which cannot be explained by the demographics of the city of Wuhan, importantly cannot be explained by a direct market ascertainment bias, as no market link for these cases was ever ascertained. We further identified several data points inconsistent with a significant impact of either contact tracing or geographic ascertainment biases on the early case data, summarized below.

Districts near the Huanan seafood market had elevated seropositivity in a retrospective analysis. If COVID-19 cases were overreported in neighborhoods close to the Huanan seafood market, we would expect a discrepancy between the number of reported cases and seropositivity by neighborhood (neighborhoods around Huanan market would have low seropositivity despite their high reported case numbers). However, a retrospective serosurveillance study (*40*) in April 2020 reported that the district where the Huanan seafood market resides (Jianghan) and the adjacent districts north of the Yangtze river (Jiang'an and Qiaokou) had higher rates of seropositivity compared to districts south of the Yangtze river (Hongshan, Wuchang, and Qingshan), consistent with the districts adjacent to the Huanan seafood market being characterized by earlier and therefore larger epidemic curves (**table S3**). Seropositivity by district correlated well with the number of reported cases per capita for each district by March 2020, giving no indication of case overreporting in districts near the Huanan seafood market or underreporting in districts south of the Yangtze river or elsewhere in the city (*40*); (**table S3**). A separate serosurvey of the city reported similar results (*41*) (**table S3**). Excess COVID-19 deaths (*61*) and deaths due to all pneumonia (WHO mission report, Figure 21) were also first elevated in the districts north of the Yangtze river near the Huanan seafood market by mid-January, 2020.

Retrospective ILI-testing for SARS-CoV-2 also identified early patients living near Huanan market. A retrospective analysis of 640 throat swabs collected at two hospitals in Wuhan from Oct 6 2019 to Jan 21, 2020 identified 9 SARS-CoV-2 positive samples (*59*), with dates of onset Jan 4 to Jan 20th. Seven of these patients lived within the boundaries of Wuhan city, and all 7 of the early JanuarySARS-CoV-2 positive patients lived north of the Yangtze river in Jianghan district or adjacent districts.

*Reported cases not directly linked to Huanan market lived significantly closer to the market than linked cases***.** If cases with no identified link to Huanan market were primarily ascertained through contact tracing of personal contacts of Huanan-linked cases (a path dependent ascertainment bias), they would be expected to have a similar geographic distribution as cases linked to the market. On the contrary, we note that on average they lived closer to the market (4.00 vs 5.74km, p=0.029). This pattern is thus better explained if the primary risk factor for linked cases was their place of work, while the primary risk factor for unlinked cases was proximity to the market and community spread starting at the market.

Publications reporting on early cases do not utilize case criteria dependent upon market links. We identified four studies that first reported on cohorts of cases with timing of onset in December 2019 (**table S2**). Only one of these papers (*62*) reported an epidemiological criteria in their case definition. This criterion was secondary to clinical characteristics, as they included patients with only 3/4 clinical characteristics if they also had a link to the Huanan Seafood Market. None of the studies reported case definitions or patient inclusion based on their neighborhood of origin or patient area of residence within the city of Wuhan, and thus none of these case definitions could directly influence a geographic association of officially unlinked cases with the market. Chen et al. (*39*), describing a cohort of patients moved to Jinyintan Hospital for isolation, reported that "adult patients were admitted centrally to the hospital from the *whole* of Wuhan *without selectivity*" [emphasis ours] and also affirmed that their data had been shared with the WHO.

Both clinically confirmed cases and laboratory confirmed cases were geographically centered around the market. If direct or indirect market-associated epidemiological criteria heavily influenced the "clinical diagnosis" case definition used in the WHO mission report, then we might expect that the laboratory confirmed cases would have a strikingly different spatial distribution. However, we have updated our analysis to confirm that cases identified under both definitions are geographically associated with the Huanan seafood market. (See section "**Results comparing to Weibo empirical**

distribution, and comparing laboratory confirmed versus clinically-diagnosed cases", above.)

Both early viral lineages A and B were observed in proximity and directly connected to the Huanan market. If the majority of sequenced early SARS-CoV-2 cases were subject to a strong path dependency ascertainment bias in relation to the Huanan market, then we would not expect to observe the ancestral viral genotypes in patients and environmental samples with connection to the market. Rather, we would expect to observe these genotypes and their descendants in at least a few patients without connection to the market. We would primarily expect samples with connection to the market to be descendant lineages. However, genomes of SARS-CoV-2 lineages A and B — the two lineages that were likely introduced into humans separately (*38*) — have been epidemiologically linked to the market, both in this work, and via direct environmental sequencing (*24*).

Hospitals reporting early cases were distributed throughout the city of Wuhan. One mechanism by which a geographic ascertainment bias could arise is if there was significant selective reporting of cases in hospitals adjacent to the Huanan seafood market, and patients tended only to visit hospitals near their home. However, early cases were reported by several hospitals scattered throughout the city (**fig. S11**). Some of these hospitals were further from the Huanan market than the mean distance of case residential addresses to the market. In addition, Chen et al. (*39*) reported that cases were sent to Jinyintan Hospital from the whole of Wuhan "without selectivity". Therefore, the geographic association of the residential addresses of reported cases with the Huanan seafood market was not due to hospital proximity.

On 29 December 2019, the first day public health officials learned of a possible association of COVID-19 cases with the Huanan market, they simultaneously learned of cases not only at Xinhua Hospital, near the Huanan market, but also Tongji and Union Hospitals, far to the south. There appears to have been no period of time when public health officials could have selectively searched only in hospitals near the market for cases linked to the Huanan market; they knew linked cases were being found further afield as well. Indeed, Wuhan CDC was alerted to the outbreak of unexplained pneumonia and its association with the Huanan market by a vice president of Xinhua Hospital (fairly near the market) whose tipping point appears to have involved learning about Huanan market-linked cases at hospitals many kilometers from the market: "Upon learning of similar patients, also linked to Huanan Market, at Tongji and Union (Xiehe) Hospitals, Xia alerted the Wuhan and Hubei CDCs on 29 December" (*5*).

This is 'all-hospital' search, moreover, is reflected in an internal communication from the Wuhan Municipal Health Commission to *all* hospitals in Wuhan the very next day, on Dec 30, and in the follow-up announcement on 3 January 2020 for hospitals be on the lookout for unexplained pneumonia in any patient, not just ones with a link to the Huanan market. Accordingly, by 3 January, it is clear that the major hospital in Wuchang district, Zhongnan Hospital, was detecting both Huanan-linked and unlinked cases (*5*). And even though it was far from the market, of the first trickle of cases identified at this hospital, two were linked to the Huanan market, with the other three being a family cluster. The facts that initial cases at this major hospital, distal from the market (1) lagged considerably behind those at hospitals closer to the Huanan market (7 at Xinhua Hospital by 29 December and 7 at Wuhan Central Hospital Houhu Branch by 28 December (*5*)) and (2) nevertheless involved large proportion linked to the Huanan market, provide further evidence that the outbreak indeed started at the Huanan market then spread into the wider Wuhan community from there.

All the cases we consider in this study were hospitalized and, therefore, were likely ascertained in hospitals rather than in the community. It is possible that public health officials could have identified previously undiagnosed individuals in hospitals via contact tracing from Huanan-linked cases (i.e., patients that clinicians had not yet identified as unexplained pneumonia cases linked to the outbreak, but which public health officials had traced from other, previously identified, pneumonia patients). However, as pointed out above, the different geographic distribution of cases unlinked to the market (they lived closer to market) than those linked to the market argues persuasively against ascertainment bias in the detection of those unlinked cases; we would expect the unlinked cases to be drawn from a similar geographic distribution as the linked cases if they had been ascertained through contact tracing. So, could those unlinked cases have been detected via biased case-finding involving searching for cases only in neighborhoods near the market but not in other parts of Wuhan? Not likely. Remembering that all the cases were hospitalized and that no diagnostic test was available to identify

mild cases it is likely that most or all Huanan market-unlinked cases were ascertained while in hospitals.

Robustness of the December COVID-19 case analyses using center-point and median distances to the Huanan market

We performed three sets of sensitivity analyses for the tests using center-point and median distances to the Huanan market (cfr. Materials and Methods). Adding considerable random noise to the extracted locations results in distance statistics that are similar to those inferred from the actual extracted locations relative to the critical values of the null distribution (**fig. S13**). The same holds true when we add noise as well as missing locations from relatively close to the Huanan market (from the 25% kernel density estimate of the 155 December cases, cfr. Materials and Methods) (**fig. S14**). Finally, sub-sampling the cases not linked to the Huanan market also indicates that the test approach is robust to some degree of mis-assignment of cases (**fig. S15**).

Supplementary Data

Data S1. Translations and URLs for relevant articles and reports

All translations were done using the premium service at TheWordPoint (https://thewordpoint.com/). Originals can be accessed via the original URLs or WayBackMachine URLs provided in the “documents_index.csv”. File is available from https://doi.org/10.5281/zenodo.6291868.

Data S2. Business registry screenshots from Tianyancha

File is available from https://doi.org/10.5281/zenodo.6791326.

Supplementary Figures

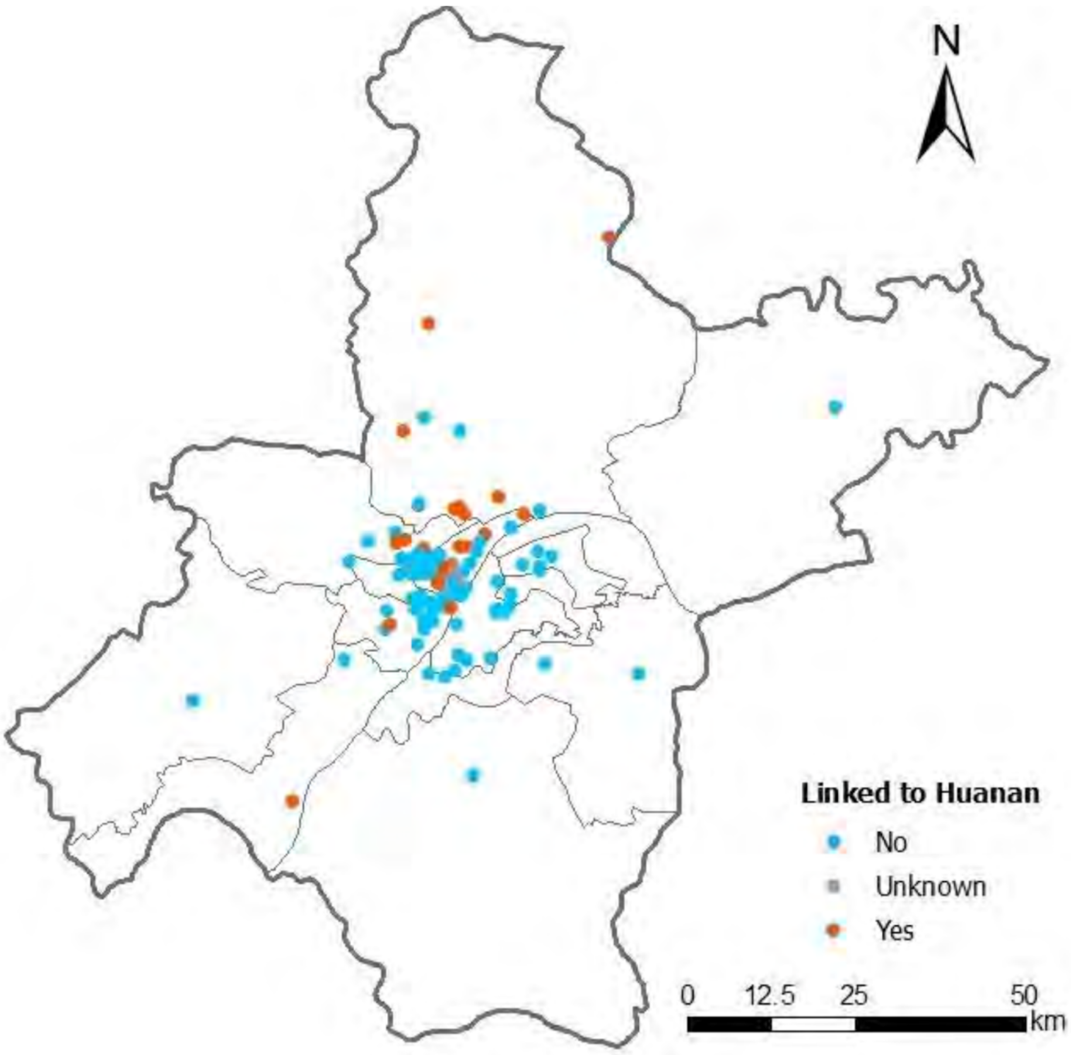

<u>Figure S1. COVID-19 cases in Wuhan in December.</u> This is Fig. 4 on page 148, Annex E, of the WHO mission report.

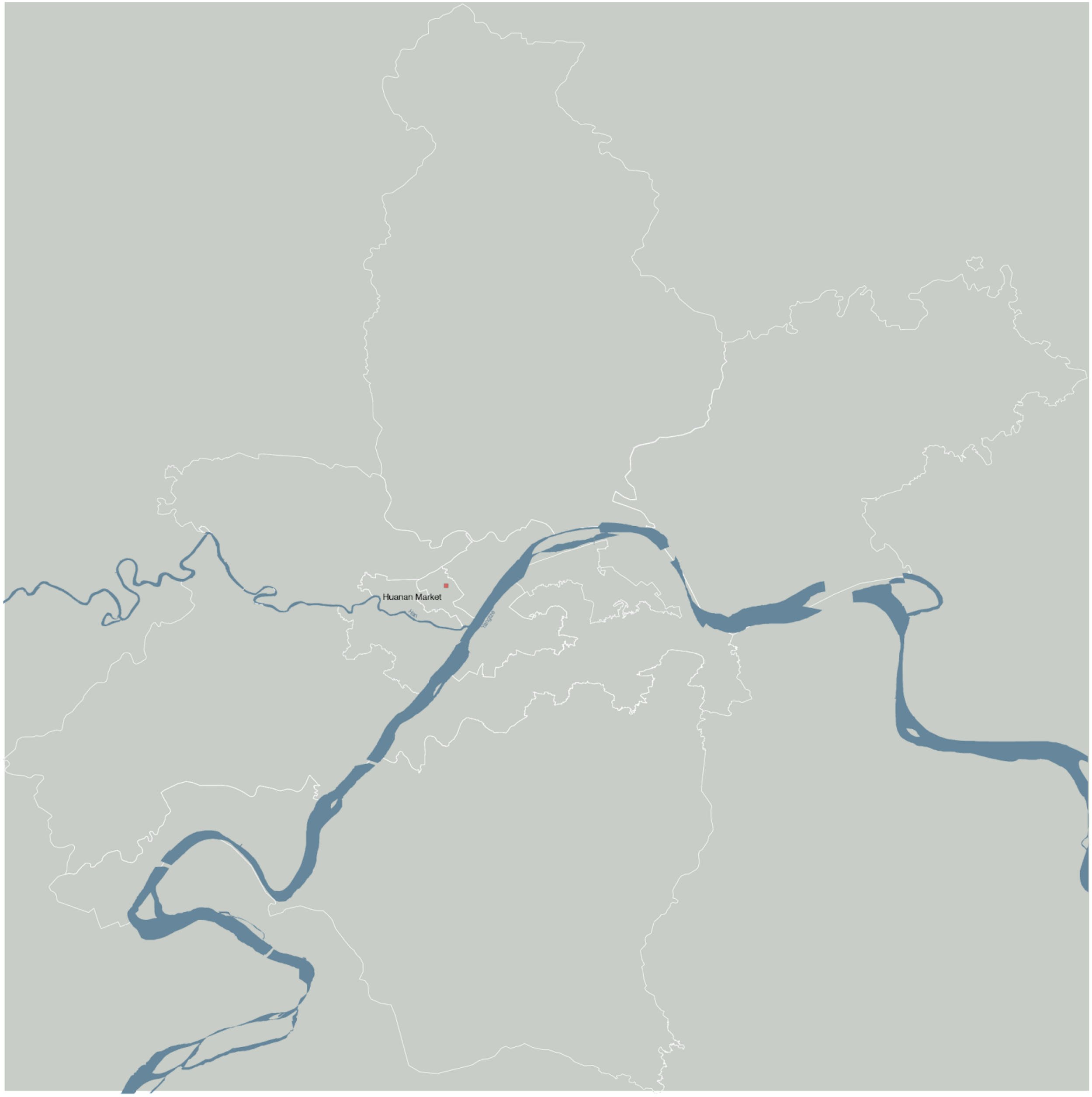

Figure S2. Map of Wuhan. Credit: © OpenStreetMap contributors.

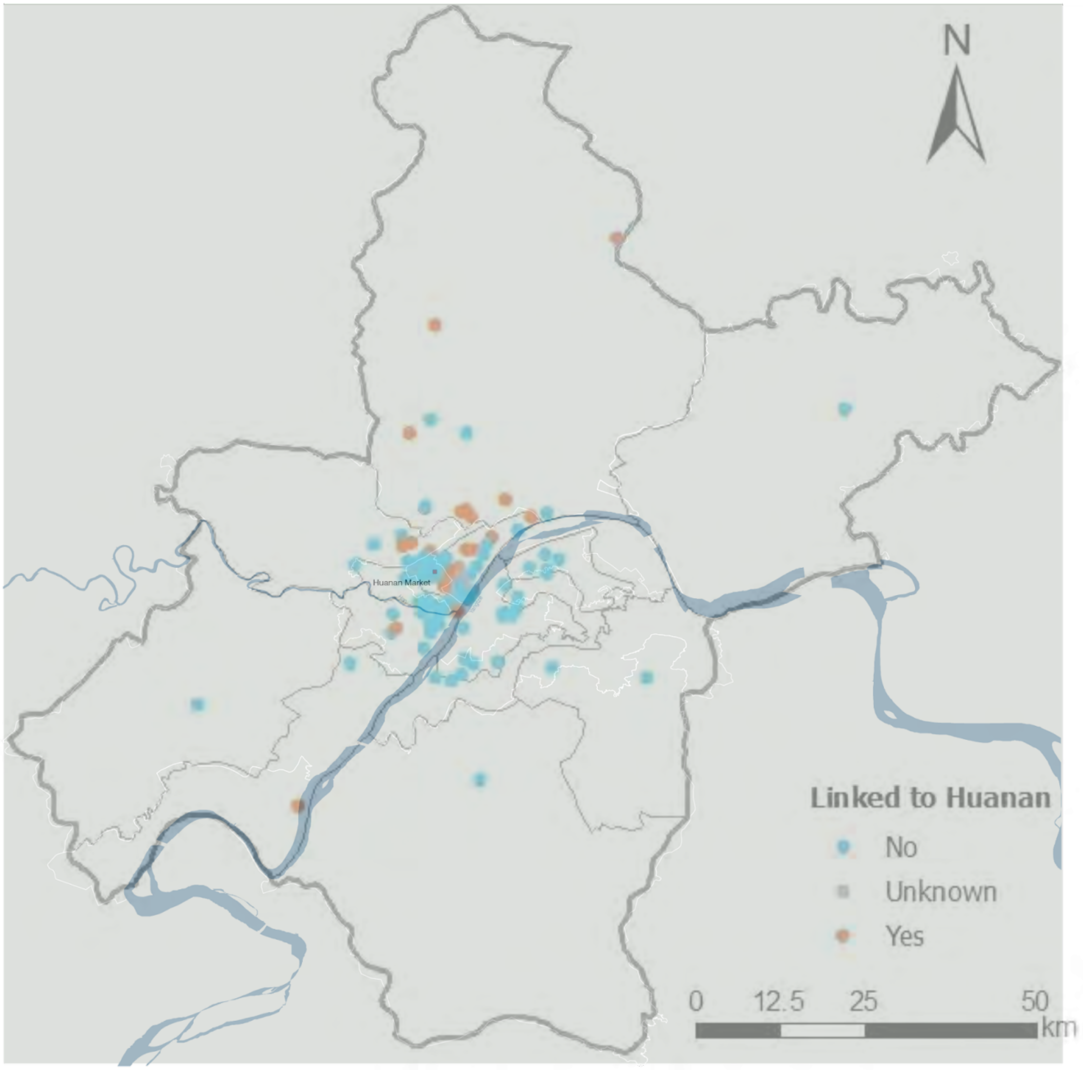

<u>Figure S3. Overlay of Figs. S1 and S2.</u>

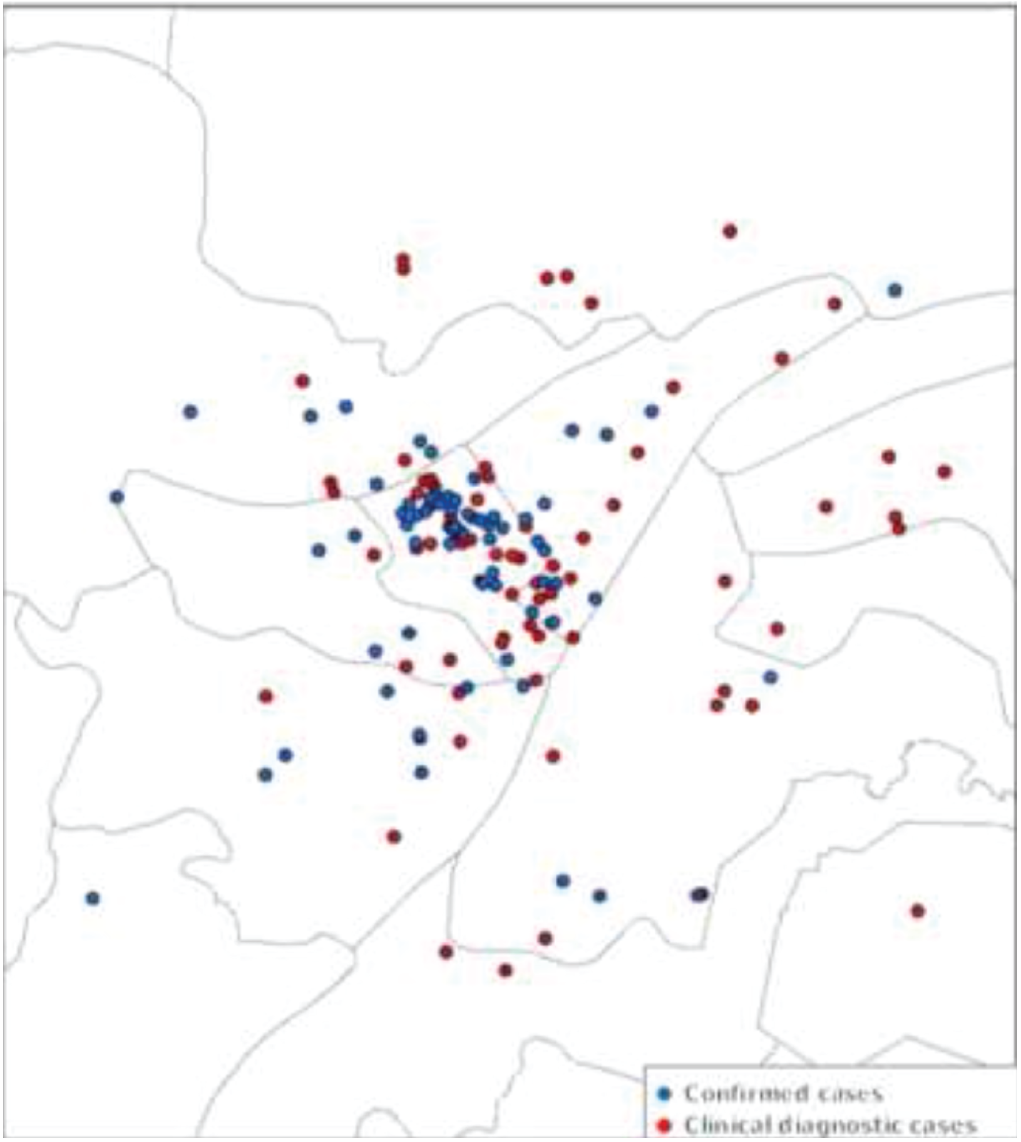

<u>Figure S4. Fig. 23 page 44 from the WHO mission report.</u> The radius of each circle marker of each case location is about 125 m.

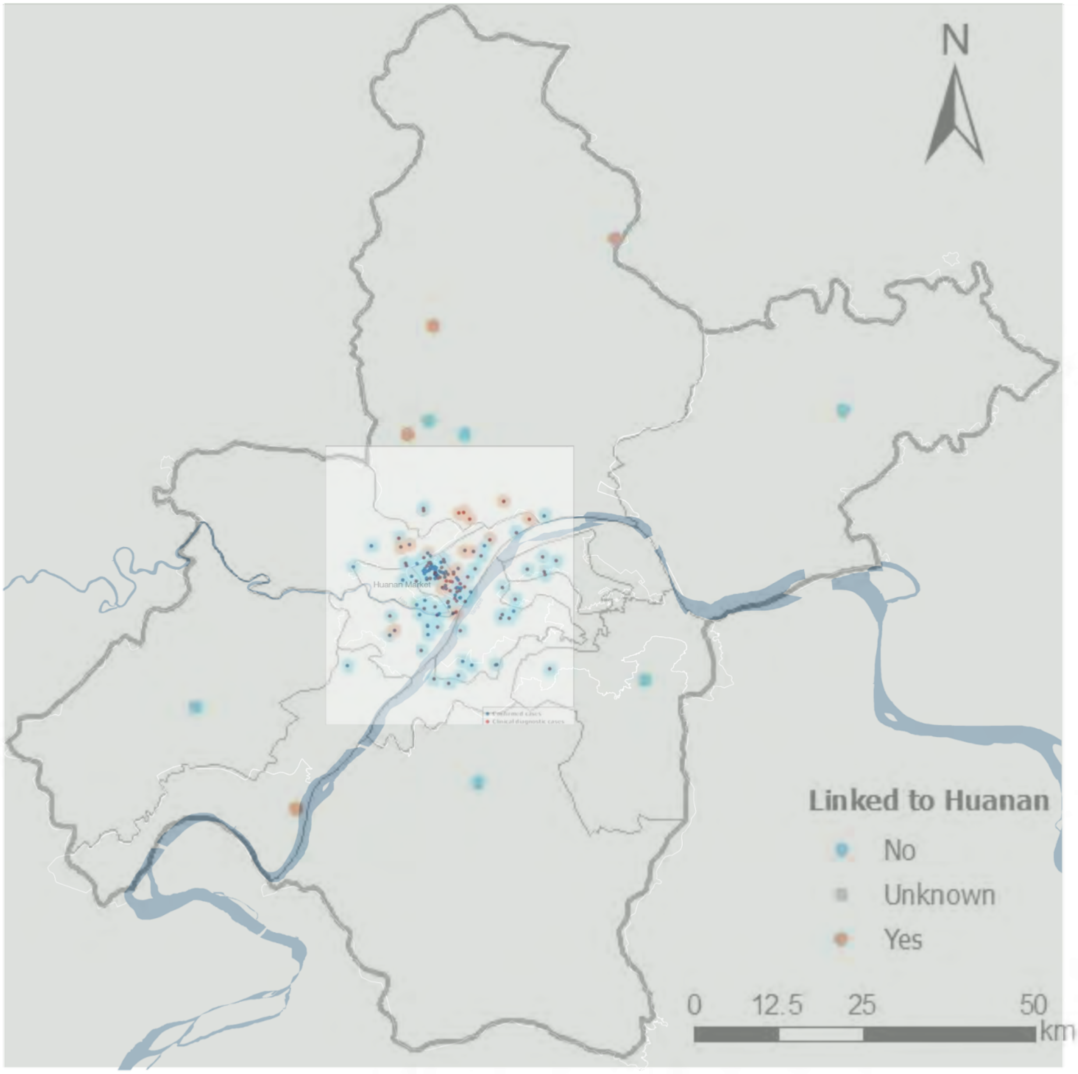

Figure S5. Overlay of fig. S3 and Fig. 23 from page 44 of the main WHO mission report.

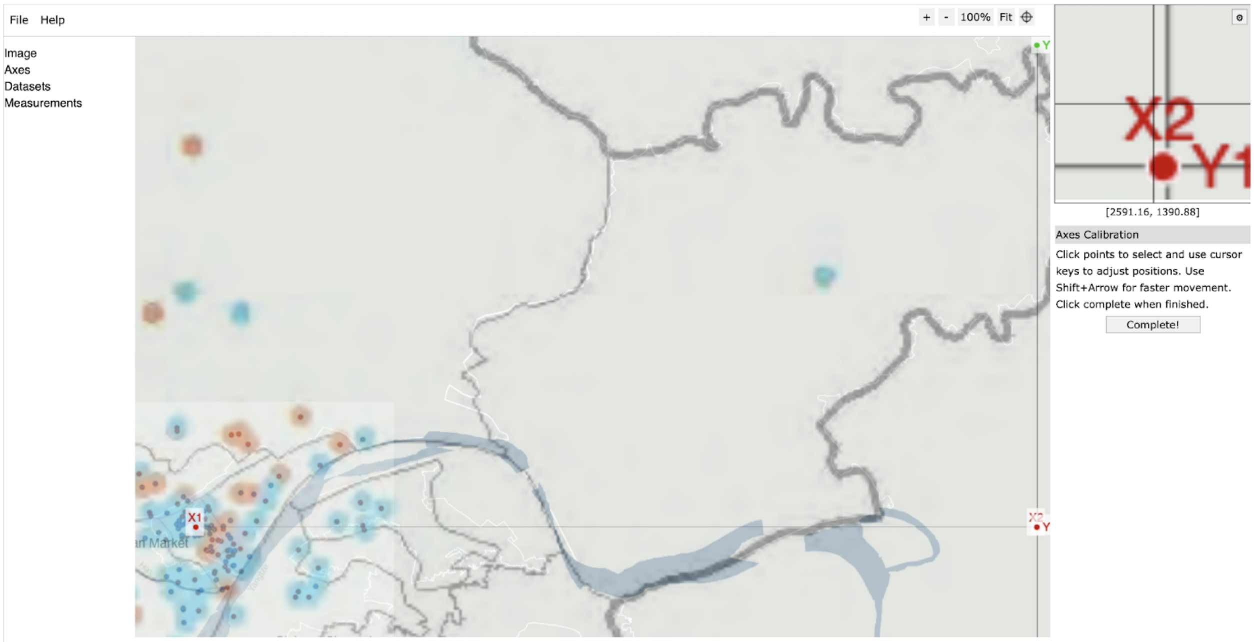

Figure S6. WebPlotDigitizer image, showing how reference points are input.

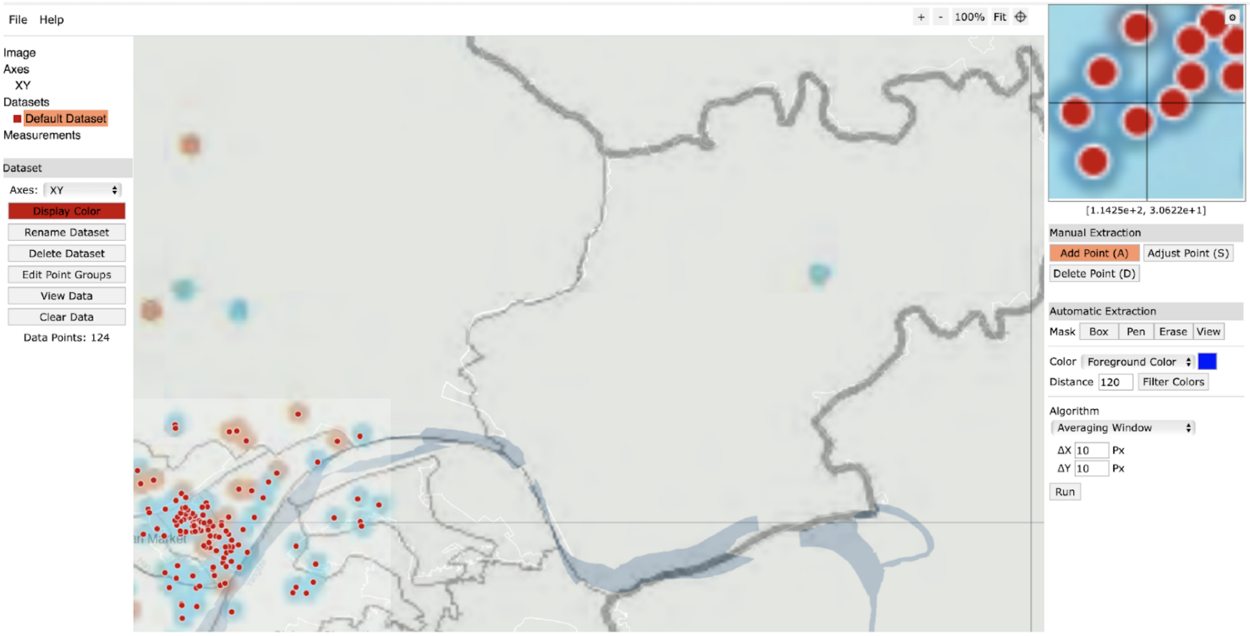

Figure S7. WebPlotDigitizer image, showing how points are added. The red dots have been added in WebPlotDigitizer to mark the darker blue dots, whose halo appears around the red dots (inset in top right). This illustrates the high precision with which case locations were extracted.

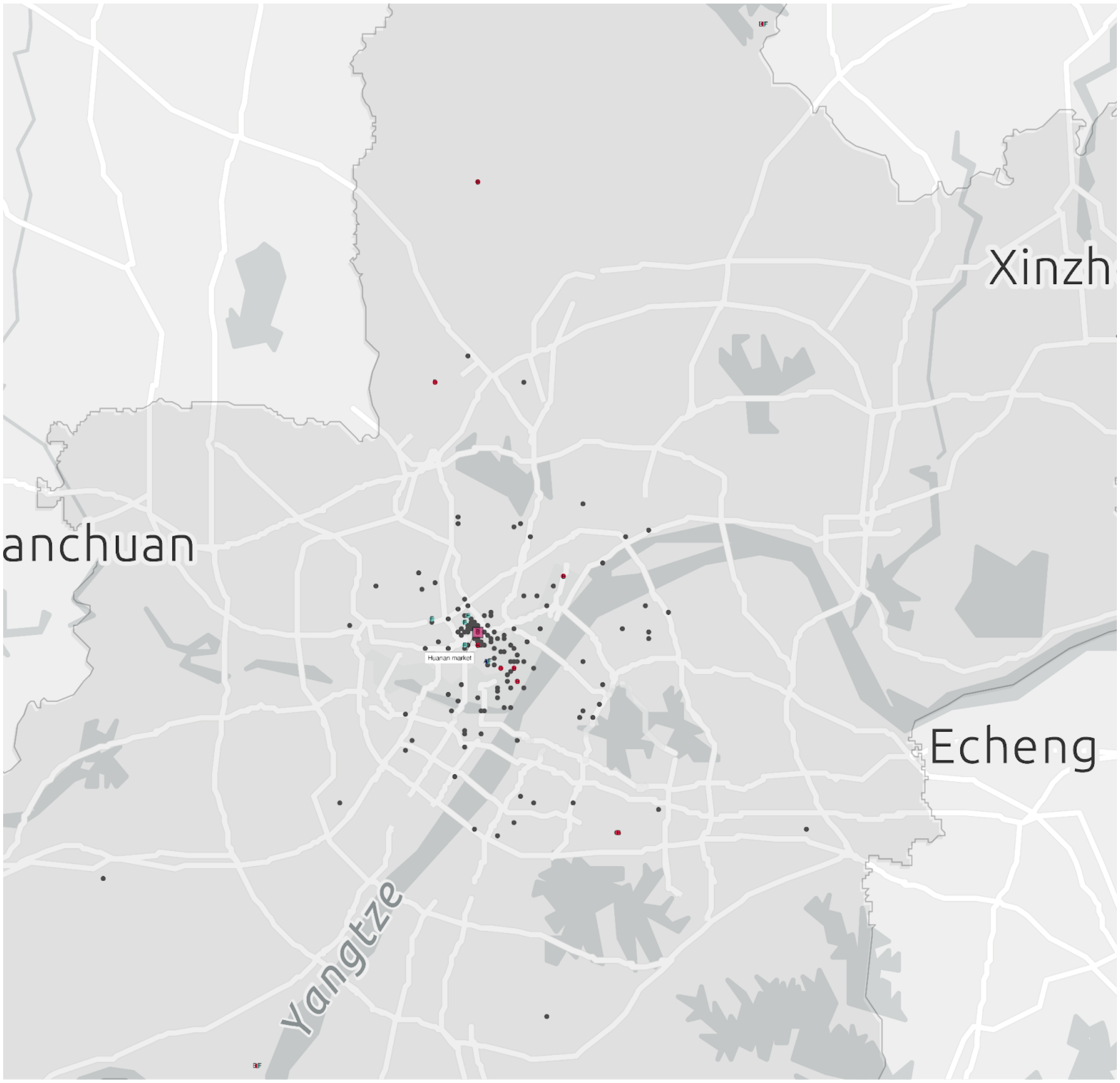

Figure S8. Case map showing family cases and lineage A and lineage B genome sequences. F=family case. B=lineage B case. A=lineage A case.

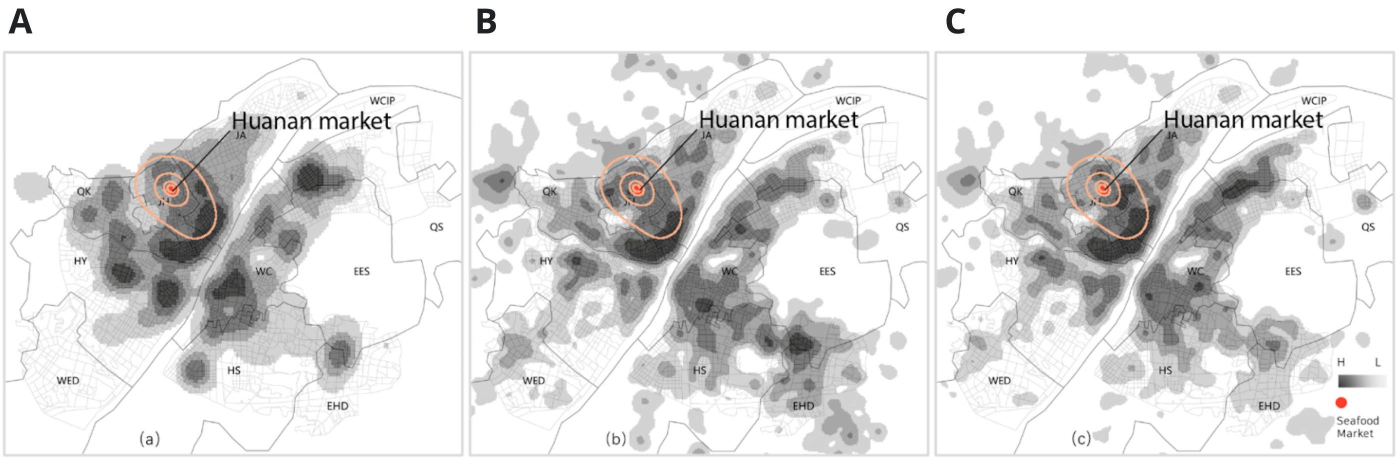

Figure S9. Comparison of highest density area of COVID-19 cases in December 2019 to density of cases, overall population and elderly population in Wuhan in January and February 2020. This figure has been adapted slightly from Peng *et al*. (*26*) by the addition, in panels **A** through **C** of colored polygons representing the highest density 50%, 25%, 10% and 1% KDE contours of December 2019 COVID-19 cases from the current study's fig. 1C. The red circle indicates the location of the Huanan market and is part of the original figure. As described in Peng et al. (*26*), panel **A** represents COVID-19 help seekers by Weibo data, **B** population density, and **C** elderly population density. Peng et al. retain copyright and the figure is made available under the CC BY 4.0 International License.

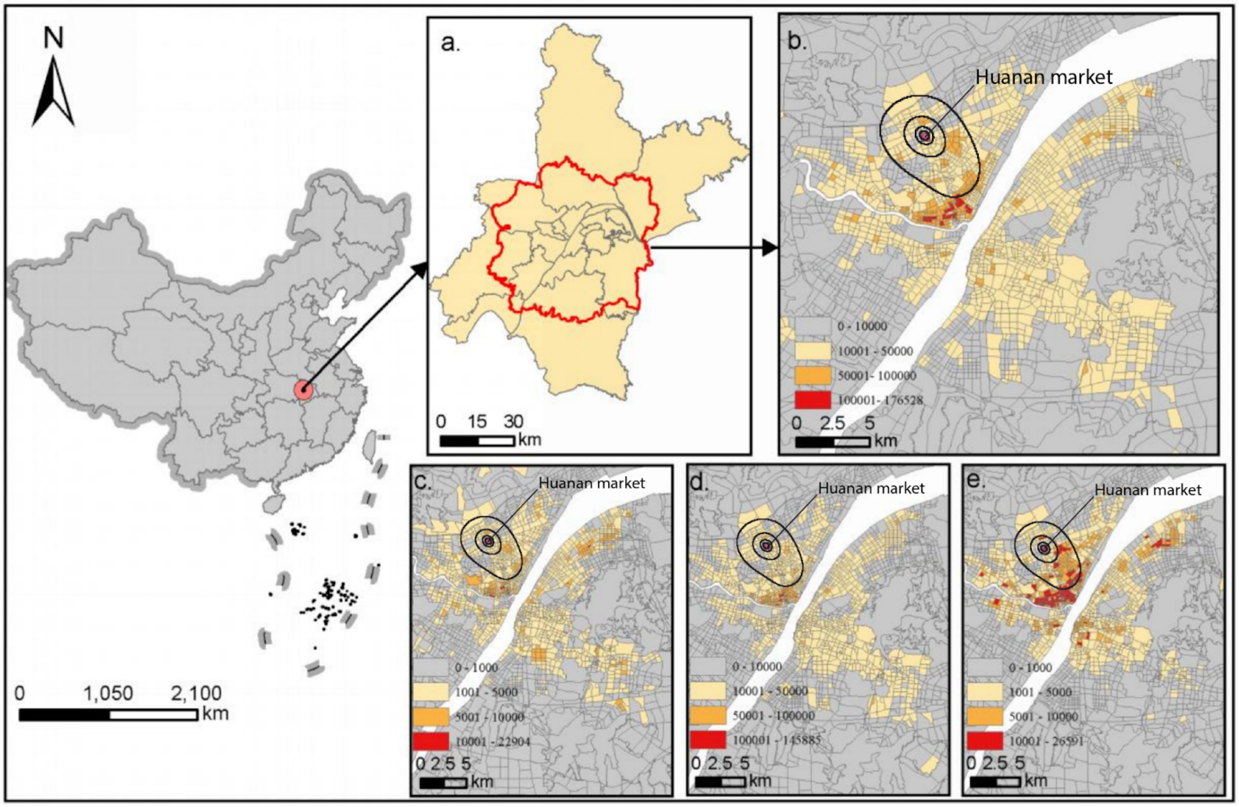

Figure S10. Comparison of highest density area of COVID-19 cases in December 2019 to density of overall population, child population, adult population and elderly population in Wuhan. This figure has been adapted slightly from Jia *et al*. (*63*) by the addition, in panels **b** through **e** of polygons representing the highest 50%, 25%, 10% and 1% KDE contours of December 2019 COVID-19 cases from the current study's Fig. 1C, plus the location of the Huanan market (pink square). As described in Jia et al. (*63*), panel **b** represents overall population density in central Wuhan, **c** children, **d** adult, and **e** elderly population density. Jia et al. retain copyright and the figure is made available under the CC BY 4.0 International License.

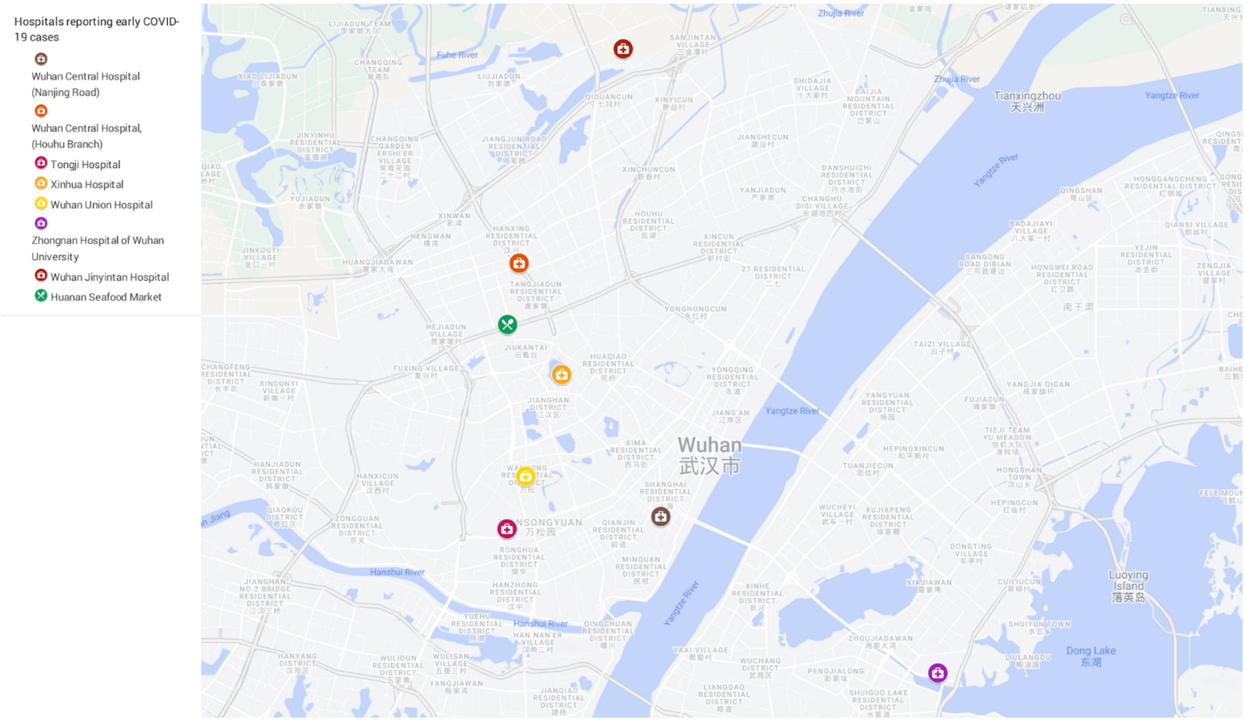

<u>Figure S11. The locations of key hospitals reporting early COVID-19 patients with respect to the Huanan market.</u> On the day that local public health authorities learned of an association of unexplained pneumonia cases with individuals connected to the Huanan market, they learned simultaneously about cases not just at hospitals near the market (e.g. Wuhan Central Hospital Houhu Branch and Xinhua Hospital) but also hospitals much further away (e.g. Tongji Hospital and Wuhan Union Hospital) (*5*). Importantly, major hospitals in districts many kilometers from the Huanan market, just like hospitals close to the market, found that their earliest cases (up to 3 January 2020) were dominated by individuals linked to the Huanan market, strong evidence that the virus was not cryptically widespread throughout the city but that cases were still, at that point, largely connected to the Huanan market (*5*).

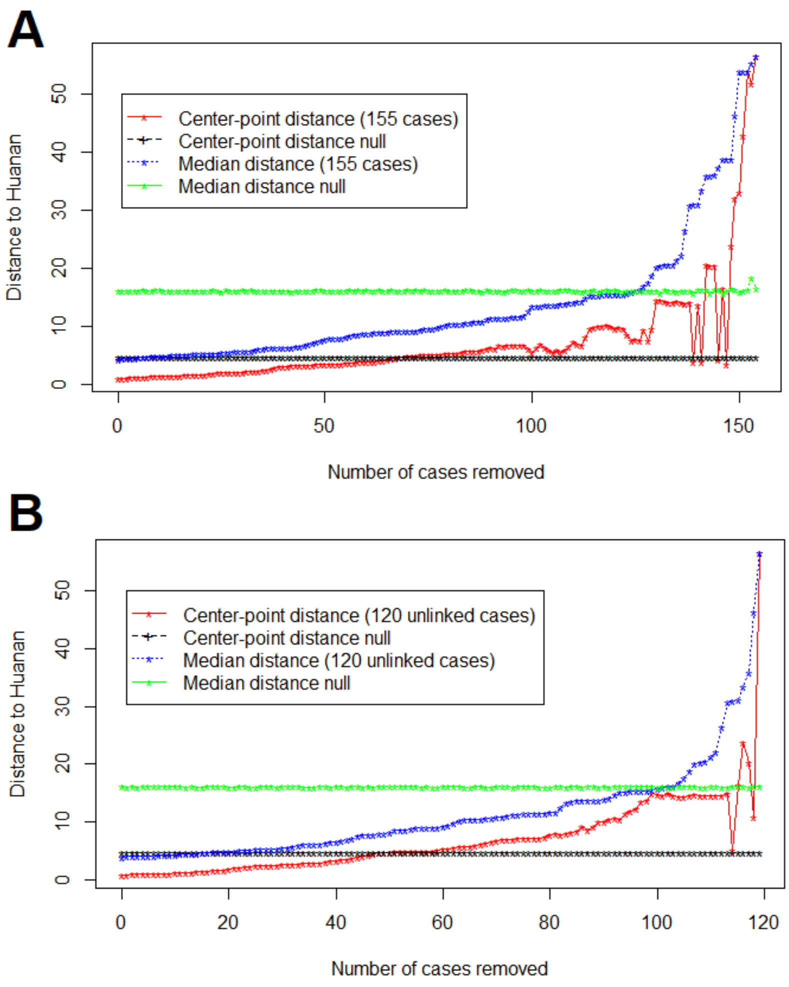

Figure S12. Effect of elimination of cases nearest the market on statistical results. To test the possibility of ascertainment bias, the nearest cases to the market were removed consecutively and the distance from center-point of the remaining cases to the Huanan market ("Center-point_distance"), and median distance of the remaining cases to the Huanan market ("Median_distance") were subsequently calculated. The x-axis indicates the number of cases closest to the market that have been removed from the dataset. The y-axis indicates the resulting median distance to the Huanan market and center-point distance to the market in kilometers. The center-point null plotted above is the distance between the Huanan market and the center-point of the 1,000,000 points sampled from the age-matched, weighted population density dataset. The median null distribution was generated by sampling the corresponding number of points shown in the x axis (155, 154, 153, and so on) from the population density dataset and calculating the median distance to the Huanan market. **A**: all cases. **B**: unlinked cases only.

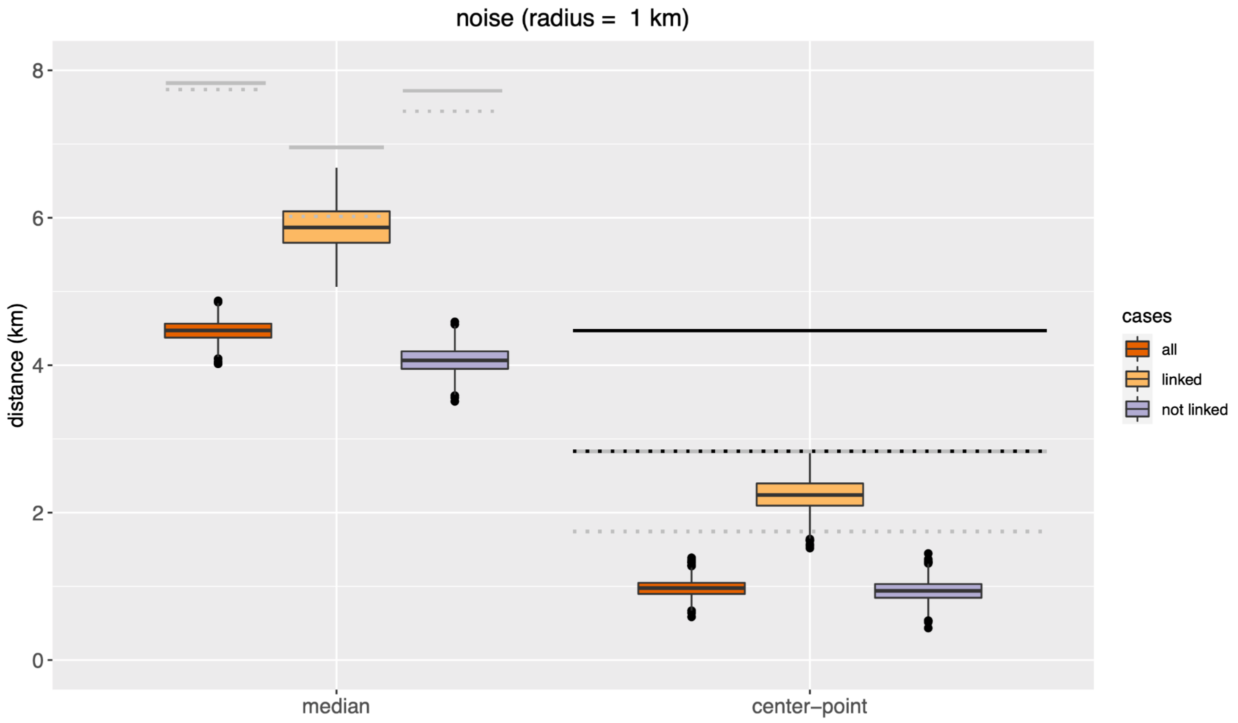

Figure S13. Robustness of the distance statistics (median and center-point distance) with respect to uncertainty in the extracted locations for all cases, linked cases and cases that are not linked to the Huanan market. The box plots summarize the distance statistics for 1000 replicates, with each replicate drawing locations uniformly at random from circles around the extracted location for each case with a radius of 1 km. Gray dotted and full gray lines indicate the 0.05 and 0.10 percentile value for the same statistics based on the Weibo data. Black dotted and full black lines indicate the 0.05 and 0.10 percentile value for the same statistics based on the worldpop.org data. If these lines are not shown for a distance statistic, they are above the upper limit of the y-axis scale. The boxes represent the interquartile ranges. The upper and lower whiskers represent the maximum and minimum value of the data that are within 1.5 times the interquartile range over the 75th percentile and under the 25th percentile respectively. Outlier values are considered any values over 1.5 times the interquartile range over the 75th percentile or any values under 1.5 times the interquartile range under the 25th percentile.

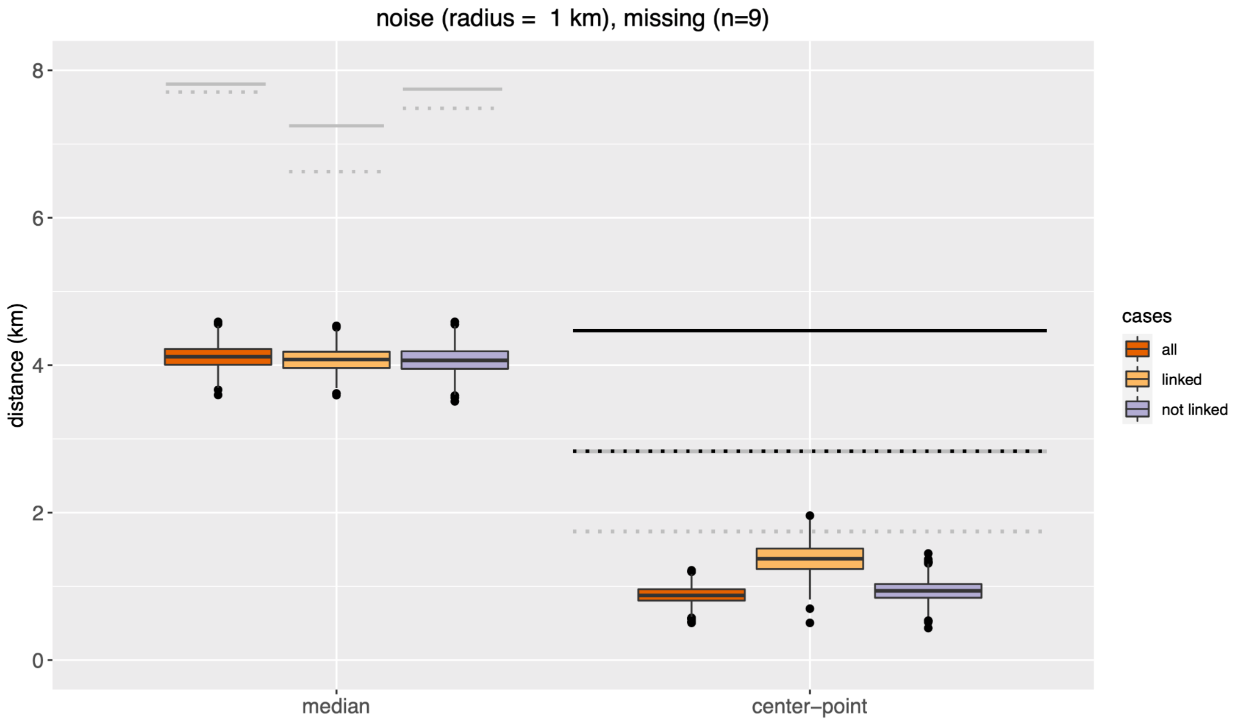

<u>Figure S14. Robustness of the distance statistics (median and center-point distance) with respect to uncertainty in the extracted locations and with respect to missing cases (*n*=9) for all cases, linked cases and cases that are not linked to the Huanan market.</u> The box plots summarize the distance statistics for 1000 replicates, with each replicate (i) drawing locations uniformly at random from circles around the extracted location for each case with a radius of 1 km and (ii) adding 9 locations drawn uniformly at random from the 25% kernel density estimate of the 155 December cases. Gray dotted and full gray lines indicate the 0.05 and 0.10 percentile value for the same statistics based on the Weibo data. Black dotted and full black lines indicate the 0.05 and 0.10 percentile value for the same statistics based on the worldpop.org data. If these lines are not shown for a distance statistic, they are above the upper limit of the y-axis scale. The boxes, whiskers and outliers follow the settings used for **fig. S9**.

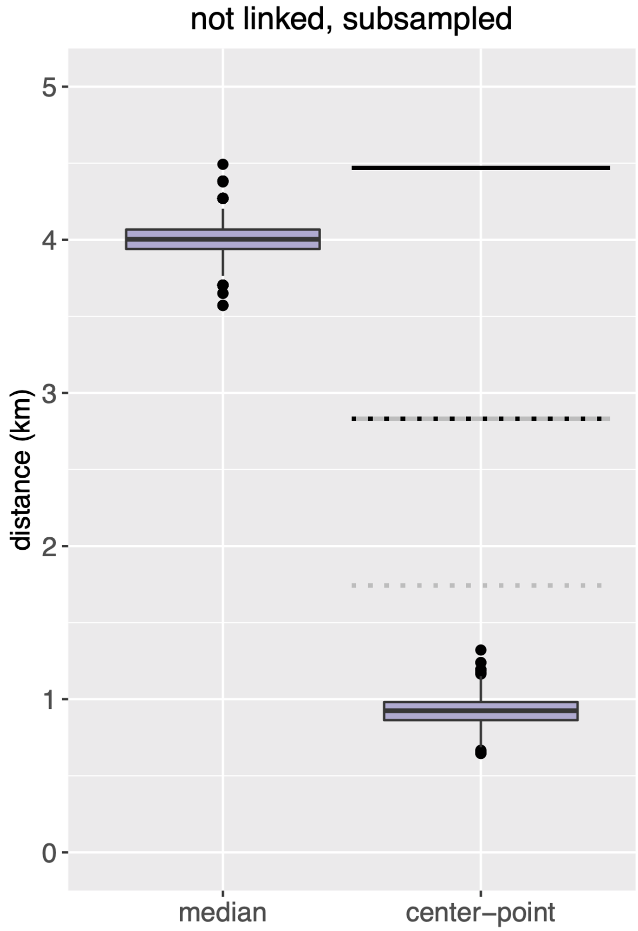

Figure S15. Robustness of the distance statistics (median and center-point distance) for the cases not linked to the Huanan market with respect to mis-assignment of some ‘linked’ cases as ‘not linked'. The box plots summarize the distance statistics for 1000 replicates, with each replicate subsampling 104 cases (out of 120) assigned as 'not linked'. Gray dotted and full gray lines indicate the 0.05 and 0.10 percentile value for the same statistics based on the Weibo data. Black dotted and full black lines indicate the 0.05 and 0.10 percentile value for the same statistics based on the worldpop.org data. If these lines are not shown for a distance statistic, they are above the upper limit of the y-axis scale. The boxes, whiskers and outliers follow the settings used for **fig. S9**.

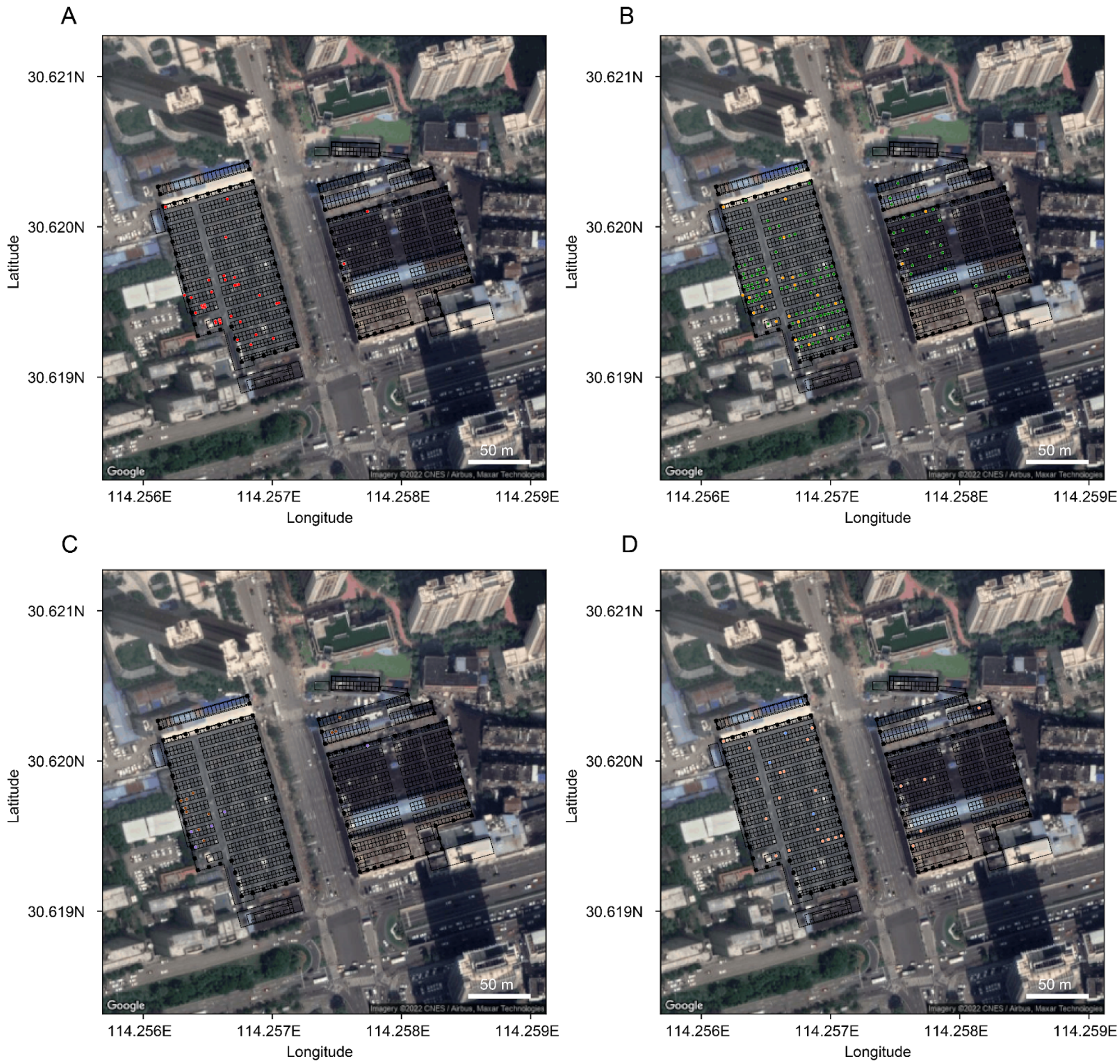

<u>Figure S16. Geospatial mapping of Huanan Seafood Market.</u> **A.** Locations of positive environmental samples (red), with jitter added to samples from stalls with multiple positive samples for visualization. **B**. Centroids of stalls with positive (orange) and negative (green) environmental samples. **C**. Centroids of known live mammal vendors (dark brown) and unknown meat vendors (lavender). **D**. Centroids of businesses with human cases up to and including December 20th (light blue), and after December 20th (salmon). Satellite imagery is obtained via the Google Maps Static API using Salem (*64*).

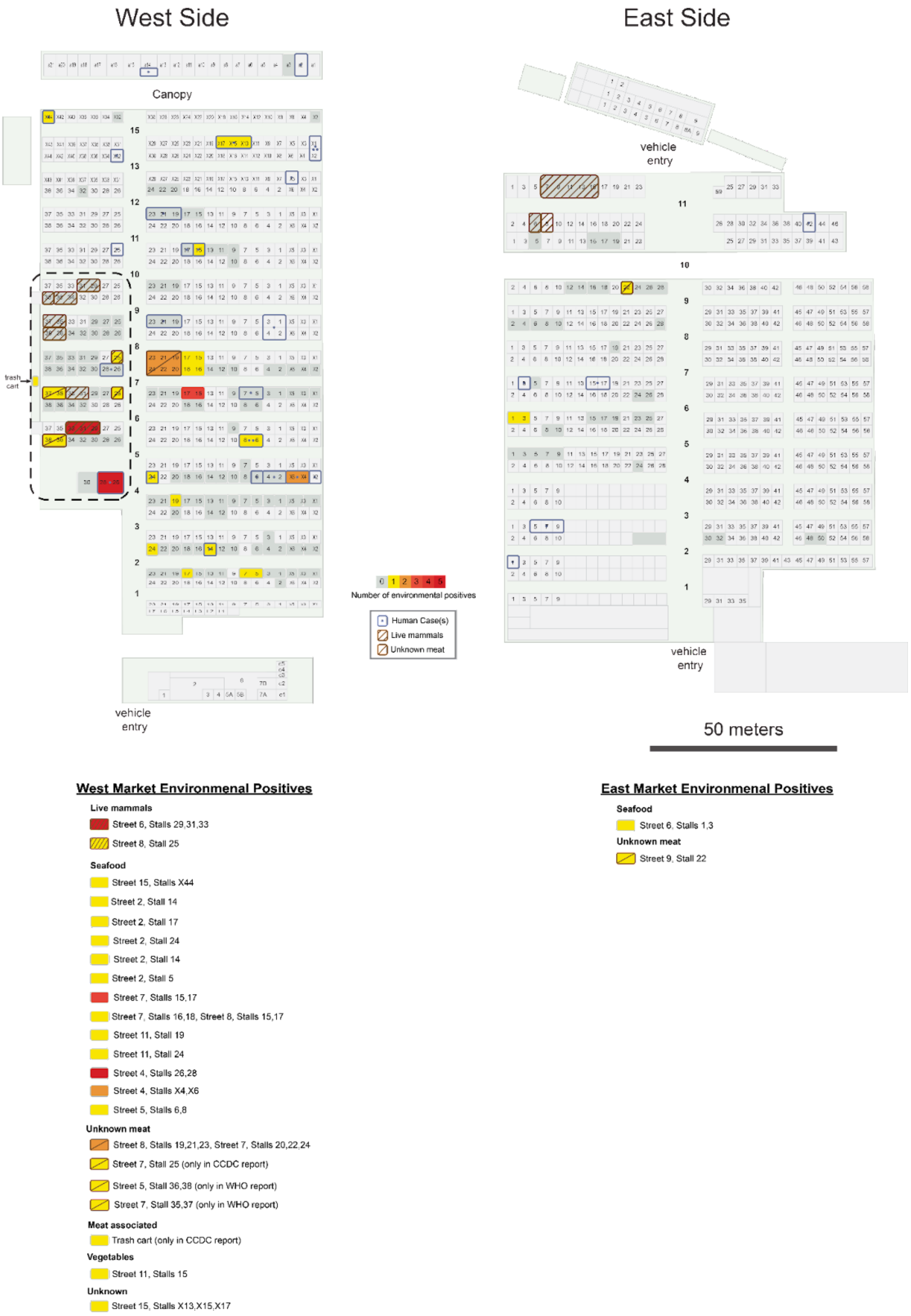

Figure S17. Huanan market environmental positives and stall addresses. Map of Huanan market including stall addresses and the type of products sold in each stall with a SARS-CoV-2 positive environmental sample.

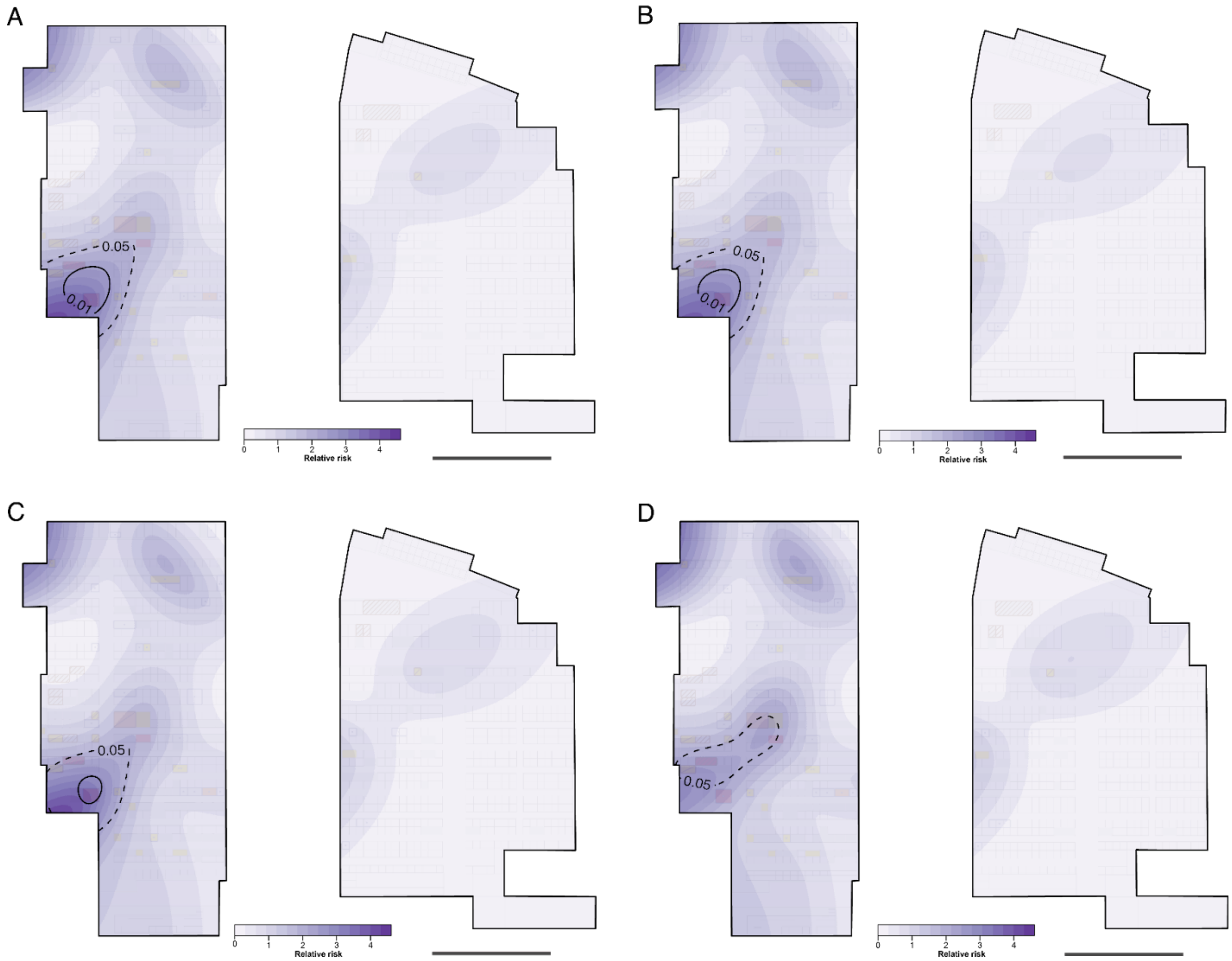

<u>Figure S18. Testing of possible sampling bias effects on observed relative risk in southwest corner of the Huanan market</u> **A-D** Relative risk analysis of positive environmental samples, **A.** for the case of 2x sampling from live mammal stalls **B.** 2x sampling from live mammal and unknown meat stalls **C.** for exclusion of samples with positive NGS but negative PCR, and **C.** Tolerance contours (p=0.05 denoted by dash, p=0.01 denoted by solid line) enclose regions with statistically significant elevation in density of positive environmental samples relative to the distribution of sampled stalls.

Supplementary Tables

Table S1. Distances from the Huanan market to the twenty nearest hotels

Hotel	Distance to market (m)*	Coordinates
Jinjiang Inn Wuhan Hankou Railway Station Branch	98 m**	30.6194 N, 114.2586 W
Non-Fan Premium Hotel (Wuhan Hankou Railway Station)	117 m	30.6194 N, 114.2588 W
100 Inns & Hotel (Wuhan Hankou Railway Station Branch)	126 m	30.6194 N, 114.2589 W
Dongfang Jianguo Hotel	402 m	30.6204 N, 114.2535 W
Lavande Hotels (Hankou Railway Station Grand Wuhan 1911 Branch)	183 m	30.6187 N, 114.2592 W
Sucha Hotel (Wuhan Hankou Railway Station)	308 m	30.6199 N, 114.2608 W
Tianya 1911 Hotel	279 m	30.6171 N, 114.2573 W
Building Lan Business Hotel	293 m	30.621 N, 114.255 W
Yibai Liangpin Hotel	422	30.6193 N, 114.2532 W
Lingbo Fashion Hotel	339	30.6207 N, 114.2543 W
Super 8 Hotel (Hankou Railway Station Branch)	422	30.6193 N, 114.2532 W
Huayi Selected Hotel	481	30.6192 N, 114.2526 W
Caiguang Hostel	491	30.6191 N, 114.2525 W
Kaixin Express Hotel	422	30.6193 N, 114.2532 W
Xana Hotel Hankou Railway Station	404	30.6182 N, 114.2537 W
Meirujia Hotel (Hankou Railway Station Branch)	336	30.6193 N, 114.2541 W
Hanting Hotel Hankou Railway Station Branch	364	30.6198 N, 114.2538 W
City Comfort Inn Chain Hankou Railway Station	407	30.619 N, 114.2534 W
Huangting Business Motel Hankou Railway Station	409	30.6189 N, 114.2534 W
7 days Inn Hankou Railway Station	402	30.6195 N, 114.2534 W

*Distance from hotel to Huanan market; median = 383 m.

**Distance to the closest edge of the market is closer for each hotel than to the central market point. For example, the Jinjiang Inn was 27 m from the edge of the east section of the market, but 98 m from the center point. To be conservative, we used distances from each hotel to the market's center point.

Table S2. Publications describing epidemiological characteristics of early COVID-19 patients.

December 2019	January 2019	Cohort	Patients	Huanan market (%)	Average age	ICU (%)	Case definition/diagnosis criteria
Huang 2020 Lancet (*6*)		Jinyintan Hospital	41	65.85%	49	31.70%	Clinical characteristics: fever and dry cough
Li 2020 NEJM (*62*)		Wuhan	47	55.30%	56		Clinical and epidemiological criteria
Chen 2020 Lancet (*39*)		Jinyintan Hospital	99	49.50%	55.5	23%	WHO interim guidance; *"Adult patients were admitted centrally to the hospital from the whole of Wuhan without selectivity… All the data of included cases have been shared with WHO"*
Wang 2020 JAMA (*65*)		Zhongnan Hospital	138	8%	56	26%	WHO interim guidance
WHO Data		Wuhan	174	33.50%	56		Clinical characteristics (n=64), lab confirmation (n=100)

Table S3. Case counts, weibo help seeker counts, total population, seropositivity, and case fatality rate per district in the city of Wuhan.

Neighborhood	Group	WHO cases (Dec)	Total cases (March)***	Total cases (March)****	Weibo help seekers*****	Population*	Cases per 100,000 (March) ***	Seropositivity (April)*	Seropositivity (April)**
Hanyang	North-urban	9	4691	4820	84	664,202.00	706.26	7.88	4.6
Jiangan	North-urban	28	6570	7030	111	962,695.00	682.46	11.12	6.5
Jianghan	North-urban	54	5199	5448	72	729,704.00	712.48	7.83	5.2
Qiaokou	North-urban	12	6863	7143	110	868,702.00	790.03	13.08	11.2
Hongshan	South-urban	10	6990	7403	84	1,677,298.00	416.74	5.62	2.9
Qingshan	South-urban	4	2788	2979	63	528,894.00	527.14	5.54	3.8
Wuchang	South-urban	6	7484	7726	133	1,282,800.00	583.41	5.15	3.7
Dongxihu	North-suburb	12	2465	2544	40	584,803.00	421.51	5.07	2.3
Huangpi	North-suburb	13	2126	2204	10	1,011,897.00	210.10	1.62	1
Xinzhou	North-suburb	1	1073	1116	2	910,595.00	117.84	2.35	0.7
Caidian	South-suburb	2	1417	1422	2	469,490.00	301.82	1.49	0.9
Hannan	South-suburb	2	1094	1146	1	427,916.00	255.66	0.73	0.5
Jiangxia	South-suburb	0	1213	1159	25	962,000.00	126.09	5.07	0.4

* Data from (*41*)
** Data from (*40*)
*** Data from (*66*)
**** Data from (*67*)
***** Data from (*26*)

Table S4. Results when corrected for multiple hypothesis testing using the Benjamini-Hochberg procedure

Metric	Group	Sample size (n)	Distance from Huanan market (km)	Null/control distribution	P-value	P-adjusted (BH)
median distance	all cases	155	4.28	weibo null	p<0.001	p<0.003
median distance	not linked	120	4.00	weibo null	p<0.001	p<0.003
median distance	linked	35	5.74	weibo null	0.031	0.041
median distance	B	11	8.30	weibo null	0.458	0.458
median distance	not linked A (1 + hotel guest)	2	2.31	weibo null	0.009	0.016
median distance	not linked A (1)	1	2.31	weibo null	0.068	0.073
median distance	confirmed	65	2.91	weibo null	p<0.001	p<0.003
median distance	clinically diagnosed	79	4.66	weibo null	p<0.001	p<0.003
median distance	all cases	155	4.28	worldpop age null	p<0.001	p<0.003
median distance	not linked	120	4.00	worldpop age null	p<0.001	p<0.003
median distance	linked	35	5.74	worldpop age null	p<0.001	p<0.003
median distance	B	11	8.30	worldpop age null	0.017	0.026
median distance	not linked A (1 + hotel guest)	2	2.31	worldpop age null	p<0.001	p<0.003
median distance	not linked A (1)	1	2.31	worldpop age null	0.034	0.041
median distance	confirmed	65	2.91	worldpop age null	p<0.001	p<0.003
median distance	clinically diagnosed	79	4.66	worldpop age null	p<0.001	p<0.003
center-point distance	all cases	155	1.02	weibo null	0.015	0.025
center-point distance	not linked	120	0.91	weibo null	0.005	0.011
center-point distance	linked	35	2.28	weibo null	0.075	0.078
center-point distance	B	11	1.95	weibo null	0.064	0.072
center-point distance	confirmed	65	0.32	weibo null	0.002	0.005
center-point distance	clinically diagnosed	79	1.80	weibo null	0.051	0.060
center-point distance	all cases	155	1.02	worldpop age null	0.008	0.015
center-point distance	not linked	120	0.91	worldpop age null	0.006	0.012
center-point distance	linked	35	2.28	worldpop age null	0.034	0.041
center-point distance	B	11	1.95	worldpop age null	0.026	0.036
center-point distance	confirmed	65	0.32	worldpop age null	0.001	0.003
center-point distance	clinically diagnosed	79	1.80	worldpop age null	0.022	0.032

Table S5. Live mammals traded at the Huanan market, October - December, 2019

Species	Family/Order	Susceptibility	Observed at Huanan market, October 2019	Observed at Huanan market, November 2019	Observed at Huanan market, December 2019*
Raccoon dog *(Nyctereutes procyonoides)***	Canidae/Carnivora	Live*** (*9*)(*68*)(*69*)	Yes	Yes	Yes
Amur hedgehog *(Erinaceus amurensis)*	Erinaceidae/Eulipotyphla		Yes	Yes	
Hog badger *(Arctonyx albogularis)*	Mustelidae/Carnivora	ACE2**** (*69*)	Yes	Yes	
Asian badger *(Meles leucurus)*	Mustelidae/Carnivora	See raccoon dog, mink, etc.	Yes	Yes	
Chinese hare *(Lepus sinensis)*	Leporidae/Lagomorpha	Rabbit :(*70*)	Yes	Yes	
Chinese bamboo rat *(Rhizomys sinensis)*	Spalacidae/Rodentia	ACE2 (*71*)	Yes	Yes	
Malayan porcupine *(Hystrix brachyura)*	Hystricidae/Rodentia	See bamboo rat	Yes	Yes	Yes
Chinese muntjac *(Muntiacus reevesi)*	Cervidae/Artiodactyla	White-tailed deer: Live (*72*)	Yes	Yes	Yes
Marmot *(Marmota himalayana)*	Sciuridae/Rodentia	See bamboo rat	Yes	Yes	Yes
Red fox *(Vulpes vulpes)*	Canidae/Carnivora	Live (*73*), Serology***** (*74*)	Yes	Yes	Yes
Siberian weasel *(Mustela sibirica)*	Mustelidae/Carnivora	See raccoon dog, mink, etc.	No	No	
Pallas's squirrel *(Callosciurus erythraeus)*	Sciuridae/Rodentia	See bamboo rat	No	No	
Masked palm civet *(Paguma larvata)*	Viverridae/Carnivora	ACE2 (*69*)(*71*)(*75*)	No	No	
Coypu *(Myocastor coypus)*	Echimyidae/Rodentia	See bamboo rat	No	No	
Mink *(Neovison vison)***	Mustelidae/Carnivora	Live (*76*)(*46*)(*77*)(*78*)(*79*)	No	No	
Red squirrel *(Sciurus vulgaris)*	Sciuridae/Rodentia	See bamboo rat	No	No	
Wild boar *(Sus scrofa)*	Suidae/Artiodactyla	Serology (*74*), ACE2 (*75*)(*80*)	No	No	
Complex-toothed flying squirrel *(Trogopterus xanthipes)*	Sciuridae/Rodentia	See bamboo rat	No	No	

* From photographic evidence from 3 December, 2019 (*81*).
** Established transmissibility, not just susceptibility.
*** Demonstrated susceptibility in live animals
**** Based on serological findings in wild populations
***** Based on predicted or demonstrated ACE2 binding.

Table S6. Environmental samples from the Huanan market.

Code	Area	Street	Stall	Store type	Known cases in stall	Wildlife area?	Illegal Hedgehog sales 2019	Type	RT-PCR	N gene seq.	S gene seq.	NGS	GISAID Sequence	Length	Coverage	GISAID Culture sequence	Virus Lineage
E61	East	6	1,3	Seafood				Ground surface	POS								?
E48	East	9	22	Meat, unknown				Blood from ground surface, in front of door	POS	>99%							?
D32	West	15	X15		Yes			Wagon surface	POS								?
B17	West		X44	Seafood	Yes			Scale	POS								?
G93	West	8	19, 21, 23	Meat, unknown				Dirty water	POS								?
F54	West	2	14	Seafood	Yes			Ground surface	POS	>99%		>99%	EPI_ISL_408512	25,342	2.4		B
F33	West		17	Seafood				Sluice gate	POS								?
F46	West		24	Seafood				Ground surface	POS	>99%	>99%						?
E7	West		5,7	Seafood				Foam dispenser	POS	>99%							?
A20	West	7	15, 17	Seafood				Gloves	POS	>99%	>99%						A
A18	West			Seafood				Shoe bottoms	POS	>99%							?
A2	West			Seafood				Ground surface	POS	>99%							?
A63	West		16, 18	Seafood				Ground surface	POS		>99%						?
A61	West		20, 22, 24	Meat, unknown	Adjacent			Ground surface	POS								?
A55	West		25	Meat, unknown†††	Adjacent	Yes		Ground surface	POS	>99%							?
A33	West		West end	Meat associated		Yes		Trash cart	POS								?
F13	West	11	15	Vegetable	Yes			Wall surface	POS	>99%	>99%	>99%	EPI_ISL_408511	28,557	25.6	EPI_ISL_408514<br>EPI_ISL_408515	B
A101	West	4	19	Seafood				Door surface	POS								?
A96	West		24	Seafood	Yes			Ground surface	POS								?
A14	West		26	Seafood	Yes	Yes		Fish packaging surface	POS								?
A15	West			Seafood	Yes	Yes		Door surface	POS								?
A90	West			Seafood	Yes	Yes		Ground surface	POS								?
A87	West		28	Seafood	Yes	Yes		Door surface	POS								?
A88	West			Seafood	Yes	Yes		Ground surface	POS								?
F98	West		X6, X4	Seafood	Yes			Ground surface	POS	>99%							?
F100	West			Seafood	Yes			Ground surface	POS	>99%							?
B5	West	5	6, 8	Seafood	Yes			Ground surface	POS	>99%		>99%	EPI_ISL_408513	1,065	2.4		B
Q37	West	8	25	Domesticated wildlife††		Yes	Yes†	Interior surface of freezer	NEG			>99%					?
Q61	West	6	29	Domesticated wildlife		Yes	Yes†	Cart 1	NEG			>99%					?
Q64	West		29	Domesticated wildlife		Yes	Yes†	Cart 2	POS			>99%					?
Q68	West		29	Domesticated wildlife		Yes	Yes†	Ground surface	POS			>99%					?
Q69	West		29	Domesticated wildlife		Yes	Yes†	Hair/feather removal machine (epilator)	POS			>99%					?
Q70	West		29	Domesticated wildlife		Yes	Yes†	Metal cage in inner room	NEG			>99%					?
WHO	West	5	36,38	Meat, unknown†††		Yes		Unknown	POS								?

WHO	West	7	35,37	Meat, unknown		Yes		Unknown	POS									?

Methodology: Business names were obtained from the TianYanCha.com business directory (see Table S8) based on addresses. In many cases, business names were further confirmed through photographic evidence of stalls. Two stalls were identified as involved in domesticated wildlife sales from an official local forestry bureau fine for their registered owners for illegal hedgehog sales in summer 2019 (*36*).

† The "Operators" of these businesses listed in TianYanCha.com business directory were fined for illegal hedgehog sales in summer 2019, in an official government report (*36*)

†† Reported in WHO mission report Appendix F, Table 3, contains "game" in the business name, lists game retail as a service in the business directory, and was fined for illegal hedgehog sales in 2019 (see †) (*7*).

††† Store selling meat products inferred from photographic evidence.

Table S7. Key numbers regarding environmental sampling at the Huanan market.

Key numbers		Source
585	Total number of Environmental Samples, CCDC	CCDC page 1
26	Number of positive stalls, CCDC	From CCDC table
33	Total number of Positive samples, CCDC	CCDC page 1
31	Number of positive samples from western section, CCDC	CCDC page 2
718	Total number of Environmental Samples, WHO	WHO page 95
678	Total number of stalls in the market, WHO	WHO page 96
134	Number of stalls sampled in the market, WHO	WHO page 96
21	Number of positive stalls, WHO	WHO Annex E4, 4.5 Market environment
113	Number of negative stalls, WHO	WHO Annex E4, 4.5 Market environment
40	Number of positive samples, WHO	WHO page 95
69	Number of positive samples including sewage and warehouses, WHO	WHO page 95
174	All December 2019 cases	WHO page 43 (Fig 22)
164	All Wuhan December 2019 cases	WHO page 43
55	Cases connected to Huanan Market	WHO, Annex E4 "Table 3. General information of cases"
30	Huanan Market Vendor cases	WHO, Annex E4 "Table 3. General information of cases"

Table S8. Public business registry of specific stalls with SARS-CoV-2 positive samples at the Huanan market.

Street	Address	Tianyancha link*
8	25	https://www.tianyancha.com/company/2336478151
5	"Corridor" (36,38)	https://www.tianyancha.com/company/2334674880
7	25,27	https://www.tianyancha.com/company/2339714863
7,8	19,20,21,22,23,24	https://www.tianyancha.com/company/2336843423
6	29,31,32,33	https://www.tianyancha.com/company/2336320983
2	14	https://www.tianyancha.com/company/1434754801

*Screenshots available in data S2.

Table S9. Binomial GLM results for possible values of total samples (N) per stall.

Using distance to a live mammal vendor						
**Samples per stall**	**N=5**	**N=6**	**N=7**	**N=8**	**N=9**	**N=10**
Estimated coefficient (wildlife stall distance)	-0.0290	-0.0287	-0.0285	-0.0283	-0.0282	-0.0281
Detection odds % decrease/m (wildlife stall distance)	2.86	2.83	2.81	2.79	2.78	2.77
P-value (wildlife stall distance)	0.00419	0.00434	0.00445	0.00454	0.00461	0.00466
Estimated coefficient (case distance)	-0.0692	-0.0684	-0.0679	-0.0675	-0.0672	-0.0670
Detection odds % decrease/m (case distance)	6.68	6.62	6.57	6.53	6.50	6.48
P-value (case distance)	0.01323	0.01355	0.01379	0.01396	0.01410	0.01421
**Using distance to a live mammal vendor or an unknown meat vendor**						
**Samples per stall**	**N=5**	**N=6**	**N=7**	**N=8**	**N=9**	**N=10**
Estimated coefficient (wildlife stall distance)	-0.0354	-0.0350	-0.0347	-0.0345	-0.0343	-0.0342
Detection odds % decrease/m (wildlife stall distance)	3.48	3.44	3.41	3.39	3.38	3.36
P-value (wildlife stall distance)	0.00242	0.00252	0.00258	0.00264	0.00268	0.00271
Estimated coefficient (case distance)	-0.0764	-0.0756	-0.0750	-0.0745	-0.0742	-0.0739
Detection odds % decrease/m (case distance)	7.36	7.28	7.22	7.18	7.15	7.12
P-value (case distance)	0.00752	0.00772	0.00787	0.00798	0.00806	0.00813

Table S10. Binomial GLM results under various possible sources of sampling bias.

	2x Sampling of live mammal stall, using distance to a live mammal vendor						2x Sampling of live mammal and unknown meat stalls, using distance to live mammal or unknown meat vendors					
**Samples per stall**	**N=5,10**	**N=6,12**	**N=7,14**	**N=8,16**	**N=9,18**	**N=10,20**	**N=5,10**	**N=6,12**	**N=7,14**	**N=8,16**	**N=9,18**	**N=10,20**
Estimated coefficient (wildlife stall distance)	-0.0231	-0.0228	-0.0227	-0.0225	-0.0225	-0.0224	-0.264	-0.0261	-0.0259	-0.0258	-0.0257	-0.0256
Estimated coefficient (case distance)	-0.0745	-0.0738	-0.0733	-0.0729	-0.0726	-0.0724	-0.0820	-0.0812	-0.0807	-0.0803	-0.0800	-0.0797
Detection odds % decrease/m (wildlife stall distance)	2.28	2.26	2.24	2.23	2.22	2.21	2.6	2.58	2.56	2.55	2.54	2.53
Detection odds % decrease/m (case distance)	7.18	7.11	7.07	7.03	7.01	6.99	7.87	7.8	7.75	7.71	7.68	7.66
P-value (wildlife stall distance)	0.0177	0.0182	0.0185	0.0188	0.019	0.0191	0.0167	0.0171	0.0174	0.0176	0.0178	0.0179
P-value (case distance)	0.0069	0.0071	0.0072	0.0073	0.0074	0.0074	0.0039	0.004	0.0041	0.0041	0.0042	0.0042
	**Only including PCR positive samples**						**Only including PCR positive samples, using distance to live mammal or unknown meat vendors**					
**Samples per stall**	**N=5**	**N=6**	**N=7**	**N=8**	**N=9**	**N=10**	**N=5**	**N=6**	**N=7**	**N=8**	**N=9**	**N=10**
Estimated coefficient (wildlife stall distance)	-0.0242	-0.0240	-0.0238	-0.0237	-0.0236	-0.0236	-0.0309	-0.0305	-0.0303	-0.0302	-0.0300	-0.0300
Estimated coefficient (case distance)	-0.0750	-0.0743	-0.0738	-0.0734	-0.0731	-0.0729	-0.0823	-0.0815	-0.0809	-0.0805	-0.0801	-0.0799
Detection odds % decrease/m (wildlife stall distance)	2.4	2.37	2.36	2.34	2.33	2.33	3.04	3.01	2.99	2.97	2.96	2.95
Detection odds % decrease/m (case distance)	7.22	7.16	7.11	7.08	7.05	7.03	7.9	7.82	7.77	7.73	7.7	7.68
P-value (wildlife stall distance)	0.0216	0.0221	0.0224	0.0227	0.0229	0.0231	0.0106	0.0109	0.0111	0.0113	0.0114	0.0115
P-value (case distance)	0.0119	0.0122	0.0124	0.0125	0.0126	0.0127	0.0071	0.0073	0.0074	0.0075	0.0075	0.0076
	**Excluding seafood stall (Street 4, Stalls 26,28)**						**Excluding seafood stall, using distance to live mammal or unknown meat vendors**					
**Samples per stall**	**N=5**	**N=6**	**N=7**	**N=8**	**N=9**	**N=10**	**N=5**	**N=6**	**N=7**	**N=8**	**N=9**	**N=10**
Estimated coefficient (wildlife stall distance)	-0.0222	-0.0221	-0.0219	-0.0218	-0.0218	-0.0217	-0.0290	-0.0288	-0.0286	-0.0285	-0.0284	-0.0283
Estimated coefficient (case distance)	-0.0369	-0.0366	-0.0364	-0.0362	-0.0361	-0.0360	-0.0434	-0.0430	-0.0428	-0.0426	-0.0425	-0.0423
Detection odds % decrease/m (wildlife stall distance)	2.2	2.18	2.17	2.16	2.15	2.15	2.86	2.84	2.82	2.81	2.8	2.79
Detection odds % decrease/m (case distance)	3.62	3.59	3.57	3.56	3.55	3.54	4.25	4.21	4.19	4.17	4.16	4.15
P-value (wildlife stall distance)	0.0307	0.0312	0.0317	0.032	0.0322	0.0324	0.0144	0.0148	0.015	0.0152	0.0153	0.0154
P-value (case distance)	0.16	0.1615	0.1626	0.1633	0.1639	0.1644	0.1104	0.1116	0.1124	0.1131	0.1136	0.1139

Table S11. Human SARS-CoV-2-positive cases at the Huanan market.

Source	Location	Street	Stalls	#	Notes	Date (before/by)
Media	West	2	14	1	10 December 2019 onset (*5*); missing from WHO report (*5*).	12-10
WHO	West	12	19,21,23	1		12-13
WHO	West	5	6,8	1		12-20
WHO	West	5	6,8	1		12-27
WHO	West	7	5,7	1		12-20
WHO	West	13	X2	2	also stall 15 X1	12-27
WHO	West	13	X2	1	also stall 15 X1	12-31
WHO	West	15	X13,X15,X17	1		12-20
WHO	West		A2	1		12-20
WHO	West	4	26,28	1		12-31
WHO	West	4	24	1		12-31
WHO	West	4	X2	1		12-31
WHO	West	4	X4,X6	1		12-31
WHO	West	4	2,4	1		12-31
WHO	West	4	6	1		12-31
WHO	West	7	26,28	1		12-31
WHO	West	9	12,21,23	1		12-31
WHO	West	8	2,4	1	also stall 9 1,3	12-31
WHO	West	11	25	1		12-31
WHO	West	11	15,17	1		12-31
WHO	West	13	X32	1		12-31
WHO	West	13	X5	1		12-31
WHO	West		A15	1		12-31
WHO	West	15	X44	1		12-31
WHO	East	2	1	1		12-31
WHO	East	3	5,7,9	1		12-31
WHO	East	7	3	1		12-31
WHO	East	7	19,21	1		12-31
WHO	East	11	42	1		12-31

Table S12. Environmentally sampled, SARS-CoV-2-negative stalls in the Huanan market.

Section of market	Street	Stall
West		A3
West	15	X2
West	15	X32
West	12	20-24
West	12	32
West	12	15-17
West	12	19-23
West	11	17
West	11	11-13
West	11	9
West	10	19-23
West	10	29-31
West	9	34-36
West	9	38
West	9	19-23
West	9	27-29
West	9	25
West	9	35-37
West	8	26-28
West	8	30
West	8	32-34
West	8	36-38
West	8	29-31
West	8	33
West	8	35
West	8	37
West	7	X2
West	7	X4-X6
West	7	2-4
West	7	10
West	7	12-14
West	7	26-28
West	7	30-32
West	7	34
West	7	36-38
West	7	31-33
West	7	29
West	7	27

West	7	25
West	7	23
West	7	19-21
West	7	9
West	7	5-7
West	7	1-3
West	7	X3-X5
West	7	X1
West	6	38
West	6	36
West	6	34
West	6	32
West	6	16-18
West	6	6-8
West	6	9
West	5	26-34
West	5	7
West	4	30
West	4	20
West	4	16-18
West	4	12-14
West	4	10
West	4	8
West	4	6
West	4	2-4
West	4	X4-X6
West	4	X2
West	4	21-23
West	4	15-17
West	4	11-13
West	4	9
West	4	7
West	4	5
West	4	3
West	4	1
West	4	X3-X5
West	4	X1
West	3	20
West	2	22
West	2	20

West	2	18
West	2	16
West	2	10-12
West	2	6
West	2	2-4
West	2	X4-X6
West	2	X2
West	2	19-23
West	2	15
West	2	11-13
West	2	1-3
West	2	X5
West	2	X1-X3
West	1	2
East	11	7-15
East	11	6
East	10	17-21
East	10	5
East	9	24-26
East	9	14-16
East	9	8-12
East	8	2-4
East	8	6-10
East	8	28
East	8	19
East	7	5
East	6	24-26
East	6	23-25
East	6	15-19
East	5	8-10
East	5	1-9
East	4	24
East	2	24-28
East	2	30-32
East	2	48-50

References and Notes

1. Sina Finance, "Wuhan pneumonia of unknown cause cases isolated, test results to be announced ASAP" (Sina Finance, 2019); https://finance.sina.cn/2019-12-31/detail-iihnzahk1074832.d.html?from=wap.

2. Wuhan Municipal Health Commission, "Wuhan Municipal Health Commission's briefing on the current situation of pneumonia in our city" (Wuham Municipal Health Commission, 2019); https://web.archive.org/web/20200131202951/http:/wjw.wuhan.gov.cn/front/web/showDetail/2019123108989.

3. World Health Organization, "COVID-19 – China" (WHO, 2020); https://www.who.int/emergencies/disease-outbreak-news/item/2020-DON229.

4. The Novel Coronavirus Pneumonia Emergency Response Epidemiology Team, The epidemiological characteristics of an outbreak of 2019 novel coronavirus diseases (COVID-19) – China, 2020. *China CDC Wkly* **2**, 113–122 (2020). doi:10.46234/ccdcw2020.032 Medline

5. M. Worobey, Dissecting the early COVID-19 cases in Wuhan. *Science* **374**, 1202–1204 (2021). doi:10.1126/science.abm4454 Medline

6. C. Huang, Y. Wang, X. Li, L. Ren, J. Zhao, Y. Hu, L. Zhang, G. Fan, J. Xu, X. Gu, Z. Cheng, T. Yu, J. Xia, Y. Wei, W. Wu, X. Xie, W. Yin, H. Li, M. Liu, Y. Xiao, H. Gao, L. Guo, J. Xie, G. Wang, R. Jiang, Z. Gao, Q. Jin, J. Wang, B. Cao, Clinical features of patients infected with 2019 novel coronavirus in Wuhan, China. *Lancet* **395**, 497–506 (2020). doi:10.1016/S0140-6736(20)30183-5 Medline

7. World Health Organization, "WHO-convened global study of origins of SARS-CoV-2: China Part" (WHO, 2021); https://www.who.int/publications/i/item/who-convened-global-study-of-origins-of-sars-cov-2-china-part.

8. X. Xiao, C. Newman, C. D. Buesching, D. W. Macdonald, Z.-M. Zhou, Animal sales from Wuhan wet markets immediately prior to the COVID-19 pandemic. *Sci. Rep.* **11**, 11898 (2021). doi:10.1038/s41598-021-91470-2 Medline

9. C. M. Freuling, A. Breithaupt, T. Müller, J. Sehl, A. Balkema-Buschmann, M. Rissmann, A. Klein, C. Wylezich, D. Höper, K. Wernike, A. Aebischer, D. Hoffmann, V. Friedrichs, A. Dorhoi, M. H. Groschup, M. Beer, T. C. Mettenleiter, Susceptibility of raccoon dogs for experimental SARS-CoV-2 infection. *Emerg. Infect. Dis.* **26**, 2982–2985 (2020). doi:10.3201/eid2612.203733 Medline

10. W. K. Jo, E. F. de Oliveira-Filho, A. Rasche, A. D. Greenwood, K. Osterrieder, J. F. Drexler, Potential zoonotic sources of SARS-CoV-2 infections. *Transbound. Emerg. Dis.* **68**, 1824–1834 (2021). doi:10.1111/tbed.13872 Medline

11. I. R. Fischhoff, A. A. Castellanos, J. P. G. L. M. Rodrigues, A. Varsani, B. A. Han, Predicting the zoonotic capacity of mammals to transmit SARS-CoV-2. *Proc. Biol. Sci.* **288**, 20211651 (2021). doi:10.1098/rspb.2021.1651 Medline

12. W. Guizhen, “Chinese CDC disease control report” (see data S1).

13. Xinhua News, “Good news! Phased progress made in tracing the origin of the coronavirus” (Xinhua News, 2020); http://www.xinhuanet.com/politics/2020-01/26/c_1125503792.htm.

14. Beijing News, “Huanan Seafood Market in the pneumonia of unexplained incident” (Beijing News, 2020); http://www.bjnews.com.cn/feature/2020/01/02/669054.html.

15. Chinese Center for Disease Control and Prevention, “Chinese Center for Disease Control and Prevention detects large quantity of novel coronavirus in Wuhan Huanan Seafood Market” (Chinese CDC, 2020); https://www.chinacdc.cn/yw_9324/202001/t20200127_211469.html.

16. Yicai Global, “China detects large quantity of novel coronavirus at Wuhan Seafood Market” (Yicai Global, 2020); https://www.yicaiglobal.com/opinion/yicai.global/china-detects-large-quantity-of-novel-coronavirus-at-wuhan-seafood-market.

17. Chinese Center for Disease Control and Prevention, “China CDC calls on the public to protect themselves” (Chinese CDC, 2020); https://www.chinacdc.cn/yw_9324/202001/t20200128_211498.html.

18. Chinese Center for Disease Control and Prevention, “On the front line, disease control warriors race against the new coronavirus” (Chinese CDC, 2020); https://www.chinacdc.cn/yw_9324/202002/t20200201_212137.html.

19. Xinhua News, “China detects large quantity of novel coronavirus at Wuhan seafood market” (Xinhua News, 2020); https://web.archive.org/web/20200126230041/http://www.xinhuanet.com/english/2020-01/27/c_138735677.htm.

20. Z. Shi, Z. Hu, A review of studies on animal reservoirs of the SARS coronavirus. *Virus Res.* **133**, 74–87 (2008). doi:10.1016/j.virusres.2007.03.012 Medline

21. W. B. Karesh, R. A. Cook, E. L. Bennett, J. Newcomb, Wildlife trade and global disease emergence. *Emerg. Infect. Dis.* **11**, 1000–1002 (2005). doi:10.3201/eid1107.050194 Medline

22. N. D. Wolfe, P. Daszak, A. M. Kilpatrick, D. S. Burke, Bushmeat hunting, deforestation, and prediction of zoonoses emergence. *Emerg. Infect. Dis.* **11**, 1822–1827 (2005). doi:10.3201/eid1112.040789 Medline

23. C. K. Johnson, P. L. Hitchens, P. S. Pandit, J. Rushmore, T. S. Evans, C. C. W. Young, M. M. Doyle, Global shifts in mammalian population trends reveal key predictors of virus spillover risk. *Proc. Biol. Sci.* **287**, 20192736 (2020). doi:10.1098/rspb.2019.2736 Medline

24. G. Gao, W. Liu, P. Liu, W. Lei, Z. Jia, X. He, L.-L. Liu, W. Shi, Y. Tan, S. Zou, X. Zhao, G. Wong, J. Wang, F. Wang, G. Wang, K. Qin, R. Gao, J. Zhang, M. Li, W. Xiao, Y. Guo, Z. Xu, Y. Zhao, J. Song, J. Zhang, W. Zhen, W. Zhou, B. Ye, J. Song, M. Yang, W. Zhou, Y. Bi, K. Cai, D. Wang, W. Tan, J. Han, W. Xu, G. Wu, “Surveillance of SARS-CoV-2 in the environment and animal samples of the Huanan Seafood Market” [Preprint] (Research Square, 2022); https://www.researchsquare.com/article/rs-1370392/v1.

25. Material and methods are available as supplementary materials.

26. Z. Peng, R. Wang, L. Liu, H. Wu, Exploring urban spatial features of COVID-19 transmission in Wuhan based on social media data. *ISPRS Int. J. Geoinf.* **9**, 402 (2020). doi:10.3390/ijgi9060402

27. WorldPop, "WorldPop: Open spatial demographic data and research" (2020); http://worldpop.org.

28. A. J. Tatem, WorldPop, open data for spatial demography. *Sci. Data* **4**, 170004 (2017). doi:10.1038/sdata.2017.4 Medline

29. M. O'Driscoll, G. Ribeiro Dos Santos, L. Wang, D. A. T. Cummings, A. S. Azman, J. Paireau, A. Fontanet, S. Cauchemez, H. Salje, Age-specific mortality and immunity patterns of SARS-CoV-2. *Nature* **590**, 140–145 (2021). doi:10.1038/s41586-020-2918-0 Medline

30. A. Rambaut, E. C. Holmes, Á. O'Toole, V. Hill, J. T. McCrone, C. Ruis, L. du Plessis, O. G. Pybus, A dynamic nomenclature proposal for SARS-CoV-2 lineages to assist genomic epidemiology. *Nat. Microbiol.* **5**, 1403–1407 (2020). doi:10.1038/s41564-020-0770-5 Medline

31. outbreak.info, "SARS-CoV-2 (hCoV-19) mutation reports: Lineage/mutation tracker" (outbreak.info, 2022); https://outbreak.info/situation-reports.

32. R. Lu, X. Zhao, J. Li, P. Niu, B. Yang, H. Wu, W. Wang, H. Song, B. Huang, N. Zhu, Y. Bi, X. Ma, F. Zhan, L. Wang, T. Hu, H. Zhou, Z. Hu, W. Zhou, L. Zhao, J. Chen, Y. Meng, J. Wang, Y. Lin, J. Yuan, Z. Xie, J. Ma, W. J. Liu, D. Wang, W. Xu, E. C. Holmes, G. F. Gao, G. Wu, W. Chen, W. Shi, W. Tan, Genomic characterisation and epidemiology of 2019 novel coronavirus: Implications for virus origins and receptor binding. *Lancet* **395**, 565–574 (2020). doi:10.1016/S0140-6736(20)30251-8 Medline

33. L. Li, L. Yang, H. Zhu, R. Dai, Explorative analysis of Wuhan Intra-urban human mobility using social media check-in data. *PLOS ONE* **10**, e0135286 (2015). doi:10.1371/journal.pone.0135286 Medline

34. D. Majra, J. Benson, J. Pitts, J. Stebbing, SARS-CoV-2 (COVID-19) superspreader events. *J. Infect.* **82**, 36–40 (2021). doi:10.1016/j.jinf.2020.11.021 Medline

35. Epoch Times, "[Exclusive] The secret of Wuhan Huanan Seafood Market testing" (Epoch Times, 2020); https://www.epochtimes.com/gb/20/5/31/n12150755.htm.

36. Wuhan Municipal Bureau of Landscape Architecture and Forestry, "Administrative penalties in 2019" (Wuhan Municipal Bureau of Landscape Architecture and Forestry, 2019); https://web.archive.org/web/20211117124950/http://ylj.wuhan.gov.cn/zwgk/zwxxgkzl_12298/cfqz/xzcf/202011/t20201110_1499879.shtml.

37. Y.-Z. Zhang, E. C. Holmes, A genomic perspective on the origin and emergence of SARS-CoV-2. *Cell* **181**, 223–227 (2020). doi:10.1016/j.cell.2020.03.035 Medline

38. J. E. Pekar, A. Magee, E. Parker, N. Moshiri, K. Izhikevich, J. L. Havens, K. Gangavarapu, L. M. Malpica Serrano, A. Crits-Christoph, N. L. Matteson, M. Zeller, J. I. Levy, J. C. Wang, S. Hughes, J. Lee, H. Park, M.-S. Park, K. Ching Zi Yan, R. T. Pin Lin, M. N. Mat Isa, Y. M. Noor, T. I. Vasylyeva, R. F. Garry, E. C. Holmes, A. Rambaut, M. A. Suchard,

K. G. Andersen, M. Worobey, J. O. Wertheim, “SARS-CoV-2 emergence very likely resulted from at least two zoonotic events” (Zenodo, 2022); https://zenodo.org/record/6291628#.YtbMInbMKUk.

39. N. Chen, M. Zhou, X. Dong, J. Qu, F. Gong, Y. Han, Y. Qiu, J. Wang, Y. Liu, Y. Wei, J. Xia, T. Yu, X. Zhang, L. Zhang, Epidemiological and clinical characteristics of 99 cases of 2019 novel coronavirus pneumonia in Wuhan, China: A descriptive study. *Lancet* **395**, 507–513 (2020). doi:10.1016/S0140-6736(20)30211-7 Medline

40. Z. Li, X. Guan, N. Mao, H. Luo, Y. Qin, N. He, Z. Zhu, J. Yu, Y. Li, J. Liu, Z. An, W. Gao, X. Wang, X. Sun, T. Song, X. Yang, M. Wu, X. Wu, W. Yao, Z. Peng, J. Sun, L. Wang, Q. Guo, N. Xiang, J. Liu, B. Zhang, X. Su, L. Rodewald, L. Li, W. Xu, H. Shen, Z. Feng, G. F. Gao, Antibody seroprevalence in the epicenter Wuhan, Hubei, and six selected provinces after containment of the first epidemic wave of COVID-19 in China. *Lancet Reg Health West Pac* **8**, 100094 (2021). doi:10.1016/j.lanwpc.2021.100094 Medline

41. Z. He, L. Ren, J. Yang, L. Guo, L. Feng, C. Ma, X. Wang, Z. Leng, X. Tong, W. Zhou, G. Wang, T. Zhang, Y. Guo, C. Wu, Q. Wang, M. Liu, C. Wang, M. Jia, X. Hu, Y. Wang, X. Zhang, R. Hu, J. Zhong, J. Yang, J. Dai, L. Chen, X. Zhou, J. Wang, W. Yang, C. Wang, Seroprevalence and humoral immune durability of anti-SARS-CoV-2 antibodies in Wuhan, China: A longitudinal, population-level, cross-sectional study. *Lancet* **397**, 1075–1084 (2021). doi:10.1016/S0140-6736(21)00238-5 Medline

42. E. C. Holmes, S. A. Goldstein, A. L. Rasmussen, D. L. Robertson, A. Crits-Christoph, J. O. Wertheim, S. J. Anthony, W. S. Barclay, M. F. Boni, P. C. Doherty, J. Farrar, J. L. Geoghegan, X. Jiang, J. L. Leibowitz, S. J. D. Neil, T. Skern, S. R. Weiss, M. Worobey, K. G. Andersen, R. F. Garry, A. Rambaut, The origins of SARS-CoV-2: A critical review. *Cell* **184**, 4848–4856 (2021). doi:10.1016/j.cell.2021.08.017 Medline

43. J. Pekar, M. Worobey, N. Moshiri, K. Scheffler, J. O. Wertheim, Timing the SARS-CoV-2 index case in Hubei province. *Science* **372**, 412–417 (2021). doi:10.1126/science.abf8003 Medline

44. L. Chang, W. Hou, L. Zhao, Y. Zhang, Y. Wang, L. Wu, T. Xu, L. Wang, J. Wang, J. Ma, L. Wang, J. Zhao, J. Xu, J. Dong, Y. Yan, R. Yang, Y. Li, F. Guo, W. Cheng, Y. Su, J. Zeng, W. Han, T. Cheng, J. Zhang, Q. Yuan, N. Xia, L. Wang, The prevalence of antibodies to SARS-CoV-2 among blood donors in China. *Nat. Commun.* **12**, 1383 (2021). doi:10.1038/s41467-021-21503-x Medline

45. L. Chang, L. Zhao, Y. Xiao, T. Xu, L. Chen, Y. Cai, X. Dong, C. Wang, X. Xiao, L. Ren, L. Wang, Serosurvey for SARS-CoV-2 among blood donors in Wuhan, China from September to December 2019. *Protein Cell* pwac013 (2019). 10.1093/procel/pwac013

46. L. Lu, R. S. Sikkema, F. C. Velkers, D. F. Nieuwenhuijse, E. A. J. Fischer, P. A. Meijer, N. Bouwmeester-Vincken, A. Rietveld, M. C. A. Wegdam-Blans, P. Tolsma, M. Koppelman, L. A. M. Smit, R. W. Hakze-van der Honing, W. H. M. van der Poel, A. N. van der Spek, M. A. H. Spierenburg, R. J. Molenaar, J. Rond, M. Augustijn, M. Woolhouse, J. A. Stegeman, S. Lycett, B. B. Oude Munnink, M. P. G. Koopmans, Adaptation, spread and transmission of SARS-CoV-2 in farmed minks and associated humans in the Netherlands. *Nat. Commun.* **12**, 6802 (2021). doi:10.1038/s41467-021-27096-9 Medline

47. H.-L. Yen, T. H. C. Sit, C. J. Brackman, S. S. Y. Chuk, H. Gu, K. W. S. Tam, P. Y. T. Law, G. M. Leung, M. Peiris, L. L. M. Poon, S. M. S. Cheng, L. D. J. Chang, P. Krishnan, D. Y. M. Ng, G. Y. Z. Liu, M. M. Y. Hui, S. Y. Ho, W. Su, S. F. Sia, K.-T. Choy, S. S. Y. Cheuk, S. P. N. Lau, A. W. Y. Tang, J. C. T. Koo, L. Yung, Transmission of SARS-CoV-2 (Variant Delta) from pet hamsters to humans and onward human propagation of the adapted strain: A case study. *Lancet* **399**, 1070–1078 (2022). doi:10.1016/S0140-6736(22)00326-9 Medline

48. M. Standaert, E. Dou, "In search for coronavirus origins, Hubei caves and wildlife farms draw new scrutiny," *The Washington Post*, 11 October 2021; https://www.washingtonpost.com/world/asia_pacific/china-covid-bats-caves-hubei/2021/10/10/082eb8b6-1c32-11ec-bea8-308ea134594f_story.html.

49. X.-D. Lin, W. Wang, Z.-Y. Hao, Z.-X. Wang, W.-P. Guo, X.-Q. Guan, M.-R. Wang, H.-W. Wang, R.-H. Zhou, M.-H. Li, G.-P. Tang, J. Wu, E. C. Holmes, Y.-Z. Zhang, Extensive diversity of coronaviruses in bats from China. *Virology* **507**, 1–10 (2017). doi:10.1016/j.virol.2017.03.019 Medline

50. Q. Li, L. Zhou, M. Zhou, Z. Chen, F. Li, H. Wu, N. Xiang, E. Chen, F. Tang, D. Wang, L. Meng, Z. Hong, W. Tu, Y. Cao, L. Li, F. Ding, B. Liu, M. Wang, R. Xie, R. Gao, X. Li, T. Bai, S. Zou, J. He, J. Hu, Y. Xu, C. Chai, S. Wang, Y. Gao, L. Jin, Y. Zhang, H. Luo, H. Yu, J. He, Q. Li, X. Wang, L. Gao, X. Pang, G. Liu, Y. Yan, H. Yuan, Y. Shu, W. Yang, Y. Wang, F. Wu, T. M. Uyeki, Z. Feng, Epidemiology of human infections with avian influenza A(H7N9) virus in China. *N. Engl. J. Med.* **370**, 520–532 (2014). doi:10.1056/NEJMoa1304617 Medline

51. B. Lin, M. L. Dietrich, R. A. Senior, D. S. Wilcove, A better classification of wet markets is key to safeguarding human health and biodiversity. *Lancet Planet. Health* **5**, e386–e394 (2021). doi:10.1016/S2542-5196(21)00112-1 Medline

52. T. M. Davies, J. C. Marshall, M. L. Hazelton, Tutorial on kernel estimation of continuous spatial and spatiotemporal relative risk. *Stat. Med.* **37**, 1191–1221 (2018). doi:10.1002/sim.7577 Medline

53. Data and code for: M. Worobey, J. I. Levy, L. Malpica Serrano, A. Crits-Christoph, J. E. Pekar, S. A. Goldstein, A. L. Rasmussen, M. U. G. Kraemer, C. Newman, M. P. G. Koopmans, M. A. Suchard, J. O. Wertheim, P. Lemey, D. L. Robertson, R. F. Garry, E. C. Holmes, A. Rambaut, K. G. Andersen, The Huanan Seafood Wholesale Market in Wuhan was the early epicenter of the COVID-19, Zenodo (2022); http://doi.org/10.5281/zenodo.6786454.

54. M. Bondarenko, D. Kerr, A. Sorichetta, A. Tatem, "Census/projection-disaggregated gridded population datasets for 189 countries in 2020 using Built-Settlement Growth Model (BSGM) outputs" (WorldPop, 2020).

55. M. L. Hazelton, T. M. Davies, Inference based on kernel estimates of the relative risk function in geographical epidemiology. *Biom. J.* **51**, 98–109 (2009). doi:10.1002/bimj.200810495 Medline

56. K. G. Andersen, A. Rambaut, W. I. Lipkin, E. C. Holmes, R. F. Garry, The proximal origin of SARS-CoV-2. *Nat. Med.* **26**, 450–452 (2020). doi:10.1038/s41591-020-0820-9 Medline

57. W. Wang, J.-H. Tian, X. Chen, R.-X. Hu, X.-D. Lin, Y.-Y. Pei, J.-X. Lv, J.-J. Zheng, F.-H. Dai, Z.-G. Song, Y.-M. Chen, Y.-Z. Zhang, Coronaviruses in wild animals sampled in and around Wuhan at the beginning of COVID-19 emergence. *Virus Evol.* **8**, veac046 (2022). doi:10.1093/ve/veac046 Medline

58. E. C. Holmes, A. Rambaut, K. G. Andersen, Pandemics: Spend on surveillance, not prediction. *Nature* **558**, 180–182 (2018). doi:10.1038/d41586-018-05373-w Medline

59. W.-H. Kong, Y. Li, M.-W. Peng, D.-G. Kong, X.-B. Yang, L. Wang, M.-Q. Liu, SARS-CoV-2 detection in patients with influenza-like illness. *Nat. Microbiol.* **5**, 675–678 (2020). doi:10.1038/s41564-020-0713-1 Medline

60. J. Tao, H. Gao, S. Zhu, L. Yang, D. He, Influenza versus COVID-19 cases among influenza-like illness patients in travelers from Wuhan to Hong Kong in January 2020. *Int. J. Infect. Dis.* **101**, 323–325 (2020). doi:10.1016/j.ijid.2020.09.1474 Medline

61. J. Bai, F. Shi, J. Cao, H. Wen, F. Wang, S. Mubarik, X. Liu, Y. Yu, J. Ding, C. Yu, The epidemiological characteristics of deaths with COVID-19 in the early stage of epidemic in Wuhan, China. *Glob. Health Res. Policy* **5**, 54 (2020). doi:10.1186/s41256-020-00183-y Medline

62. Q. Li, X. Guan, P. Wu, X. Wang, L. Zhou, Y. Tong, R. Ren, K. S. M. Leung, E. H. Y. Lau, J. Y. Wong, X. Xing, N. Xiang, Y. Wu, C. Li, Q. Chen, D. Li, T. Liu, J. Zhao, M. Liu, W. Tu, C. Chen, L. Jin, R. Yang, Q. Wang, S. Zhou, R. Wang, H. Liu, Y. Luo, Y. Liu, G. Shao, H. Li, Z. Tao, Y. Yang, Z. Deng, B. Liu, Z. Ma, Y. Zhang, G. Shi, T. T. Y. Lam, J. T. Wu, G. F. Gao, B. J. Cowling, B. Yang, G. M. Leung, Z. Feng, Early transmission dynamics in Wuhan, China, of novel coronavirus-infected pneumonia. *N. Engl. J. Med.* **382**, 1199–1207 (2020). doi:10.1056/NEJMoa2001316 Medline

63. Y. Jia, Z. Zheng, Q. Zhang, M. Li, X. Liu, Associations of spatial aggregation between neighborhood facilities and the population of age groups based on points-of-interest data. *Sustainability (Basel)* **12**, 1692 (2020). doi:10.3390/su12041692

64. F. Maussion, TimoRoth, R. Bell, F. Li, J. Landmann, M. Dusch, “fmaussion/salem: v0.3.7” (Zenodo, 2021); https://zenodo.org/record/596573.

65. D. Wang, B. Hu, C. Hu, F. Zhu, X. Liu, J. Zhang, B. Wang, H. Xiang, Z. Cheng, Y. Xiong, Y. Zhao, Y. Li, X. Wang, Z. Peng, Clinical characteristics of 138 hospitalized patients with 2019 novel coronavirus-infected pneumonia in Wuhan, China. *JAMA* **323**, 1061–1069 (2020). doi:10.1001/jama.2020.1585 Medline

66. D. Wang, J. Cai, T. Shi, Y. Xiao, X. Feng, M. Yang, W. Li, W. Liu, L. Yu, Z. Ye, T. Xu, J. Ma, M. Li, W. Chen, Epidemiological characteristics and the entire evolution of coronavirus disease 2019 in Wuhan, China. *Respir. Res.* **21**, 257 (2020). doi:10.1186/s12931-020-01525-7 Medline

67. F. Li, Y.-Y. Li, M.-J. Liu, L.-Q. Fang, N. E. Dean, G. W. K. Wong, X.-B. Yang, I. Longini, M. E. Halloran, H.-J. Wang, P.-L. Liu, Y.-H. Pang, Y.-Q. Yan, S. Liu, W. Xia, X.-X. Lu, Q. Liu, Y. Yang, S.-Q. Xu, Household transmission of SARS-CoV-2 and risk factors for susceptibility and infectivity in Wuhan: A retrospective observational study. *Lancet Infect. Dis.* **21**, 617–628 (2021). doi:10.1016/S1473-3099(20)30981-6 Medline

68. K. Wernike, A. Aebischer, A. Michelitsch, D. Hoffmann, C. Freuling, A. Balkema-

Buschmann, A. Graaf, T. Müller, N. Osterrieder, M. Rissmann, D. Rubbenstroth, J. Schön, C. Schulz, J. Trimpert, L. Ulrich, A. Volz, T. Mettenleiter, M. Beer, Multi-species ELISA for the detection of antibodies against SARS-CoV-2 in animals. *Transbound. Emerg. Dis.* **68**, 1779–1785 (2021). doi:10.1111/tbed.13926 Medline

69. X. Zhao, D. Chen, R. Szabla, M. Zheng, G. Li, P. Du, S. Zheng, X. Li, C. Song, R. Li, J.-T. Guo, M. Junop, H. Zeng, H. Lin, Broad and differential animal angiotensin-converting enzyme 2 receptor usage by SARS-CoV-2. *J. Virol.* **94**, e00940-20 (2020). doi:10.1128/JVI.00940-20 Medline

70. A. Z. Mykytyn, M. M. Lamers, N. M. A. Okba, T. I. Breugem, D. Schipper, P. B. van den Doel, P. van Run, G. van Amerongen, L. de Waal, M. P. G. Koopmans, K. J. Stittelaar, J. M. A. van den Brand, B. L. Haagmans, Susceptibility of rabbits to SARS-CoV-2. *Emerg. Microbes Infect.* **10**, 1–7 (2021). doi:10.1080/22221751.2020.1868951 Medline

71. P. Chen, J. Wang, X. Xu, Y. Li, Y. Zhu, X. Li, M. Li, P. Hao, Molecular dynamic simulation analysis of SARS-CoV-2 spike mutations and evaluation of ACE2 from pets and wild animals for infection risk. *Comput. Biol. Chem.* **96**, 107613 (2022). doi:10.1016/j.compbiolchem.2021.107613 Medline

72. V. L. Hale, P. M. Dennis, D. S. McBride, J. M. Nolting, C. Madden, D. Huey, M. Ehrlich, J. Grieser, J. Winston, D. Lombardi, S. Gibson, L. Saif, M. L. Killian, K. Lantz, R. M. Tell, M. Torchetti, S. Robbe-Austerman, M. I. Nelson, S. A. Faith, A. S. Bowman, SARS-CoV-2 infection in free-ranging white-tailed deer. *Nature* **602**, 481–486 (2022). doi:10.1038/s41586-021-04353-x Medline

73. S. M. Porter, A. E. Hartwig, H. Bielefeldt-Ohmann, A. M. Bosco-Lauth, J. J. Root, Susceptibility of wild canids to severe acute respiratory syndrome coronavirus 2 (SARS-CoV-2). bioRxiv 478082 [Preprint] (2022); https://doi.org/10.1101/2022.01.27.478082.

74. L. Jemeršić, I. Lojkić, N. Krešić, T. Keros, T. A. Zelenika, L. Jurinović, D. Skok, I. Bata, J. Boras, B. Habrun, D. Brnić, Investigating the presence of SARS CoV-2 in free-living and captive animals. *Pathogens* **10**, 635 (2021). doi:10.3390/pathogens10060635 Medline

75. C. S. Lupala, V. Kumar, X.-D. Su, C. Wu, H. Liu, Computational insights into differential interaction of mammalian angiotensin-converting enzyme 2 with the SARS-CoV-2 spike receptor binding domain. *Comput. Biol. Med.* **141**, 105017 (2022). doi:10.1016/j.compbiomed.2021.105017 Medline

76. C. D. Eckstrand, T. J. Baldwin, K. A. Rood, M. J. Clayton, J. K. Lott, R. M. Wolking, D. S. Bradway, T. Baszler, An outbreak of SARS-CoV-2 with high mortality in mink (Neovison vison) on multiple Utah farms. *PLOS Pathog.* **17**, e1009952 (2021). doi:10.1371/journal.ppat.1009952 Medline

77. N. Oreshkova, R. J. Molenaar, S. Vreman, F. Harders, B. B. Oude Munnink, R. W. Hakze-van der Honing, N. Gerhards, P. Tolsma, R. Bouwstra, R. S. Sikkema, M. G. Tacken, M. M. de Rooij, E. Weesendorp, M. Y. Engelsma, C. J. Bruschke, L. A. Smit, M. Koopmans, W. H. van der Poel, A. Stegeman, SARS-CoV-2 infection in farmed minks, the Netherlands, April and May 2020. *Euro Surveill.* **25**, (2020). doi:10.2807/1560-7917.ES.2020.25.23.2001005 Medline

78. A. S. Hammer, M. L. Quaade, T. B. Rasmussen, J. Fonager, M. Rasmussen, K. Mundbjerg,

L. Lohse, B. Strandbygaard, C. S. Jørgensen, A. Alfaro-Núñez, M. W. Rosenstierne, A. Boklund, T. Halasa, A. Fomsgaard, G. J. Belsham, A. Bøtner, SARS-CoV-2 transmission between mink (*Neovison vison*) and humans, Denmark. *Emerg. Infect. Dis.* **27**, 547–551 (2021). doi:10.3201/eid2702.203794 Medline

79. Z. Song, L. Bao, W. Deng, J. Liu, E. Ren, Q. Lv, M. Liu, F. Qi, T. Chen, R. Deng, F. Li, Y. Liu, Q. Wei, H. Gao, P. Yu, Y. Han, W. Zhao, J. Zheng, X. Liang, F. Yang, C. Qin, Integrated histopathological, lipidomic, and metabolomic profiles reveal mink is a useful animal model to mimic the pathogenicity of severe COVID-19 patients. *Signal Transduct. Target. Ther.* **7**, 29 (2022). doi:10.1038/s41392-022-00891-6 Medline

80. H.-L. Zhang, Y.-M. Li, J. Sun, Y.-Y. Zhang, T.-Y. Wang, M.-X. Sun, M.-H. Wang, Y.-L. Yang, X.-L. Hu, Y.-D. Tang, J. Zhao, X. Cai, Evaluating angiotensin-converting enzyme 2-mediated SARS-CoV-2 entry across species. *J. Biol. Chem.* **296**, 100435 (2021). doi:10.1016/j.jbc.2021.100435 Medline

81. K. L. Stout, "'Wuhan SARS': Tracing the origin of the new virus to China's wild animal markets" (YouTube, 2020); https://www.youtube.com/watch?v=Je0_U2ym_r0.
